# Examining Complexity across Domains: Relating Subjective and Objective Measures of Affective Environmental Scenes, Paintings and Music

**DOI:** 10.1371/journal.pone.0072412

**Published:** 2013-08-16

**Authors:** Manuela M. Marin, Helmut Leder

**Affiliations:** Department of Basic Psychological Research and Research Methods, University of Vienna, Vienna, Austria; UC Davis School of Medicine, United States of America

## Abstract

Subjective complexity has been found to be related to hedonic measures of preference, pleasantness and beauty, but there is no consensus about the nature of this relationship in the visual and musical domains. Moreover, the affective content of stimuli has been largely neglected so far in the study of complexity but is crucial in many everyday contexts and in aesthetic experiences. We thus propose a cross-domain approach that acknowledges the multidimensional nature of complexity and that uses a wide range of objective complexity measures combined with subjective ratings. In four experiments, we employed pictures of affective environmental scenes, representational paintings, and Romantic solo and chamber music excerpts. Stimuli were pre-selected to vary in emotional content (pleasantness and arousal) and complexity (low versus high number of elements). For each set of stimuli, in a between-subjects design, ratings of familiarity, complexity, pleasantness and arousal were obtained for a presentation time of 25 s from 152 participants. In line with Berlyne’s collative-motivation model, statistical analyses controlling for familiarity revealed a positive relationship between subjective complexity and arousal, and the highest correlations were observed for musical stimuli. Evidence for a mediating role of arousal in the complexity-pleasantness relationship was demonstrated in all experiments, but was only significant for females with regard to music. The direction and strength of the linear relationship between complexity and pleasantness depended on the stimulus type and gender. For environmental scenes, the root mean square contrast measures and measures of compressed file size correlated best with subjective complexity, whereas only edge detection based on phase congruency yielded equivalent results for representational paintings. Measures of compressed file size and event density also showed positive correlations with complexity and arousal in music, which is relevant for the discussion on which aspects of complexity are domain-specific and which are domain-general.

## Introduction

Complexity has been widely studied in psychology and related disciplines because of its relevance to humans’ relations with their environments [1,2]. These disciplines have focused on the behavioral outcomes of sensory, cognitive and affective responses to stimuli varying in perceived complexity [3,6]. Understanding the impact of such collative stimulus dimensions as complexity, uncertainty and novelty, on hedonic value (e.g., preference, pleasingness and beauty) has been of paramount importance in empirical aesthetics, which primarily aims to study aesthetic preferences, mostly owing to Daniel Berlyne’s contributions [7,10]. Research in light of Berlyne’s *New Experimental Aesthetics* has been largely motivated by the question of what determines humans’ preferences for certain kinds of stimuli over others. Perceived complexity has been reliably identified as a key determinant of hedonic value [11], and has therefore been included in current frameworks for the study of aesthetic experiences [12,15].

Research on complexity in the field of empirical aesthetics has been marred by contradictory findings, which may be due to several theoretical and experimental shortcomings. For example, the multidimensionality of complexity [16] is a major issue that needs to be controlled for in research designs. In this study, we focused on the number and variety of elements present in a visual and auditory scene. This dimension was found to be the strongest determinant of subjective complexity, more than organization or symmetry [16,17]. Moreover, researchers have mostly focused on subtle manipulations of stimulus complexity and neglected the emotional contents of stimuli. This has led to a restricted, ecologically invalid way of studying the impact of complexity in aesthetic experiences, given the crucial role of emotions in such experiences. In addition, it is still not known whether the relationship between complexity and hedonic value is domain-specific or domain-general.

Here, we thus studied the effects of stimulus complexity on pleasantness (taken as a measure of hedonic value) by comparatively investigating large sets of visual and musical stimuli within the context of Russell’s circumplex model of affect [18]. Moreover, we also examined the relation between a set of algorithms (including both previously used algorithms and new ones) to measure objective complexity in two kinds of affective visual stimuli varying in aesthetic quality and subjectively rated complexity. Our findings revealed that their performance depends in part on the stimulus type. In addition, our findings demonstrated that analogous types of algorithms developed for the auditory domain can be fruitful for the study of musical complexity and its relation to arousal and pleasantness. Finally, our analyses accounted for gender effects reported in response to affective visual [19] and musical stimuli [20]. In summary, we introduced a comprehensive approach to the study of subjective complexity, which not only comprised recent developments in measuring objective complexity but also crossmodal comparisons based on ecologically valid stimuli.

### Berlyne’s Collative-motivation Model and Divergent Research Findings

Berlyne’s model predicts that people will generally prefer stimuli of intermediate complexity to simple and highly complex ones under normal arousal conditions [7,9]. This preference for intermediate levels of complexity gives rise to an optimal level of arousal, considered as an intervening variable, experienced as hedonically positive. The collative properties of a stimulus are, in Berlyne’s model [9], the main determinants of arousal, though psychophysical (e.g., brightness, saturation, intensity) and ecological variables (e.g., innate or learned signal value, meaningfulness) also add to stimuli’s arousal potential. Despite empirical evidence for an inverted U-relationship between a stimulus’ perceived complexity and various measures of hedonic value in the visual [21,27] and musical domains [28,35], a considerable amount of counterevidence has also accumulated. These findings have either mostly revealed a linear relationship between perceived complexity and a specific measure of hedonic value or no clear relationship between these variables, both in the visual [16,36,41] and musical domains [42,47].

The discrepancy between findings of the relationship between perceived complexity and hedonic value in the visual and musical domains may not be explicable solely by the wide range of stimuli types employed in these experiments [16,44], which ranged from simple geometric forms and random shapes to artistic stimuli and landscapes in the visual domain, and from sequences of pure tones, melodies, chord progressions to music of different styles in the musical domain. Instead, the limited number of participants and stimuli used in several of the reported studies may have additionally affected the results. Moreover, the ecological validity of these materials may also play a crucial role in explaining the divergent findings [16,44]. The use of highly artificial stimuli, possibly due to the fact that a stringent manipulation of objective complexity has been deemed necessary by most researchers, is rather surprising considering that Berlyne is largely regarded as a motivational theorist [48], whose theoretical framework is primarily concerned about how humans explore their environment and display curiosity [7]. In addition, the different conceptions of complexity within psychology [49] and the related measurements and manipulations thereof may constitute another important factor explaining the discrepancy of the current research findings [16].

There is abundant empirical evidence for a multidimensional nature of complexity of visual [16,17] and musical [50] materials. Subjective visual complexity has been shown to be determined by stimulus features such as the number of elements, their organization and symmetry [16]. In a meta-analysis of studies exploring the relationship between complexity and hedonic value based on different definitions of visual complexity, Nadal et al. [16] suggested that manipulations of complexity on the basis of the number of elements present in a stimulus yielded a linearly increasing relationship between complexity and beauty, whereas varying the organization of elements showed an U-shaped or descending relationship, and manipulations along the dimension of symmetry an inverted U-shape relationship. However, regarding a comprehensive theory of aesthetic preference, other determinants besides complexity, such as stimulus prototypicality [51,52], expertise [44,53,55], familiarity [56,58], personality traits [59,62] and situational influences [63] may also have confounded the investigations on the relationship between subjective complexity, arousal and hedonic value.

### Introducing Emotion to the Study of Complexity

The anomalous empirical findings in relation to Berlyne’s collation-arousal model have led to serious criticism on theoretical grounds. For example, Berlyne’s disregard of the dawn of cognitive psychology [64] made it impossible to integrate the interaction of thoughts and emotions in his framework of aesthetic experience. Another criticism concerns the disinterest in the diversity of emotions and their likely role in aesthetic experience. It is widely accepted that emotions play a fundamental role, for example, in the experience of visual art [65] and music [66], and thus it is not surprising that an affective approach to the study of aesthetic experience has been recently proposed [67,70]. For instance, the application of appraisal theories of emotion (for a review, see 71), which posit that subjective cognitive appraisals of events are the source of a wide range of possible emotions, may help account for the variability of emotions commonly experienced in response to artistic stimuli [72,73]. In contrast, Berlyne’s collation-arousal model is restricted to arousal shifts and concomitant enjoyment, interest or aversion in response to a stimulus, yielding either simple positive or negative affective states. Another difference between Berlyne’s theory and appraisal theories was pointed out by Silvia [70], who argues that objective stimulus features and collative properties of a stimulus are not sufficient to create an emotion. Nevertheless, it needs to be stated that Berlyne’s theory implies that the subjective experience of stimulus features modulates hedonic value, not the objectively measurable features of a stimulus [8,21]. In other words, with regard to subjective evaluations of stimulus features the two contrasting theories are not alien from each other.

Despite these initial efforts to introduce emotion to the study of aesthetic experiences induced by visual and auditory stimuli, previous research on complexity did not use stimuli that are strongly emotionally expressive, and even explicitly avoided using them [16,74]. To be specific, the stimuli under investigation were not selected within the context of current emotion models or on the basis of their degree of emotionality, but primarily on the basis of their varying degree of complexity [6,75,76]. We consider this as an important shortcoming because it is known, for instance, that the emotional content of stimuli modulates even the early neural processing of visual features [77,78].

The concept of arousal may be helpful in bridging the gap between emotion psychology and the study of stimulus complexity in the field of empirical aesthetics, particularly since arousal is an essential dimension in several emotion models [9,18,79,80]. However, studies in the tradition of Berlyne usually do not consider arousal as an independent variable, and moreover they rarely collect subjective arousal ratings or physiological arousal measures (but see 31,37,38,81,82). In order to incorporate arousal conceptually and empirically in the study of complexity, we propose to examine complexity within the context of Russell’s circumplex model of affect [18]. The model considers arousal and pleasantness as two independent dimensions of affect, defining a wide range of different emotions. This two-dimensional model is highly suitable for crossmodal comparisons due to its simplicity and wide application in the visual and musical domains [83].

Gender effects in emotional processing are commonly reported and their underlying causes are manifold [84]. Specifically, gender effects with regard to emotional processing of visual [19,85,88] and musical [20] materials have been widely documented. Furthermore, subjective complexity judgments of photographs of fruits and vegetables have recently been found to be affected by gender [89]. Research on the perception of odors also revealed effects of complexity on preference that differed in females and males [90,91]. Therefore, we decided to consider the gender of the participant in the current research design and provide results for both genders separately.

Emotional responses are usually studied within the context of short presentation times. However, there is evidence that visual art is often experienced for much longer. For example, in a museum context Smith and Smith [92] reported an average viewing time of 27.2 s. Consequently, in order to enhance the ecological validity of our results and to study real aesthetic episodes, we chose presentation durations of 25 s in all four experiments. This also allowed for a fair comparison between the visual and musical domains since music is a dynamic stimulus that unfolds over time. Presentation durations of approximately 30 s are commonly used in research on musical emotions [83,93] and thus seemed to be appropriate for the purposes of the current study.

### Computational Measures of Complexity

Which computational methods capture variations in complexity in a way that might be representative of how humans perceive complexity? The study of subjective complexity and its relationship to aesthetic experience has profited from work in the field of digital image processing and recent advances in finding automated measures of visual complexity [74,94,95]. These developments go beyond the initial mathematical approach of calculating objective complexity by considering the number of elements (lines and angles) and their heterogeneity in an additive way [96,98]. Instead, the application of various image compression techniques [99,103] and edge detection algorithms [74,100,103,104] has proved to be a reliable correlate of subjective visual complexity. Importantly, these measures were not developed by computer scientists to predict subjective complexity in the first place, but were only currently successfully applied to various types of visual stimuli by psychologists. The ratio between the original and the compressed file sizes of marine electronic charts and radar images [101,105], icons [103], line drawings [99,103] environmental scenes [74,104] and a wide range of artistic works [74] have been shown to be positively correlated with ratings of subjective complexity. It needs to be stated plainly that these types of stimuli were not selected within the context of a specific emotion model. In addition, only Forsythe et al. [74] have used compressed file size as a measure of complexity to test Berlyne’s inverted U-shape hypothesis so far.

In the few studies that applied compression algorithms as objective measures of complexity to images, the file sizes of JPEG (Joint Photographic Expert Group) and GIF (Graphics Interchange Format) compression formats have yielded moderate correlations with subjective complexity judgments. In these studies, the stimulus presentation times [74,95,101,102,104] varied from several seconds to an unlimited exposure time in sorting tasks, suggesting that the correlation between compressed file size and subjective complexity may be independent of presentation time. Furthermore, Forsythe et al. [102] found that familiarity does not interact with objective complexity as measured by compressed file size and perimeter detection, which makes it a more reliable measure than subjective complexity ratings which are usually influenced by familiarity with the stimuli [106,107].

Donderi [4] explains that the success of image compression techniques to predict subjective complexity can be understood within the context of Algorithmic Information Theory (AIT) (for a review, see 108,109) which combines information-theory with the theory of computation (for a review on the Turing machine see 110): “Algorithmic complexity is defined in terms of the length of the shortest algorithm in any programming language, which computes a particular binary string” [49], p. 6. In principle, data compression algorithms analyze the visual information of an image, as described by a bit string, in order to compress it to the extent that makes a valid reproduction of the original input possible [4]. The size of the resulting compressed data file correlates positively with the complexity of the input image. Simple images contain more redundant information that can be represented by a shorter string of bits, yielding a smaller file size than more complex images.

Besides the use of data compression, various edge detection measures developed in the field of image statistics have been shown to be another reliable way of measuring holistic complexity in the visual domain [74,95,100,102,104]. Edge detection algorithms, such as perimeter detection and Canny edge detection, detect changes in intensity at an image’s edges. The more edges an image has, the higher the level of perceived complexity is [102,103]. Perimeter detection, a contour-based and a global measure of shape [111], has specifically yielded reliable - though moderate - correlations with subjective complexity across different stimulus sets [74,102,103]. Alternative approaches to edge detection include the analysis of root mean square (RMS) contrast, the standard deviation of the pixel intensities, which was recently applied to a set of day- and nightscapes by Cavalcante et al. [104]. The presence of high-contrast features is indicated by a high mean RMS contrast value, yielding a positive correlation with subjective complexity. The correlations between different measures of RMS contrast and subjective complexity were stronger (*r* ~.60) than the one for JPEG file size (*r* = .36). Thus, applications of RMS contrast measures to other types of visual stimuli seem promising.

Music is another affective domain that is widely studied within experimental psychology. The study of subjective complexity and objective complexity measures, however, has hardly received any attention in the field of music psychology [112], although research on musical expectation and its relation to complexity is flourishing [76,113,114]. In contrast to several recent reports of a positive association between compressed file size and subjective complexity in the visual domain, we are unaware of any studies using compression algorithms and related file sizes to predict subjective complexity in the musical domain. For example, Streich [50] modeled subjective musical complexity on the basis of twelve predictors extracted from audio, but this model did not include compression file size. Streich’s model comprised four measures relating to the dynamic and spatial properties of an audio excerpt, one measure of timbral complexity, three measures of tonal strength, as well as four measures of rhythmic complexity. More recently, Mauch and Levy [115] proposed to objectively measure musical complexity by means of a structural change algorithm applied to changes in harmony, rhythm and timbre. Results of an internet-based experiment indicated that around 61.4% of the listeners agreed with the automated analysis, which is similar to the performance of Streich’s model [50]. Nevertheless, it was shown earlier that compression-based approaches to classify MIDI files were successful in differentiating between musical works of different periods and between solo piano music by different composers of different periods [116,117]. Based on these findings, and the results reported above on studies using data compression in the visual domain, it can be surmised that the ratio between the original and the compressed file size may also be a significant predictor of subjective complexity of musical materials.

### Overview of the Present Experiments

We investigated subjective and objective complexity in the visual and musical domains in a series of four experiments. The main goals of our approach were threefold: to compare the relationships between subjective complexity, felt arousal and pleasantness (by controlling for effects of familiarity) in large sets of visual and musical stimuli selected on the basis of Russell’s circumplex model of affect [18]; to relate subjective complexity to measures of objective complexity; and to gain further insights into the similarities and difference between the perception of environmental scenes and art images. Our approach is characterized by the following decisions: First, pleasantness was chosen as a measure of hedonic value and arousal was not only considered as an unmeasured intervening variable within the framework of Berlyne’s collative-motivation model. Instead, variations in arousal were inherent in the design of our experiments because we aimed at selecting representative stimuli that covered the arousal-pleasantness emotion space of a particular stimulus type as much as possible. Second, complexity was manipulated by pre-selecting stimuli that varied in the number of elements present in a visual or musical scene. Third, in order to make valid comparisons between the perception of environmental scenes and visual art possible, both types of stimuli were chosen to contain similar semantic contents. Fourth, we also explored the mediating role of arousal in the complexity-pleasantness relationship in the context of Berlyne’s theory [9]. For example, Vettehen et al. [118] recently employed mediation analysis to address this question in a study on the effect of sensationalism on liking of television news stories. In this relationship, arousal was identified as a mediator.

In the field of image statistics, direct comparisons between image properties of environmental scenes and visual art have become popular because they may offer insights into the nature of artistic stimuli [119,121]. We were following this trend by comparing the performance of the very same set of objective complexity measures on two stimulus sets varying in motivational relevance and artistic quality (Experiments 1 and 2). Specifically, we compared the performance of objective measures related to object recognition processes (i.e., edge detection measures) and measures that capture visual information in a more abstract way (i.e., compressed file size) as approximations to human subjective ratings. In this study, we were particularly interested in the practical means of measuring objective complexity for the purpose of stimulus selection. In line with this, we neither primarily aimed at a comprehensive model of subjective complexity based on objective measures nor at an in-depth discussion of the performance of each objective measure with regard to aspects of perceptual and cognitive processing of complexity.

The goals of Experiments 1 and 2 were to compare the inter-relationships between subjective ratings of familiarity, complexity, pleasantness and arousal of affective environmental scenes and representational paintings, and further, to compare the performance of the very same set of objective measures of complexity. In Experiment 1, stimuli consisted of affective environmental scenes selected from the International Affective Picture System (IAPS) developed by Lang, Bradley, and Cuthbert [122], a stimuli database widely used for visual emotion induction. In Experiment 2, we studied pre-selected affective representational paintings with similar semantic contents as the pictures used in Experiment 1. Since most previous studies only focused on a few measures of objective complexity, we aimed at assessing the performance of a wide range of measures previously reported to be successful in predicting subjective complexity (i.e., JPEG and GIF compression file size, perimeter detection, Canny edge detection, RMS contrast). Moreover, we extended this set of measures by including several potentially new measures of objective complexity, such as PNG (Portable Network Graphics) and TIFF (Tagged Image File Format) compression formats, measures of edge detection based on phase congruency [123], and the entropy of the image intensity histogram of a grayscale image [124]. It is also important to note that since it was already demonstrated earlier that compression file size is an indicator of subjective complexity [74,105], we selected pictures in JPEG format rather than uncompressed pictures as a starting point for all further transformations and analyses. This approach extends the implications and applicability of the current research findings since JPEG pictures are more easily accessible to the research community compared to (scans of) pictures in uncompressed formats.

For Experiments 1 and 2, we hypothesized that subjective complexity and arousal would be positively associated in both types of visual stimuli [9,21]. Furthermore, based on research by Nadal et al. [16], we surmised that subjective complexity and pleasantness would be linearly and positively associated because we mainly varied complexity by the number of elements present in a visual scene. In other words, we did not predict an inverted U-shape relationship between these variables as proposed by Berlyne [9]. Nevertheless, we predicted that the effect of complexity on pleasantness would be mediated by arousal, as proposed by Berlyne [9]. We also hypothesized that JPEG and GIF compression file sizes [101,102,104], perimeter detection [74,103], Canny edge detection [102] and RMS contrast measures [104] would yield a moderate positive relationship with subjective complexity if the image size was held constant, and that these objective measures would not correlate with reported familiarity [102]. Last, we predicted that measures of objective complexity would correlate positively with pleasantness [74].

Another major goal of the study was to show the fruitfulness of a comparative approach to the study of emotion and complexity by demonstrating the application of objective measures of complexity to the musical domain (Experiments 3 and 4) by using analogous measures to those used in the visual domain (Experiments 1 and 2). As such, this paper contributes to a wider theoretical discussion on the relationships between subjective complexity, arousal and pleasantness (i.e., one measure of hedonic value), and the use of objective measures of complexity in the affective musical and visual domains. In Experiment 3, we investigated subjective responses to a stimulus set recently developed by Marin et al. [83]. These musical stimuli represented one musical genre (Romantic solo piano music of the 19^th^ century) and were evaluated within the context of Russell’s circumplex model of affect [18]. In Experiment 4, we additionally varied complexity in a similar vein as we did in the visual domain (in Experiments 1 and 2) by changing the number of instruments audible in the musical excerpts, i.e., one versus three instruments. For this purpose, half of the stimuli were selected based on results of Experiment 3, and the other half consisted of piano trio excerpts of the same musical period. The hypotheses with regard to the inter-relationships between subjective complexity, pleasantness and arousal were identical to those of Experiments 1 and 2.

Since no study has reported on the use of compression file size as a measure of subjective complexity in the musical domain, we applied various audio compression formats to uncompressed WAV (Waveform Audio File Format) files in a first step. The auditory domain is prone to such an investigation because compression of audio files is a common and easily accessible tool. Moreover, analyses of acoustic and musical properties of audio signals relevant to subjective complexity have been made possible by recent developments in the field of music information retrieval [50,115]. Therefore, in analogy to edge detection algorithms and their application to predict subjective complexity in the visual domain, it was decided to analyze the event density per second of each musical excerpt by means of the MIRtoolbox [125], a collection of functions written in Matlab. Event density analyzes the overall amount of simultaneous (melodic, harmonic and rhythmic) events in a musical excerpt that can be perceived by a musical listener. This measure was not included in the models of musical complexity developed by Streich [50] and Mauch and Levy [115]. However, it can be conjectured that musical excerpts containing fewer musical events are judged as less complex than those with a higher number of events, and similarly, that compressed file size is a predictor of subjective complexity as previously observed in the visual domain.

## Experiment 1

### Methods

#### Ethics Statement

All four experiments reported in this article were conducted in accordance with the Declaration of Helsinki (revised 1983) and local guidelines of the Faculty of Psychology, University of Vienna. According to the Austrian Universities Act 2002 (UG2002) which held at the time the study was carried out, only medical universities were required to appoint ethics committees for clinical tests, application of medical methods, and applied medical research. Therefore, no ethical approval was required for the present study. Written informed consent was given by all participants who could withdraw at any time during the experiment without further consequences.

#### Participants

Thirty-six German-speaking psychology students (18 males, 23.9 ± 4.9 years, age range 21-42 years; 18 females, 22.6 ± 4.7 years, age range 20-41 years) participated in exchange for course credit. All participants had normal or corrected-to-normal visual acuity. These participants did not take part in any other experiment of this study.

#### Materials

Pictures were chosen from the International Affective Picture System (IAPS) [122], a standardized picture system of colored photographs widely used in experimental psychology. Ninety-six colored pictures of realistic natural scenes were pre-selected on the basis of their standardized values of arousal and pleasantness to vary in hedonic content (high vs. low-arousing and unpleasant vs. pleasant). In addition, pictures were also chosen on the basis of their degree of visual complexity (figure-ground composition vs. complex scene), for which no standardized values were available. However, the classification of pictures into figure-ground compositions and complex scenes was guided by the results reported in studies on visual complexity and emotion using IAPS pictures [3,126]. Following Russell’s circumplex model of affect [18], this pre-selection led to 24 pictures of specific hedonic contents (either low-arousing pleasant, high-arousing pleasant, low-arousing unpleasant, or high-arousing unpleasant). Half of the pictures were chosen to depict relatively simple figure-ground compositions (i.e., one figure with a uniform background), and the other half depicted complex scenes with several objects and a more varied background. The semantic content of these pictures varied largely, ranging from animals, plants, landscapes and food to human beings in everyday life scenes. Pictures showing erotic scenes, brand names and strong mutilation were not included in the stimulus set (see Supporting Information Stimulus List S1). All pictures were in landscape format (1024 x 768 pixels) and saved in their original JPEG format.

#### Procedure

Participants were tested either individually or in pairs separated by a wall in a quiet room. After signing the informed consent form, participants completed two practice trials in which they were familiarized with the task. Each trial was announced by a sentence appearing in the middle of the screen (Samsung SyncMaster S2443BW, 24-inch) and the picture followed after 5 s, displayed on a black background at a size of 33.90 x 25.50 cm. Participants, sitting 60-70 cm away from the screen, were instructed to look at the picture for the entire presentation duration of 25 s. After the picture had disappeared, the first out of four rating scales was displayed on the screen. This procedure was chosen to make possible a comparison between the visual and musical modalities because, due to the dynamic nature of music, global ratings could only be obtained after participants had listened to the excerpts. Participants indicated their choice by a mouse click and the next rating scale appeared immediately after. All pictures were rated on a seven-point Likert scale in terms of familiarity (from “unfamiliar” to “very familiar”), complexity (from “very simple” to “very complex”), pleasantness (from “very unpleasant” to “very pleasant”) and arousal (from “very calm” to “very excited”). The order of the four ratings was the same for each participant and stimulus and the specific instructions said: “Please rate your familiarity with the contents of the picture,” “Please rate your felt complexity of the picture,” “Please rate the degree of pleasantness of your emotional experience,” and “Please rate your felt arousal”. From another recent experiment (unpublished data) involving the same stimuli sets as those used in the current Experiments 1 and 2, we found that the order of ratings (familiarity-complexity-pleasantness-arousal versus pleasantness-arousal-complexity-familiarity) did not affect the inter-relationships of the four variables under investigation. Importantly, participants were instructed to report their subjective feelings rather than the perceived emotional contents of the pictures (felt vs. perceived emotion, see 127) and to give all ratings spontaneously (although they were informed that there were no time constraints).

The pictures were presented in four blocks and the order of the blocks was randomized across participants. Within each block, 24 pictures of similar emotional content (either low-arousing pleasant, high-arousing pleasant, low-arousing unpleasant, or high-arousing unpleasant) but with varying complexity were randomly presented. This procedure was chosen in order to make it easier for participants to perceive subtle differences in terms of emotional contents and complexity. Participants were allowed to take a short self-timed break between these blocks. They were told that the four blocks would vary in terms of emotional contents. The entire experiment lasted around 75 minutes, after which the participants were debriefed and dismissed.

In order to assess objective measures of complexity using data compression, the original JPEG files were transformed into ZIP files (settings: maximum compression rate, compression mechanism deflate, dictionary size 32 kB, word size 64 and 2 CBU-threads) and 7z files (settings: maximum compression rate, compression mechanism LZMA, dictionary size 32 MB, word size 64 and 2 CBU-threads) using the 7-Zip file manager. The original JPEG files were also transformed into lossless GIF files using Adobe Photoshop CS5 (settings: palette local selective, colors 256, forced black-white colors, no transparency, dither diffusion 75%, exact colors and normal order of lines). Two other common lossless compression formats were included, namely PNG and TIFF. The TIFF files were compressed by using the Lempel-Ziv-Welch algorithm. All new file types were further compressed to ZIP and 7z files as described above, leading to twelve different compression file types in total. The file sizes of the pictures of the respective compression formats were assessed in bytes as given by Command Prompt.

Two conventional edge detection parameters were determined, namely perimeter detection and the Canny edge detection. Image analyses were conducted using the Image Processing Toolbox in Matlab (The MathWorks, Inc., Natick, Massachusetts, USA). In order to perform perimeter detection, grayscale images were transformed into binary images by considering the global image threshold using Otsu’s method [128]. The perimeter function returns a binary image showing the perimeter pixels of objects, indicating the changes of intensity at the edges of an image. To be considered as a perimeter pixel, a pixel must have the value one (i.e., being white) in the binary image and be connected to at least one zero-valued pixel (i.e., being black). The default connectivity of four was used for the analysis. Four measures of perimeter detection were determined: the file size of the JPEG, PNG and TIFF compressions of the perimeter images conducted using the standard settings in Matlab and a raw measure based on the sum of the white pixels representing the edges of an image after applying the perimeter function. The Canny edge detection method [129] is a widely used tool to detect weak edges appearing in combination with strong edges in grayscale images. The sensitivity threshold was automatically determined as well as the size of the Gaussian filter. JPEG, PNG and TIFF compression files were created from the images after the application of the Canny-algorithm, and a raw measure was determined by calculating the sum of the white pixels of an image.

Cavalcante et al. [104] showed that the RMS contrast of luminance values provide an alternative method for calculating edges in an image. A contrast map of a grayscale image reveals edges based on luminance contrasts, which proved to be a useful predictor of subjectively experienced complexity of a set of day- and nightscapes. We thus calculated the RMS contrast map of each IAPS picture in Matlab as an alternative way of detecting edges. RMS contrast does not depend on the spatial frequency content or the spatial distribution of contrast in the image and is defined as the standard deviation of the pixel intensities [130]. Around every pixel of an input image *I* a neighborhood of 15 x 15 pixels was considered in the calculation of the RMS contrast map **C** as

$$C(i,j)=\sqrt{1/MN\sum \begin{array}{c} N-1\\ i=0 \end{array}\sum \begin{array}{c} M-1\\ j=0 \end{array}(Iij-\bar{I})²}$$

where intensities *I*_*ij*_ are the *i*-th *j*-th element of the two dimensional image of size *M* by *N*. *Ī* is the average intensity of all pixel values in the image. The image *I* is assumed to have pixel intensities normalized in the range [0, 1]. Following Cavalcante et al. [104], we calculated three measures of objective complexity based on the RMS contrast map: the mean of the RMS contrast values, the standard deviation of the mean RMS contrast values, and a measure α, which is a product of the mean and the standard deviation of the RMS contrast values.

Kovesi [123] developed another type of feature detection algorithm based on phase congruency. Whereas measures such as Canny edge detection are sensitive to variations in image illumination and blurring, phase congruency can be considered as an illumination and contrast invariant measure of feature significance. We calculated the maximum moment of phase congruency covariance (M), an indicator of edge strength, for each picture using the phasecong3.m function provided by Kovesi in Matlab (http://www.csse.uwa.edu.au/~pk/research/matlabfns/#phasecong). Similar to the RMS contrast measures, we computed three measures of objective complexity based on edge detection by phase congruency: the mean of the M values, the standard deviation of the mean M values, and a measure β, which constitutes the product of the mean and the standard deviation of the M values.

We also calculated the entropy of the image intensity histogram of a grayscale image [124], another potentially useful measure of objective complexity, which is included in the image processing toolbox of Matlab. Entropy refers to a statistical measure of randomness of an image: If all of the pixels have the same intensity value, the entropy of the image is zero. In other words, the higher the entropy value of an image, the larger its variation in intensity values.

#### Statistical Analysis

Statistical analyses were conducted in IBM SPSS Statistics version 19 (SPSS Inc., Chicago, IL, USA) or Matlab R2010b (The MathWorks, Inc., Natick, Massachusetts, USA). In order to address the issue of controlling for type 1 error in multiple testing of several correlations from the same matrix, we reported adjusted *p*-values calculated by following the sequential Bonferroni-Holm procedure [131]. For regression analyses, it was ensured that all assumptions (no multicollinearity between the predictors; independence, homoscedasticity and normality of the errors) were met for the variables in question. Mediation regression analyses were computed using the SPSS macro “MEDCURVE” [132]. All statistical tests were two-tailed at an alpha level of .05 if not otherwise indicated. This information refers to all four experiments of this study.

### Results and Discussion

Subjective ratings for each picture were averaged across participants prior to any further analysis. In order to ensure that participants rated each stimulus in a consistent fashion, inter-rater reliability was assessed by computing the average measure intra-class correlation coefficient (ICC) using a two-factor random effects model and type consistency [133,134]. We observed a very high inter-rater reliability for all scales when both males and females were considered in the analysis, as evidenced by the following results: familiarity (ICC(2, k) = .93, 95% confidence interval (CI) [.94, .97]), complexity (ICC(2, k) = .94, 95% CI [.92, .96]), pleasantness (ICC(2, k) = .98, 95% CI [.98, .99]) and arousal (ICC(2, k) = .93, 95% CI [.91, .95]). The same type of analysis was also conducted for males and females separately, which revealed the following results for males: familiarity (ICC(2, k) = .92, 95% CI [.89, .94]), complexity (ICC(2, k) = .91, 95% CI [.87, .93]), pleasantness (ICC(2, k) = .96, 95% CI [.95, .97]) and arousal (ICC(2, k) = .88, 95% CI [.84, .91]). Similar results were obtained for the group of females: familiarity (ICC(2, k) = .92, 95% CI [.89, .94]), complexity (ICC(2, k) = .87, 95% CI [.83, .91]), pleasantness (ICC(2, k) = .97, 95% CI [.96, .98]) and arousal (ICC(2, k) = .87, 95% CI [.83, .91]).

An exploratory data analysis revealed that each of the variables referring to the different compression formats and other measures of objective complexity (edge detection, RMS contrast, entropy) contained several outliers that were 2 *SD* above or below the mean. A series of Shapiro-Wilk normality tests indicated that five variables of the measures of compressed file size, edge detection, RMS contrast and entropy deviated significantly from a normal distribution (all *p*s < .05), after removing the outliers. Furthermore, subjective ratings of familiarity, complexity, pleasantness and arousal were generally not normally distributed when all participants were considered together (all *p*s < .08), and when males (all *p*s < .020) and females (all *p*s < .23) were considered separately. Thus, it was decided to employ non-parametric analyses to investigate correlations between this set of variables in a first step. All relationships between the variables were visually inspected in order to ensure that the distributions of the stimuli followed a linear or monotonic function. Since further regression analyses were planned, values 2 *SD* above and below the mean were removed in all variables prior to these correlational analyses.

**Figure 1:**
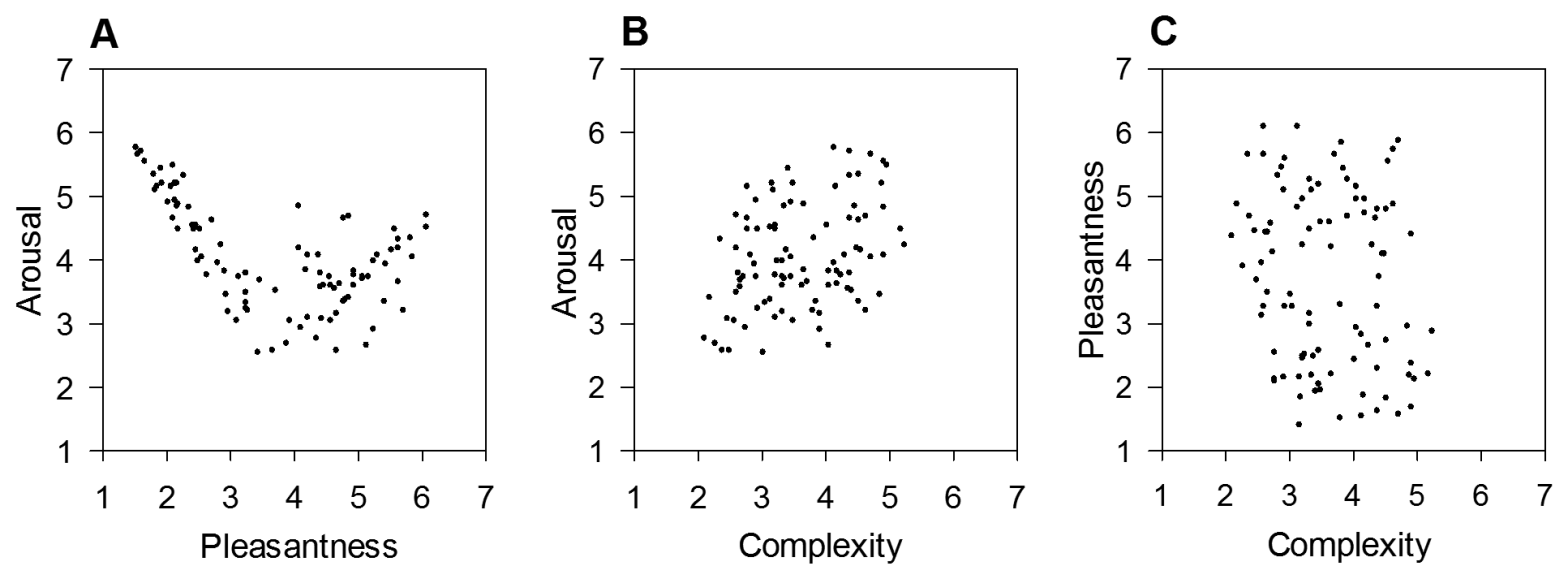
Relationships between pleasantness, arousal and complexity in a set of IAPS pictures. Low numbers refer to low ratings of pleasantness, arousal and complexity, respectively. A) Distribution of IAPS pictures in the pleasantness-arousal space based on mean ratings per picture. B) Relationship between mean complexity and arousal ratings. C) Relationship between mean complexity and pleasantness ratings.

Note that we decided to present three types of relationships graphically: (a) the arousal-pleasantness relationship was considered as important because it shows the distribution of stimuli in the two-dimensional emotion space, which offers insights into the representativeness of the stimulus set, and (b) the arousal-complexity and (c) pleasantness-complexity relationships were considered as most relevant for the discussion of Berlyne’s theory [9] in the context of emotional stimuli. In Figure 1a, the distribution of the 96 IAPS pictures in the arousal-pleasantness space is depicted. Arousal and pleasantness ratings followed a quadratic relationship, *R* ^2^ = .69, *F*(2,91) = 99.19, *p* < .001, y = -2.96x + .35x^2^ + 9.54. The pictures varied to a larger degree in terms of felt pleasantness (min. *M* = 1.39, max. *M* = 6.08, range = 4.69), nearly covering the complete seven-point scale, than in arousal (min. *M* = 2.53, max. *M* = 5.75, range = 3.22). Pleasant high-arousing pictures did not receive average ratings above five on the seven-point rating scale, which stood in contrast to a subset of 14 unpleasant high-arousing pictures. This finding of a smaller number of less arousing pleasant pictures can be explained by the fact that IAPS pictures with erotic scenes, generally inducing high pleasantness and arousal [122], were excluded in the pre-selection process of the current experiment. Another explanation refers to the often reported negativity bias in emotional processing [135], a bias in humans and animals to give greater weight to negative entities such as events and objects. In general, the current distribution of IAPS pictures for a long presentation time of 25 s resembles the distribution for a presentation time of 6 s found for the complete IAPS picture set [122]. Thus, the current stimuli can be considered as representative for this type of stimulus set.

Spearman’s rank-order correlations were calculated between the four subjective ratings (familiarity, complexity, pleasantness and arousal) for all participants as well as for males and females separately (Table 1 and see also Figure S1) because gender differences in response to IAPS pictures have been reported earlier [19]. Familiarity correlated significantly with all other subjective measures when all participants were considered in the analyses. A significant negative correlation between familiarity and complexity was observed (*r*_s_ = -.31), indicating that more familiar pictures were rated as less complex. This finding is in line with earlier reports of effects of familiarity on subjective complexity ratings (for a review, see 102). Similarly, familiarity was moderately negatively associated with arousal (*r*_s_ = -.44): Unfamiliar pictures were experienced as more arousing than familiar ones. A further analysis of the difference between two Spearman rank correlation coefficients based on the Fisher *r*-to-*z* transformation [136] revealed that the correlation between familiarity and arousal was marginally stronger in males compared to females, *z* = 1.91, *p* = .056, but no other gender effects were observed. The positive relationship between familiarity and pleasantness was the strongest (*r*_s_ = .64), showing that familiar semantic contents of pictures was associated with a higher degree of pleasantness.

**Table 1:** Spearman’s rank-order correlations between ratings of familiarity, complexity, pleasantness and arousal of IAPS pictures (N_All_ ≥ 94, n_males_ ≥ 92, n_females_ ≥ 92).

**Measure**		**Familiarity**	**Complexity**
Complexity	f	-.30*	
	m	-.34*	
	All	-.31*	
Pleasantness	F	.66*	-.19
	M	.61*	-.17
	All	.64*	-.18
Arousal	F	-.27*	.30*
	M	-.51*	.37*
	All	-.44*	.36*

Note. *p < .05 after Bonferroni-Holm correction; f = females; m = males, All = all participants; the *dfs* are not the same for all correlations due to slightly different numbers of outliers.

Two further associations in relation to complexity need to be noted. First, complexity correlated moderately positively with arousal (*r*_s_ = .36), in other words, more complex pictures induced higher degrees of arousal (Figure 1b). Next, we did not observe a significant association between complexity and pleasantness in the current data set (*r*_s_ = -.18), although a negative association between these two variables was visually present (Figure 1c). Environmental scenes of low complexity received higher pleasantness ratings. Furthermore, non-parametric partial Spearman’s rank-order correlations were conducted to control for effects of familiarity in the associations between complexity, pleasantness and arousal (Table S1). The relationship between complexity and pleasantness was considerably weakened (*r*_s_ = .01), whereas the positive relationship between complexity and arousal (*r*_s_ = .27) was still significant. The observed positive association between complexity and arousal is in line with Berlyne’s theory [9,21]. However, we observed neither an inverted U-relationship between complexity and pleasantness, nor did we find support for a positive association between complexity and pleasantness as suggested by Nadal et al. [16] for non-artistic representational pictures. On the contrary, we found weak indications for a negative association between these variables when familiarity was not controlled for. This divergence may be due to several reasons: First, hedonic value was defined as beauty in Nadal et al. [16], whereas pleasantness was selected as a measure of hedonic value in the current study. Second, Nadal et al. [16] explicitly avoided the use of affective stimuli, while the current study selected stimuli of different affective contents. Third, Nadal et al. [16] did not explicitly control for familiarity effects when investigating the relationship between complexity and beauty, for example by obtaining subjective ratings for each picture.

In order to gain a deeper understanding of the underlying mechanism of the complexity-pleasantness relationship, a bootstrapping approach [132] was used to examine the existence of an indirect effect of complexity (independent variable X) on pleasantness (dependent variable Y) through arousal (mediator variable M), which would lend support to Berlyne’s collative-motivation model [9]. Due to the nonlinear relation between arousal and pleasantness we conducted multiple regression analyses to compute the instantaneous indirect effect (ϴ_x_) by using the macro “MEDCURVE” for SPSS [132]. According to Hayes and Preacher [132] the instantaneous indirect effect “quantifies how much Y is changing at the point X = x indirectly through X’s affect [sic] on M which, in turn, affects Y” (p. 631). It is important to note that modern mediation theory does not assume a significant correlation between X and Y [137]. All relationships were modeled as linear except for the one between arousal and pleasantness, for which a quadratic relationship was chosen. The results of these regression analyses, conducted for all participants, as well as for males and females separately, are shown in Table 2. In a first step, we calculated a mediator model and specified familiarity as a covariate. This model significantly explained around 26% of the variance of arousal (M) (*p* < .001) when both groups of participants were considered in the analysis. As expected, and in line with Berlyne’s theory, complexity was positively correlated with arousal (*a* = .26, *p* = .008). The dependent mediator model was calculated in a second step and explained 55% of the variance in pleasantness (*p* < .001). There was no significant direct effect of complexity on pleasantness (*c*’ = .10, *p* = .458), which represents how much a unit change in complexity affects pleasantness independent of its effect on arousal. With respect to the effect of arousal on pleasantness, the coefficient of the linear term was positive and significant (*b*_1_ = 2.95, *p* = .009), whereas the coefficient of the quadratic term was negative and significant (*b*_2_ = -.43, *p* = .002). Following the suggestion of Hayes and Preacher [132], the sample mean and one standard deviation above and below the mean were used to estimate the conditional indirect effect, that is, the significance of the indirect effect from complexity to pleasantness through arousal, conditional on specific values of complexity. A confidence interval was computed applying a bias-corrected resampling bootstrap technique with 5000 resamples. These values were negative at a 95% confidence level and thus statistically different from zero, demonstrating a linear instantaneous indirect effect: At all levels of picture complexity, an increase of complexity led to a decrease in pleasantness through the effect of complexity on arousal. A similar pattern of results was observed for males and females and when models without considering familiarity as a covariate were tested. Bearing in mind that causal path models should be treated with caution, especially in cross-sectional designs, an alternative reversed mediation model using pleasantness as the independent variable and complexity as the dependent variable was calculated. This model could only explain 17% of the variance of complexity (*p* = .001), which makes the pleasantness-arousal-complexity causal system a less likely candidate. Taken together, the current data suggests that arousal plays a significant role in the complexity-pleasantness relationship, supporting Berlyne’s theory [7,10]. Moreover, complexity and pleasantness were linearly related when complexity was manipulated by the number of elements in an environmental scene, which is generally in line with the findings by Nadal et al. [16], although the current linear relationship was negative and not positive.

**Table 2:** Indirect effect of complexity (X) on pleasantness (Y) through arousal (M) calculated for IAPS pictures (N_All_ = 94, n_males_ = 92, n_females_ = 92), modeled with a quadratic relation between M and Y and familiarity as a covariate.

		**Model predicting arousal (M)**				
		***Coeff***		***SE***		
Constant	f	3.64***		.58		
	m	4.58***		.50		
	All	4.27***		.53		
Familiarity	f	-.11		.08		
	m	-.39***		.07		
	All	-.29***		.07		
Complexity (X)	f	.31**		.11		
	m	.20*		.09		
	All	.26***		.10		
Summary of model predicting M	f	*R* ^2^ = .13***				
	m	*R* ^2^ = .35***				
	All	*R* ^2^ = .26***				
		**Model predicting pleasantness (Y)**				
Constant	f	-5.10*		2.52		
	m	-1.51		2.03		
	All	-3.57		2.20		
Familiarity	f	.73***		.10		
	m	.36**		.11		
	All	.57***		.11		
Complexity (X)	f	.10		.15		
	m	.12		.12		
	All	.10		.12		
Arousal (M)	f	3.14*		1.23		
	m	2.56*		1.06		
	All	2.95**		1.11		
Arousal squared (M*M)	f	-.42**		.14		
	m	-.43**		.14		
	All	-.43**		.14		
Summary of model predicting Y	f	*R* ^2^ = .53***				
	m	*R* ^2^ = .52***				
	All	*R* ^2^ = .55***				
		***M*_x_**	**ϴ_x_**	**CI 95%**		
	Pictures of low complexity	f	2.77	-.09	-.22	-.01
	m	2.77	-.09	-.22	-.02	
	All	2.80	-.09	-.22	-.02	
Pictures of moderate complexity	f	3.56	-.15	-.33	-.04	
	m	3.62	-.12	-.28	-.03	
	All	3.61	-.13	-.30	-.04	
Pictures of high complexity	f	4.35	-.21	-.50	-.05	
	m	4.48	-.15	-.36	-.03	
	All	4.42	-.18	-.42	-.04	

Note. X = independent variable; M = mediator variable, Y = dependent variable; *Coeff* = unstandardized coefficient; f = females; m = males; ϴ_x_ = instantaneous indirect effect of X on Y through M at a specific value X = x; CI = confidence interval; * *p* < .05, ** *p* < .01, *** *p* < .001.

In order to investigate whether subjective ratings of complexity were associated with objective complexity as measured by compressed file size, a series of Spearman’s rank-order correlations was conducted. Table 3 shows that all four measures of compressed file size were positively associated with subjective complexity. The correlations between the subjective ratings and TIFF file size (*r*_s_ = .53) and JPEG file size (*r*_s_ = .52) were the strongest, followed by the one of PNG file size (*r*_s_ = .46), whereas the one with the GIF file size was the lowest (*r*_s_ = .29) among the different types of compression formats. In general, the correlations between the ZIP and 7Z versions of the respective compression formats and subjective complexity yielded very similar results and are thus not reported here. One exception concerns the ZIP version of the JPEG format, which did not correlate significantly with subjective complexity. There were no significant correlations between the different compression formats and any other type of subjective rating, and further, no indications of any significant gender differences were found. In summary, the current results suggest that the correlation between subjective complexity and the original JPEG file size is among the strongest and that further transformations may not yield better results.

**Table 3:** Spearman’s rank-order correlations between a set of 13 measures of objective complexity applied to IAPS pictures (N = 96) and ratings of familiarity, complexity, pleasantness and arousal.

**Measure**		**JPEG**	**GIF**	**PNG**	**TIFF**	**PERI-RAW**	**CANNY-RAW**	**ENTROPY**	**RMS-CONTR *M***	**RMS-CONTR*SD***	**RMS-CONTR*α***	**PHASE-CONG *M***	**PHASE-CONG *SD***	**PHASE-CONG β**
Familiarity	f	.03	.08	.06	.08	.18	.15	.13	.08	.07	.07	.03	.00	.03
	m	.02	.08	.03	.04	.11	.11	.14	.06	.05	.05	.002	-.05	-.01
	All	.03	.07	.04	.05	.16	.14	.12	.08	.07	.07	.04	-.01	.03
Complexity	f	.48*	.28	.44*	.51*	.41*	.27	.30	.54*	.38*	.53*	.32*	.30*	.33*
	m	.52*	.30	.43*	.49*	.44*	.27	.26	.58*	.45*	.57*	.31*	.34*	.34*
	All	.52*	.29	.46*	.53*	.45*	.29	.28	.59*	.43*	.57*	.34*	.34*	.36*
Pleasantness	f	.08	.09	.08	.11	.07	.14	-.002	.07	.18	.14	.07	.11	.08
	m	.10	.08	.10	.12	.10	.15	.004	.10	.21	.16	.06	.11	.07
	All	.09	.09	.09	.11	.09	.15	.005	.08	.20	.16	.07	.11	.08
Arousal	f	.06	.04	.02	.03	.06	.03	.16	.11	-.03	.05	-.07	-.12	-.06
	m	.13	.05	.12	.13	.05	-.05	.14	.20	.04	.12	-.01	-.01	.02
	All	.12	.06	.11	.11	.08	-.01	.14	.18	.02	.10	-.03	-.04	.02

Note. * *p* < .05 after Bonferroni-Holm correction; f = females; m = males; All = all participants; PERI = perimeter detection; RMS = root mean square; CONTR = contrast; CONG = congruency; all *dfs* ≥ 84 for females; all *dfs* ≥ 83 for males; all *dfs* ≥ 84 for all participants; the *dfs* are not the same for all correlations due to slightly different numbers of outliers.

The observed positive correlations between compressed file size and subjective complexity are in line with earlier findings [101,102,104]. Considering the type of pictures used in the current experiment, i.e. environmental scenes, a comparison with the studies by Cavalcante et al. [104] and Forsythe et al. [74] seems to be appropriate. Cavalcante et al. [104] investigated the relationship between JPEG file size and subjective complexity for 74 days- and nightscapes of cities. Their findings indicated a slightly weaker positive relationship (*r* = .36) as the one observed in the current experiment (*r*_s_ = .52). Forsythe and colleagues [74] examined the relationship between two types of compression file size (JPEG and GIF) with subjective complexity ratings in response to natural pictures (*N* = 200) presented for 5 s. In their study, JPEG file size correlated somewhat more strongly with subjective complexity (*r*_s_ = .60) than in the current experiment. Forsythe et al. [74] also reported a similar correlation between GIF file size and natural pictures (*r*_s_ = .55), which could not be replicated with the current set of stimuli (*r*_s_ = .29).

Table 3 shows the results of Spearman’s rank-order correlations between different types of edge detection measures and subjective ratings of familiarity, complexity, pleasantness and arousal (see Table S2 for inter-correlations between a representative set of objective complexity measures). Only the raw measures of the perimeter detection measure and Canny algorithm are depicted in Table 3 because the measures based on compressed file size yielded very similar results. The four measures of perimeter detection correlated stronger (all *r*_s_ ~ .44) with subjective complexity compared to the measures based on the Canny algorithm (all *r*_s_ ~ .30). Therefore, the current results support the findings by Forsythe et al. [74], who also reported a moderate correlation between perimeter detection and subjective complexity (*r*_s_ = .54) for a set of environmental scene photographs.

It further needs to be noted that we did not observe any significant positive correlations between participants’ self-reported familiarity with the pictures and the four measures of perimeter detection. This result is in line with Forsythe et al. [102], who reported that perimeter detection and its relation to subjective complexity remain unaffected by the self-reported familiarity with the content of a picture. Furthermore, none of the compression file sizes and edge detection measures of complexity correlated significantly with pleasantness, which does not support the findings by Forsythe et al. [74], who reported an association between GIF compression and beauty ratings of various types of visual stimuli.

Finally, we found several positive associations between subjective complexity and objective measures of complexity, which either have not been explored yet or have only rarely been used before. The entropy of the grayscale pictures correlated weakly with subjective complexity (*r*_s_ = .28), which is comparable to the correlation we detected for the raw measure of the Canny edge detection algorithm. Similar correlations were observed for the three measures of edge detection based on phase congruency (all *r*_s_ ~ .34). Among all applied objective complexity measures related to edge detection, subjective complexity correlated best with the mean contrast values of the RMS contrast map (*r*_s_ = .59) and the α-measure of the RMS contrast values (*r*_s_ = .57). These results clearly replicate those of Cavalcante et al. [104], who reported correlations of *r* = ~ .60 between the three RMS contrast measures and subjective complexity ratings of streetscape images, revealing the potential future use of RMS contrast measures as reliable indicators of subjective complexity. It is worth noting that we also found weak, non-significant, indications for a positive association between pleasantness and the standard deviation of the mean RMS contrast values (all *r*_s_ ~ .20). Nevertheless, the current data suggests that correlations between objective measures of complexity and subjective ratings other than complexity are weak, and consequently, that the applicability of objectives measures of complexity to the prediction of subjective pleasantness ratings appears limited for photographs of environmental scenes.

Linear multiple regression analyses were conducted to further investigate the inter-relationships between subjective ratings and objective measures of complexity. Table 4 summarizes the results of a stepwise linear regression model with subjective complexity as the criterion (dependent variable) and a set of ten variables as predictors, including familiarity, pleasantness and arousal as well as a set of seven measures of objective complexity. Familiarity was entered in the first step. Due to issues of multicollinearity, the following six measures of objective complexity were excluded: TIFF and PNG file size, *SD* and α-measures of RMS contrast, and *SD* and β-measures of edge detection based on phase congruency. This regression model allowed addressing the question of whether objective measures can predict subjective complexity better than, for example, subjective arousal. The adjusted *R* ^2^ value indicated that the model predicted 51% of the variance, with the mean values of the RMS contrast measure as the strongest predictor (β = .55, *p* < .001), followed by familiarity (β = -.48, *p* = .001). The RMS contrast measure accounted for around 30% of the variation in subjective complexity. A similar model considering only objective measures of complexity as predictors (Table S3) revealed that the mean of the RMS contrast values was the only significant predictor of subjective complexity (β = .54, *p* < .001), explaining approximately 28% of the variance. Similar regression analyses conducted for males and females separately revealed analogous results except that arousal was a third significant predictor in the model for females (β = .23, *p* = .012). Taken together, these results support the view that familiarity is a significant predictor of subjective complexity that cannot be ignored in any complex models, and further, that the mean RMS contrast values are the strongest predictor of subjective complexity among the current set of objective complexity measures applied to affective environmental scenes.

**Table 4:** Summary of linear stepwise regression analysis for ten variables predicting subjective complexity ratings of IAPS pictures (N = 70).

**Variable**	***B***	***SEB***	**Β**
Step 1			
Constant	5.15	.35	
Familiarity	-.35	.08	-.47***
Adjusted *R* ^*2*^	.21		
*F*	18.98***		
Step 2			
Constant	4.02	.33	
RMS contrast *M*	20.66	3.17	.55***
Familiarity	-.37	.06	-.48***
Adjusted *R* ^*2*^	.51		
*F*	36.58***		
*ΔR* ^*2*^	.30		

Note. * *p* < .05, ** *p* < .01, *** *p* < .001; *B* = unstandardized regression coefficient; SE = standard error; β = standardized regression coefficient; *ΔR* ^2^ *=* difference in the proportion of variance explained.

## Experiment 2

### Methods

#### Participants

Forty German-speaking students (20 males, 24.4 ± 4.1 years; age range 19-38 years; 20 females, 22.0 ± 1.5 years, age range 20-26 years) participated in the study in return for course credit. Most of these students were enrolled in a psychology degree and none of them was pursuing an art history degree or was an arts expert. Participants did not take part in any other experiment in the current study, and they all had normal or corrected-to-normal visual acuity.

#### Materials

Ninety-six high-quality digital reproductions of colored oil and acryl paintings were downloaded from two digital image libraries (*prometheus*, http://prometheus-bildarchiv.de, and *ARTStor*, http://www.artstor.org/index.shtml). These representational paintings comprised different styles from the end of the 18^th^ to the beginning of the 20^th^ century, including renowned artists such as Achenbach, Cezanne, Courbet, Friedrich, Gauguin, Gericault, Goya, Manet, Monet, Turner, and Van Gogh (see Supporting Information Stimulus List S2). The semantic content of the paintings was similar to the one of the IAPS pictures used in Experiment 1, depicting human beings in different situations of everyday life (excluding erotic scenes), landscapes, animals and plants. Paintings that were highly familiar and for which the semantic content could not be easily understood, as judged by the co-authors, were not included in the stimulus set. As in Experiment 1, the paintings were pre-selected, as judged by the co-authors, to fall into one of the four quadrants spanned by arousal and pleasantness. For this purpose, the range of the semantic content was matched as much as possible with the one of environmental scenes. In order to achieve sufficient variation in subjective arousal, paintings with dull colors where preferably pre-selected for the low-arousing emotion quadrants. Half of the paintings in each hedonic category (*n* = 24) depicted a figure-ground composition and half a complex scene. The paintings were all in landscape format and saved in the same size (1024 x 768 pixels) and resolution (72dpi) in JPEG format (maximum quality) as the IAPS pictures used in Experiment 1. Due to the natural preponderance of figure-ground compositions in portrait format rather than in landscape format, especially when paintings contained human faces, several paintings were cut to landscape format using Adobe Photoshop CS5 software. Other modifications concerned the presence of signatures or frames, which were removed. It was made sure that all modifications yielded natural representations of paintings without any deformations. Since we aimed to preserve the emotional contents of the paintings, we decided not to control for variations of brightness within the stimulus set. The paintings were not modified in any other way.

In order to rule out that any observed differences between the results regarding paintings and IAPS pictures were due to differences in brightness, an arithmetic mean model was applied to calculate the average brightness of the 96 paintings and 96 IAPS pictures in the RGB (red-green-blue) color space in Matlab. Specifically, brightness can be thought of as the arithmetic mean μ of the red, green and blue color coordinates (μ = (R+G+B)/3). The sets of paintings (*M* = .38, *SD* = .14) and IAPS pictures (*M* = .37, *SD* = .14) did not significantly differ in their average RGB pixel levels, *t*(190) = -.35, *p* = .726.

Two self-developed questionnaires were used, one asking specific questions referring to the experiment and one probing general interest in arts and expertise in visual arts. The former questionnaire comprised two questions about whether participants recognized the styles of the paintings and could name the century of their creation. They were also asked to indicate the degree of their general liking for the paintings on a seven-point Likert scale (from “not at all” to “very much”). Moreover, participants reported the general level of difficulty associated with giving ratings of complexity on a seven-point Likert scale (from “very easy” to “very difficult”). Finally, participants estimated the percentage of paintings they had seen at least once before the experiment (ranging from 0% to 100%). The self-developed questionnaire on interest in arts and expertise in visual arts comprised three parts. A set of eleven questions was used to examine participants’ interest in visual arts (nine-point Likert scale, from “fully disagree” to “fully agree”), followed by a part in which participants had to indicate whether they are familiar with the names of a range of artists as well as to name their nationality and their associated style by free verbal responses. In the final part, participants were confronted with representations of six paintings and again had to indicate their familiarity with the paintings and to name the artist and the style.

#### Procedure

In order to be able to compare results across the two different visual stimuli sets used in this study, the procedure of Experiment 2 was generally similar to the one in Experiment 1, with the exception that the instructions for familiarity ratings were as follows: “Please rate your familiarity with the painting.” In addition, after participants had finished the experiment, they filled a self-developed one-page questionnaire relating directly to the experiment followed by a four-page questionnaire assessing general interest in arts and expertise in visual arts. These additional questionnaires extended the length of the experimental session to 90 minutes. As in Experiment 1, the original paintings in JPEG format were transformed into a set of twelve different compression formats of which the file sizes were determined. Several edge detection measures and the entropy were calculated by using the same procedures as described in Experiment 1.

### Results and Discussion

With regard to the questionnaire data on participants’ liking for the paintings used in the experiment and the level of difficulty of giving complexity judgments, all except one out of four Shapiro-Wilk normality tests revealed that the data was not normally distributed (*p*s < .01). Further analyses showed that males (*n* = 20) and females (*n* = 20) did not differ in their general liking for the paintings, Mann-Whitney *U* = 181.5, *p* = .608, *r* = -.08, and also not in their self-reported degree of difficulty in judging the complexity of the paintings, Mann-Whitney *U* = 155.5, *p* = .214, *r* = -.20. Note that *r* refers to an effect size estimate that is derived by converting test statistics into *z*-scores and dividing by the square root of the number of total observations, *r* = *z*/√*N* ( [138], p. 19). The standard values of *abs*(r) for small, medium and large effect sizes are as follows: small: *r* = .10; medium: *r* = .30; large: *r* = .50.

Participants reported to have seen approximately 5-10% of the paintings prior to the experiment on average. Regarding the results of the questionnaire on the self-reported interest in visual arts, we did not observe a significant difference between males (*n* = 19, *M* = 6.24, *SD* = 1.5) and females (*n* = 20, *M* = 5.4, *SD* = 1.34), *t*(37) = 1.85, *p* = .072, *r* = .29. One male participant did not respond to all the items of the art interest questionnaire. Screening of the other parts of the questionnaire (art expertise) indicated that none of the participants was an art expert.

As in Experiment 1, subjective ratings were averaged across participants for each painting prior to the main analysis. In order to test whether participants rated each stimulus in a consistent fashion, inter-rater reliability was assessed by computing the average measure intra-class correlation coefficient (ICC) using a two-factor random effects model and type consistency [133,134]. We observed high inter-rater reliability for all four rating scales, as evidenced by the following results based on all participants: familiarity (ICC(2, k) = .86, 95% CI [.82, .90]), complexity (ICC(2, k) = .93, 95% CI [.90, .96]), pleasantness (ICC(2, k) = .96, 95% CI [.95, .97]) and arousal (ICC(2, k) = .90, 95% CI [.87, .92]). The following results were obtained for females: familiarity (ICC(2, k) = .82, 95% CI [.76, .87]), complexity (ICC(2, k) = .87, 95% CI [.83, .90]), pleasantness (ICC(2, k) = .94, 95% CI [.92, .95]) and arousal (ICC(2, k) = .84, 95% CI [.79, .88]). In the group of males, inter-rater reliability for the four scales was as follows: familiarity (ICC(2, k) = .71, 95% CI [.61, .79]), complexity (ICC(2, k) = .89, 95% CI [.86, .92]), pleasantness (ICC(2, k) = .92, 95% CI [.90, .95]) and arousal (ICC(2, k) = .80, 95% CI [.73, .85]).

Data screening of the variables of objective complexity for outliers revealed between zero and eleven outliers per variable. Outliers were defined by values ± 2 *SD* from the mean and removed. A series of Shapiro-Wilk normality tests indicated that four variables deviated significantly from normality after removal of outliers (all *p*s < .03). The majority of the variables of self-reported familiarity, complexity, pleasantness and arousal did not deviate significantly from a normal distribution when all participants were considered together (all ps > .04), or when males (all ps > .09) and females (all ps > .01) were considered separately.

**Figure 2:**
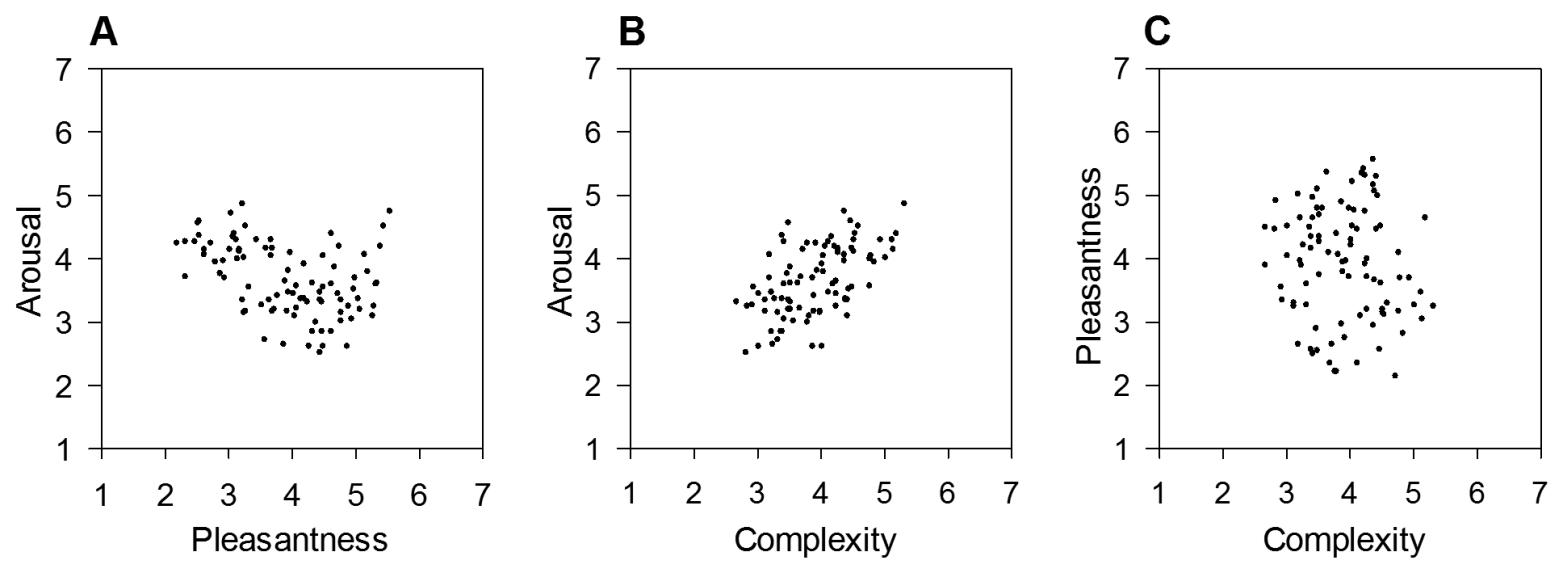
Relationships between pleasantness, arousal and complexity in a set of representational paintings. Low numbers refer to low ratings of pleasantness, arousal and complexity, respectively. A) Distribution of paintings in the pleasantness-arousal space based on mean ratings per picture. B) Relationship between mean complexity and arousal ratings. C) Relationship between mean complexity and pleasantness ratings.

The distribution of the paintings in the two-dimensional emotion space (Figure 2a) was very similar to the one of IAPS pictures observed in Experiment 1 (Figure 1a), showing a quadratic relationship between arousal and pleasantness, *R* ^2^ = .30, *F*(2,88) = 18.88, *p* < .001, y = -2.42x + .28x^2^ + 8.69. However, the strength of the relationship was weaker in the set of paintings than the one observed for IAPS pictures. A further comparison between the two emotion spaces indicated that the range of arousal for paintings (min. *M* = 2.50, max. *M* = 4.85, range = 2.35) was smaller than the one seen for IAPS pictures (min. *M* = 2.53, max. *M* = 5.75, range = 3.22). The range of pleasantness associated with the paintings (min. *M* = 2.13, max. *M* = 5.55, range = 3.42) was also smaller than the one associated with IAPS pictures (min. *M* = 1.39, max. *M* = 6.08, range = 4.69). Fewer paintings were regarded as highly unpleasant and high-arousing. Nevertheless, the current data suggest the presence of a negativity bias [135] of emotional processing of representational paintings. This finding may be explained by the fact that the environmental scenes used in Experiment 1 had similar semantic contents as the current representational paintings and that both types of stimuli presumably induced emotional processes relevant to survival in nature.

Table 5 and Figure 2 show that, when males and females were considered together, the nature of the relationships between the subjective ratings of familiarity, complexity, pleasantness and arousal was generally similar to the one observed in Experiment 1. However, in contrast to Experiment 1, familiarity was not significantly associated with complexity (*r*_s_ = -.07), but the data revealed a significant negative association with arousal (*r*_s_ = -.28) and a significant positive association with pleasantness (*r*_s_ = .78). The latter correlations were of a roughly similar magnitude as the ones observed in the set of IAPS pictures. Moreover, complexity was significantly positively related with arousal (*r*_s_ = .54). The non-significant negative relationship between complexity and pleasantness was weaker (*r*_s_ = -.10) than in Experiment 1 (*r*_s_ = -.18). In order to control for effects of familiarity, partial Spearman’s rank-order correlations (Table S4) were performed on the subjective reports of complexity, pleasantness and arousal. A comparison between results presented in Tables 5 and S4 revealed that the positive association between complexity and arousal gained slightly in strength (*r*_s_ = .59), whereas the relationship between complexity and pleasantness did not change.

**Table 5:** Spearman’s rank-order correlations between ratings of familiarity, complexity, pleasantness and arousal in response to representational paintings (N_All_ ≥ 89, n_males_ ≥ 89, n_females_ ≥ 89).

**Measure**		**Familiarity**	**Complexity**
Complexity	f	-.33*	
	m	.16	
	All	-.07	
Pleasantness	f	.81*	-.26*
	m	.66*	.10
	All	.78*	-.10
Arousal	f	-.45*	.56*
	m	-.10	.43*
	All	-.28*	.54*

Note. * *p* < .05 after Bonferroni-Holm correction; f = females; m = males; All = all participants; the *dfs* are not the same for all correlations due to slightly different numbers of outliers.

In light of Berlyne’s theory [9], the finding of a positive association between complexity and arousal could be corroborated in both genders (*r*_s_ = .59), as was the case for IAPS pictures in Experiment 1 (*r*_s_ = .27). This association between complexity and arousal was significantly stronger for paintings (*z* = -2.63, *p* = .009) when effects of familiarity were partialed out. Furthermore, we did not observe a clear linear relationship between complexity and pleasantness when males and females were analyzed separately (Figure S2) and when familiarity effects were controlled for, which is in line with results of Experiment 1. A significant negative linear relationship was only present in the group of females when familiarity was not considered in the analysis. This finding does not lend support to Nadal et al.’s [16] theory of a positive linear relation between complexity and hedonic value if complexity is manipulated by the number and variety of elements. One may hypothesize that this discrepancy in research findings could be related to the fact that different hedonic measures were applied in the two studies (beauty vs. pleasantness). Future studies may thus investigate whether the three relationships between different dimensions of complexity and beauty (linear, U-shaped and inverted U-shaped) described by Nadal et al. [16] hold true for other types of hedonic measures besides beauty. Another alternative explanation for the current finding may simply be attributed to the different degree of aesthetic quality and motivational relevance of the visual materials under investigation compared to those used by Nadal et al. [16], who avoided including stimuli inducing strong affect.

The current data also revealed several gender differences with regard to subjective ratings (Figure S2), which were not present in Experiment 1. In general, familiarity correlated stronger with complexity, pleasantness and arousal in females compared to males. The relationship between familiarity and complexity was reversed in the two groups, *z* = -3.31, *p* < .001. In females, the relationship between familiarity and complexity was negative (*r*_s_ = -.33), while it was positive in males (*r*_s_ = .16). Familiarity and pleasantness were also more strongly associated in females (*r*_s_ = .81) than in males (*r*_s_ = .66), *z* = 2.19, *p* = .029. Moreover, the negative relationship between familiarity and arousal was stronger in females (*r*_s_ = -.45) than in males (*r*_s_ = -.10), *z* = -2.52, *p* = .012. Last, the direction and strength of the relationship between complexity and pleasantness (Figure S2) differed when males (*r*_s_ = .10) and females (*r*_s_ = -.26) were analyzed separately, *z* = -2.4, *p* = .016. The current findings thus corroborate earlier reports of gender differences in visual art appreciation [86,139,140], highlighting the necessity to consider gender in the study of empirical aesthetics.

As in Experiment 1, a mediation model was calculated using the macro “MEDCURVE” in SPSS [132] in order to examine whether arousal mediates the relationship between complexity and pleasantness. The relationships between complexity and arousal as well as between complexity and pleasantness were modeled as linear, while the relationship between arousal and pleasantness was modeled as quadratic. Results of these analyses, separately for males, females and all participants, are presented in Table 6. In a first step, we calculated a mediator model and specified familiarity as a covariate. This model significantly explained 44% of the variance in arousal (M) (*p* < .001) when both groups of participants were considered in the analysis. As expected, and in line with Berlyne’s theory and results of Experiment 1, complexity was positively correlated with arousal (*a* = .49, *p* < .001). In a second step, the dependent variable model was calculated and explained 59% of the variance in pleasantness (*p* < .001). The direct effect of complexity on pleasantness was not significant (*c*’ = .11, *p* = .346), which we also observed in Experiment 1. With respect to the effect of arousal on pleasantness, the coefficient of the linear term and the coefficient of the quadratic term were not significant. Only the effect of familiarity was highly significant (*p* < .001). There were no indications of any significant instantaneous indirect effects when males and females were analyzed together, which stands in clear contrast to results reported in Experiment 1. However, a further mediation model conducted without considering familiarity as a covariate revealed significant linear indirect effects of complexity through arousal on pleasantness based on data of all participants. As in Experiment 1, we tested an alternative mediation model (pleasantness-arousal-complexity) which could only explain around 36% of the variance, implying that the complexity-arousal-pleasantness path model may better capture the underlying perceptual and cognitive processes.

**Table 6:** Indirect effect of complexity (X) on pleasantness (Y) through arousal (M) calculated for representational paintings (N_All_ = 86, n_males_ = 87, n_females_ = 88), modeled with a quadratic relation between M and Y and familiarity as a covariate.

		**Model predicting arousal (M)**				
		*Coeff*		*SE*		
Constant	f	2.88***		.54		
	m	2.74***		.38		
	All	2.80***		.42		
Familiarity	f	-.34**		.10		
	m	-.34**		.10		
	All	-.38***		.10		
Complexity (X)	f	.48***		.09		
	m	.44***		.07		
	All	.49***		.07		
Summary of model predicting M	f	*R* ^2^ = .40***				
	m	*R* ^2^ = .33***				
	All	*R* ^2^ = .44***				
		**Model predicting pleasantness (Y)**				
Constant	f	-2.75		2.03		
	m	-.33		2.36		
	All	-1.20		2.38		
Familiarity	f	1.24***		.12		
	m	.87***		.13		
	All	1.21***		.14		
Complexity (X)	f	.22		.12		
	m	.15		.11		
	All	.11		.12		
Arousal (M)	f	1.59		1.02		
	m	1.15		1.38		
	All	1.03		1.28		
Arousal squared (M*M)	f	-.26*		.13		
	m	-.22		.20		
	All	-.19		.18		
Summary of model predicting Y	f	*R* ^2^ = .69***				
	m	*R* ^2^ = .51***				
	All	*R* ^2^ = .59***				
		***M*_x_**	**ϴ_x_**	**CI 95%**		
	Pictures of low complexity	f	3.47	-.13	-.30	.03
	m	3.13	-.11	-.25	.05	
	All	3.30	-.10	-.28	.08	
Pictures of moderate complexity	f	4.08	-.20	-.37	-.08	
	m	3.80	-.17	-.32	-.05	
	All	3.93	-.16	-.34	.02	
Pictures of high complexity	f	4.69	-.28	-.49	-.12	
	m	4.47	-.23	-.46	-.06	
	All	4.55	-.22	-.50	.05	

Note. X = independent variable; M = mediator variable; Y = dependent variable; *Coeff* = unstandardized coefficient; f = females; m = males; All = all participants; ϴ_x_ = instantaneous indirect effect of X on Y through M at a specific value X = x; CI = confidence interval; * *p* < .05, ** *p* < .01, *** *p* < .001.

By using a bootstrapping approach, instantaneous indirect effects of complexity on pleasantness through arousal were observed when males and females were analyzed separately. Interestingly, this mediation effect was only present for pictures of moderate and high complexity in both groups, suggesting that for pictures of moderate and high complexity an increase in complexity led to a decrease in pleasantness through an increase in arousal. For pictures of low complexity, no such mediation effect was found. Further, it needs to be noted that the direct effect of complexity on pleasantness was nearly significant in females (*c*’ = .22, *p* = .074). We believe that a gender-wise analysis is clearly better suited to detect mediation effects since we have shown that, for example, correlations between familiarity and the respective subjective ratings differed between males and females in the current experiment. Important information would have been lost if only results based on the pooled data had been presented.

A critical interpretation of the current results suggests that, on the one hand, the underlying mechanisms of the perception of visual artistic stimuli may differ from the perception of environmental scenes. For example, results of Experiment 1 provide a very coherent picture with regard to significant indirect effects of complexity through arousal on pleasantness as well as with regard to the absence of a direct effect of complexity on pleasantness. In the current experiment involving paintings, significant indirect effects only emerged when males and females were analyzed separately, and further, weak indications of a direct effect were observed in females. Moreover, the relationship between complexity and arousal was stronger in the perception of representational paintings compared to the perception of environmental scenes. On the other hand, albeit the noted differences between the results of Experiments 1 and 2, which are presumably partly due to gender effects with regard to familiarity ratings of paintings, the presence of significant indirect effects when viewing environmental scenes and representational paintings lends support to Berlyne’s theory [7,10]. Future studies may investigate the presence of a mediation effect with different types of visual stimuli and hedonic measures to see whether the current findings can be generalized.

The relationships between subjective and objective complexity as measured by compressed file size were investigated by Spearman’s rank-order correlations. Table 7 shows that none of these correlations was significant (i.e., JPEG, GIF, PNG and TIFF formats as well as their 7z and ZIP versions which are not reported here), which stands in clear contrast to the results reported in Experiment 1. Thus, these findings are not in line with Forsythe et al. [74], who reported that JPEG file size (*r*_s_ = .40) and GIF file size (*r*_s_ = .47) correlated significantly with subjective complexity judgments of representational paintings (*N* = 148). Furthermore, the current twelve compression file sizes neither correlated with familiarity, pleasantness nor arousal, which is in agreement with the results in relation to IAPS pictures investigated in Experiment 1 (Table 3). The lack of a significant relationship between compressed file size and subjective complexity could be due to the fact that the current set of paintings was pre-selected to vary considerably in the number of elements present in a visual scene. A figure-ground composition, such as a portrait, may be judged as relatively simple but even a relatively uniform background may be difficult to compress because of the natural variability of the brush marks present in most of these paintings. Consequently, the application of compression file size as a measure of objective complexity may depend on the specific type of complexity dimension under investigation as well as on the stimulus type. Forsythe et al. [74] did not pre-select their stimuli according to one or more complexity dimensions (M. Nadal-Roberts, personal communication, October 15, 2012), thus making it difficult to establish a direct comparison with their results.

Table 7 also presents the Spearman’s rank-order correlations between the four subjective variables and the set of edge detection measures as well as entropy. Neither the raw nor the compressed file size measures of perimeter detection yielded any significant relationships with subjective complexity, which can partly be explained by the nature of the paintings under investigation. For example, perimeter detection may be sensitive to coarse features of the background in a figure-ground composition and thus weaken the positive association with subjective complexity observed in Experiment 1. Such an interpretation of the results would explain why the current findings differ from those reported by Forsythe et al. [74], who found a positive association between perimeter detection and subjective complexity (*r*_s_ = .37). Bearing in mind that Forsythe et al. [74] did not specifically include figure-ground compositions, it may be that perimeter detection was still successful in predicting subjective complexity to a moderate extent.

The measures related to the Canny algorithm correlated negatively (but not significantly) with subjective complexity (*r*_s_ = -.23), a result which clearly differs from the positive association between these measures as presented in Experiment 1 (*r*_s_ ~ .29). A closer inspection of the paintings after analysis by the Canny algorithm revealed that fine features of the background, due to the individual brush strokes, were indeed largely detected, which may explain the negative association with subjective complexity. Moreover, there was no significant association between the entropy of a grayscale image and subjective complexity. Interestingly, the mean RMS contrast values correlated only weakly with subjective complexity, but positive correlations were observed for the standard deviation of the RMS contrast values (*r*_s_ = .24) and the RMS contrast α-measure (*r*_s_ = .25). However, the degree of these correlations was considerably smaller than the one found for IAPS pictures in Experiment 1 (*r*_s_ ~ .60). The standard deviation of the mean values of edge detection based on phase congruency yielded the strongest (and only significant) correlation (*r*_s_ ~ .38) among all measures. It seems that objective measures of complexity including a measure of dispersion, such as the *SD* measures of RMS contrast and edge detection based on phase congruency as well as the related α- and β-measures, worked better for the current set of artworks than for environmental scenes. In summary, our findings indicate that objective measures which were successful in predicting subjective complexity of environmental scenes were much less efficient for representational paintings. Inter-correlations between a representative set of objective measures of complexity can be found in the Supporting Information (Table S5).

The present results not only revealed significant correlations between measures of subjective and objective complexity, but also indications of relationships between objective measures of complexity and other types of subjective ratings. The correlation between the standard deviation RMS contrast measure and arousal was significant (*r*_s_ = .37), which stands in clear contrast to the findings of Experiment 1. In addition, a negative relationship between Canny edge detection measures and subjectively reported arousal (all *r*_s_ ~ -.23) was observed. The finding that edge detection measures correlated with arousal in paintings but not in IAPS pictures may be explicable by the fact that the correlation between subjective arousal and complexity was stronger in paintings (*r*_s_ = .54) than in IAPS pictures (*r*_s_ = .36). Furthermore, the data showed indications of weak correlations between subjective pleasantness and measures of Canny edge detection (*r*_s_ =.26) and the standard deviation of the RMS contrast values (*r*_s_ = -.28), respectively. This lends some support to the finding by Forsythe et al. [74], who reported a significant correlation between objective complexity as measured by compressed file size and subjective ratings of beauty. Last but not least, indications of a positive correlation between familiarity and JPEG, PNG and TIFF file sizes were observed in males, indicating that objective measures of complexity are not completely unaffected by familiarity as previously reported by Forsythe et al. [102]. In summary, the pattern of results observed for the current set of representational paintings differs in several respects from the results in relation to IAPS pictures (Experiment 1), drawing attention to the specificity of digital reproductions of representational art and environmental scenes within a common framework of complexity.

A stepwise regression analysis was conducted in order to examine whether objective measures of complexity are better predictors of complexity than, for example, subjective arousal. As in Experiment 1, we included seven measures of objective complexity (JPEG and GIF file size, perimeter detection, Canny algorithm, entropy, *SD* measures of RMS contrast and phase congruency) as well as arousal and pleasantness in the as predictors in the stepwise regression analysis. Familiarity was entered in a first step, although it was not significantly correlated with subjective complexity when all participants were considered together in the analysis. This procedure was chosen in order to guarantee comparability across the four experiments. The results, presented in Table 8, yielded a significant model (adjusted *R* ^2^ = .40) based on two significant predictors, namely arousal (β = .56, *p* < .001) and the standard deviation of the phase congruency edge detection measure (β = .28, *p* = .013). A comparison with results presented in Experiment 1 showed two main differences: First, subjective arousal was a better predictor of complexity ratings of representational paintings than the best objective measure, which was not the case for the set of IAPS pictures. Second, the mean RMS contrast values were the strongest predictor in Experiment 1, whereas the *SD* measure of edge detection based on phase congruency yielded the best results in the current experiment.

**Table 7:** Spearman’s rank-order correlations between a set of 13 measures of objective complexity applied to representational paintings (N = 96) and ratings of familiarity, complexity, pleasantness and arousal.

**Measure**		**JPEG**	**GIF**	**PNG**	**TIFF**	**PERI-RAW**	**CANNY-RAW**	**ENTROPY**	**RMS-CONTR *M***	**RMS-CONTR *SD***	**RMS-CONTR α**	**PHASE-CONG *M***	**PHASE-CONG *SD***	**PHASE-CONG β**
Familiarity	f	.05	-.07	.07	.08	.02	.20	.10	-.07	-.16	-.14	-.08	-.06	-.05
	m	.30	.05	.18	.24	-.03	.01	.10	.05	.04	.04	-.03	.04	.01
	All	.22	.05	.21	.23	.06	.13	.12	.05	-.03	.001	-.05	-.01	-.02
Complexity	f	-.02	.05	-.09	-.08	.11	-.28	.03	.09	.25	.23	.27	.37*	.30
	m	.02	.01	-.04	.002	.14	-.22	.09	.09	.20	.22	.24	.37*	.29
	All	.02	.06	-.06	-.02	.15	-.23	.10	.11	.24	.25	.25	.38*	.30
Pleasantness	f	.20	-.07	.11	.18	.03	.30	.03	-.06	-.27	-.17	-.09	-.10	-.09
	m	.16	-.14	.07	.12	-.04	.16	.01	-.13	-.24	-.18	-.05	-.05	-.03
	All	.17	-.09	.10	.15	.03	.26	.05	-.11	-.28	-.19	-.07	-.08	-.06
Arousal	f	-.09	.02	-.13	-.12	.02	-.23	-.02	.12	.34	.23	.23	.21	.26
	m	-.07	.03	-.10	-.11	-.02	-.25	.07	.09	.31	.23	.13	.16	.14
	All	-.10	.05	-.09	-.12	.03	-.23	.04	.14	.37*	.27	.23	.24	.25

Note. *p < .05 after Bonferroni-Holm correction; f = females; m = males; All = all participants; PERI = perimeter detection; RMS = root mean square; CONTR = contrast; CONG = congruency; all *dfs* ≥ 81 for females; all *dfs* ≥ 79 for males; all *dfs* ≥ 80 for all participants; the *dfs* are not the same for all correlations due to slightly different numbers of outliers.

**Table 8:** Summary of linear stepwise regression analysis for ten variables predicting subjective complexity ratings of representational paintings (N = 59).

**Variable**	***B***	***SEB***	**β**
Step 1			
Constant	4.28	.56	
Familiarity	-.14	.20	-.10
Adjusted *R* ^*2*^	-.01		
*F*	.53		
Step 2			
Constant	.69	.79	
Arousal	.75	.14	.63***
Familiarity	.15	.17	.11
Adjusted *R* ^*2*^	.34		
*F*	15.76***		
*ΔR* ^*2*^	.35		
Step 3			
Constant	-.32	.85	
Arousal	.67	.13	.56***
*SD* phase congruency	22.37	8.74	.28*
Familiarity	.22	.16	.15
Adjusted *R* ^*2*^	.40		
*F*	13.74***		
*ΔR* ^*2*^	.07		

Note. * *p* < .05, **p < .01, ***p < .001; *B* = unstandardized regression coefficient; SE = standard error; β = standardized regression coefficient; *ΔR* ^2^ *=* difference in the proportion of variance explained.

Similar stepwise regression analyses were conducted for both genders separately. In general, the results were comparable to those described above, with arousal and the *SD* measure of phase congruency as significant predictors of subjective complexity. However, the *SD* RMS contrast measure was identified as a significant suppressor variable in both multiple regressions. Following Pandey and Elliott [141], the current type of suppression could be identified as negative suppression, meaning that the *SD* RMS contrast measure was positively correlated with other predictors and the outcome variable but had a negative beta weight when entered into the regression model. For females, the model yielded an adjusted *R* ^*2*^ of .41, *F*(4,57) = 11.42, *p* <.001, with the following predictors: familiarity (β = -.06, *p* = .61), arousal (β = .51, *p* < .001), *SD* phase congruency (β = .39, *p* = .001) and *SD* RMS contrast (β = -.25, *p* = .038). The final model based on the data of males also explained 41% of the variance in subjective complexity, *F*(4,57) = 11.44, *p* <.001, including the following predictors: familiarity (β = .26, *p* = .013), arousal (β = .53, *p* < .001), *SD* phase congruency (β = .43, *p* = .001) and *SD* RMS contrast (β = -.26, *p* = .035).

As a final step, a multiple stepwise regression model only including objective measures of complexity was employed to predict subjective complexity ratings averaged over all participants. The results (Table S6) suggest that the *SD* measure of phase congruency (β = .69, *p* <.001) was the strongest predictor, followed by GIF compression file size (β = .39, *p* = .003) and the SD measure of RMS contrast (β = -.30, *p* = .039). Only 25% of the variance in subjective complexity could be explained, *F*(3,60) = 7.90, *p* <.001. Again, the *SD* measure of RMS contrast was identified as a negative suppressor variable. A comparison between the current results and those presented in Experiment 1 suggest that subjective complexity in response to IAPS pictures was easier to predict by means of objective measures because the model based on one predictor (mean RMS contrast values) was as successful as the current model based on three predictors. In both experiments, measures based on RMS contrast values contributed as significant predictors of visual complexity, highlighting their future use in studies involving objective complexity measures.

## Experiment 3

### Methods

#### Participants

Thirty-six German-speaking psychology students (18 males, 23.6 ± 2.8 years, age range 20-30 years; 18 females, 22.2 ± 3.9 years, age range 19-33 years) participated in the experiment. Participants received course credit in return for their participation and did not take part in any other experiment in this study. All participants were enculturated in Western tonal music from their birth onwards and non-musicians, i.e., they were neither playing a musical instrument at the time of the experiment nor had they played an instrument for longer than four years in their childhood or youth. Sixteen participants had played an instrument in their past (musical training = 1.10 ± 1.40 years). All participants reported normal hearing.

#### Materials

The ninety-two musical excerpts in WAV format were taken from the stimulus set described in Marin et al. [83]. The stimuli can be considered as representative of the 19^th^ century Romantic piano solo music repertoire and comprised composers such as Brahms, Chopin, Grieg, Mendelssohn, Schubert and Schumann (see Supporting Information Stimulus List S3). Based on the distribution of the musical excerpts in the two-dimensional emotion space reported in Experiment 1 in Marin et al. [83], 23 stimuli were chosen to exhibit maximal variation in arousal and pleasantness. Excerpts thus included the outer representatives of each emotion quadrant spanned by arousal and pleasantness to obtain large differences in arousal and/or pleasantness and to cover the whole emotion space of this particular musical style. All stimuli were cut to 25 s of length (keeping the original beginning of the excerpts and removing parts of the ending of the excerpts selected from [83]), and a 500 ms fade in/out was added to the excerpts using Audacity 1.3.14 software. Importantly, in the pre-selection process only stimuli in which the emotional content was not largely changing over the 25 s were selected because the aim was to select stimuli that induce a relatively unambiguous emotion. The sounds were not modified in any other way.

A self-developed two-page questionnaire was used to explore participants’ musical background. Participants answered questions referring to their amount of musical training and indicated how often they listen to a particular musical style (*n* = 14, ranging from classical, pop, Jazz to music of foreign cultures) on a seven-point Likert scale (from “never” to “very often”). In a similar way, they reported how often they listen to music actively (without doing other activities at the same time), passively (while doing other activities at the same time) or in a live-concert setting. For each of these ratings, participants named at least one musical style to which the rating mostly referred. Furthermore, they named their least and most preferred musical style and reported on the role music plays in their daily lives (seven-point Likert scale, with “no role… a big role” as anchors). Three questions referred directly to the experiment. Participants had to indicate whether they recognized the musical style and, if so, they were asked to name it. They also reported the degree of liking for the music on a seven-point Likert scale (from “not at all” to “very much”) and the degree of difficulty of judging the complexity of the music (from “very easy” to “very difficult”).

#### Procedure

In general, the design and procedure of the behavioral experiment were identical to Experiments 1 and 2 of the current study, except that participants were specifically screened for their amount of musical training during the recruitment process and further, that all participants were tested individually in a quiet room. Familiarity ratings referred to the specific musical excerpt and the instructions said: “Please rate your familiarity with the musical excerpt.” Musical excerpts were blocked according to emotional contents as was the case for pictures in Experiments 1 and 2. This procedure was chosen in order to make it easier for participants to perceive subtle differences in terms of emotional contents and complexity. The order of the ratings (familiarity, complexity, pleasantness and arousal) was the same for all participants. A comparison between the inter-relationships of familiarity, arousal and pleasantness with those based on data of Experiment 1 as reported in Marin et al. [83] revealed similar results regarding the direction and strength of the relationships, thus making the presence of order effects unlikely. After the experiment, participants filled the questionnaire. All stimuli were played through an external sound card (E-MU audio interface, 0204/USB) and Sennheiser high-quality (HD 380 pro) headphones. The volume was fixed to approximately 72 dB SPL (a-weighted), which constitutes an averaged dB value measured by a commercial dB-meter (Voltcraft SL-400 decibel meter that was calibrated immediately prior to usage) during the presentation of the second practice trial. This excerpt was one of the loudest trials in the set, and participants were asked whether they were comfortable with this loudness level before the actual experiment started. The experiment lasted around 90 minutes in total.

In order to obtain objective measures of complexity by means of data compression, the original uncompressed audio files in WAV format were transformed into one lossless compression format (Free Lossless Audio Codec, FLAC) and two common lossy compression formats (MP3 and Ogg Vorbis) by using Audacity 1.3.14 software. The 16 bit FLAC files were compressed to the maximum level 8. MP3 files were created with the following settings: bit-rate mode ‒ pre-set extreme, 220-260 kbit/s; variable speed ‒ standard; and channel mode ‒ joint stereo. The quality setting for the transformation into Ogg Vorbis files was set to 8, which referred to a variable bit rate of 256 kbit/s. The file sizes of the audio files of the respective compression formats were assessed in bytes. The event density per second of each musical excerpt was analyzed by means of the MIRtoolbox [125] in Matlab.

### Results and Discussion

Two female participants did not provide answers to two questions in the post-questionnaire, but their behavioral data was included in the analysis. Shapiro-Wilk normality tests showed that the data referring to participants’ musical background, i.e., the role of music in participants’ lives and the frequency of listening to classical music, was not normally distributed (all *p*s < .05). Mann-Whitney-U-tests indicated that males (*n* = 18) and females (*n* = 16) differed neither in their ratings of the role music played in their daily lives, *U* = 108.0, *p* = .195, *r* = -.22, nor in their frequency of listening to classical music, *U* = 108.5, *p* = .212, *r* = -.21. Regarding the questionnaire referring to the actual experiment, an independent *t*-test revealed that males (*n* = 18, *M* = 5.11, *SD* = .96) and females (*n* = 18, *M* = 5.06, *SD* = 1.16) did not significantly differ in their general liking for the piano solo music, *t*(34) = .16, *p* = .877, *r* = .03. With respect to the difficulty of judging the complexity of the musical excerpts, there was also no significant difference between the genders, *t*(34) = -.42, *p* = .678, *r* = .07 (males: *M* = 4.33, *SD* = 1.28; females: *M* = 4.50, *SD* = 1.10). Taken together, these results suggest that males and females had a similar musical background, especially with regard to classical music, and that both participant groups reported comparable levels of liking for the music played in the experiment and of the difficulty of judging complexity thereof. Further inspection of the musical background questionnaire indicated that only three participants had a high interest in classical music.

The main analysis was based on ratings averaged across participants for each musical excerpt. Inter-rater reliability was assessed by computing the average measure intra-class correlation coefficient (ICC) using a two-factor random effects model and type consistency [133,134]. A high degree of reliability was found for the four rating scales, as shown by the following results: familiarity (ICC(2, k) = .80, 95% CI [.74, .86]), complexity (ICC(2, k) = .96, 95% CI [.94, .97]), pleasantness (ICC(2, k) = .88, 95% CI [.84, .91]) and arousal (ICC(2, k) = .93, 95% CI [.91, .95]). ICCs computed for both genders separately revealed the following results for males: familiarity (ICC(2, k) = .62, 95% CI [.49, .72]), complexity (ICC(2, k) = .90, 95% CI [.87, .93]), pleasantness (ICC(2, k) = .73, 95% CI [.64, .80]) and arousal (ICC(2, k) = .88, 95% CI [.84, .91]). In the group of females, ICCs were generally somewhat higher than those observed for males: familiarity (ICC(2, k) = .70, 95% CI [.60, .78]), complexity (ICC(2, k) = .92, 95% CI [.89, .94]), pleasantness (ICC(2, k) = .83, 95% CI [.78, .88]) and arousal (ICC(2, k) = .86, 95% CI [.81, .90]).

After removal of several outliers prior to the analysis, a series of Shapiro-Wilk normality tests showed that most of the data of the four subjective ratings (familiarity, complexity, pleasantness and arousal) deviated significantly from a normal distribution when all participants were considered together (all *p*s < .122), and when males (all *p*s < .104) and females (all *p*s < .459) were considered separately. Shapiro-Wilk normality tests also revealed significant deviations from normality for the MP3 (*p* = .001) and the FLAC (*p* = .002) compression file sizes as well as for the measure of event density (*p* = .039) although several outliers were removed. It was thus decided to run non-parametric tests in order to investigate the relationships between these sets of variables.

**Figure 3:**
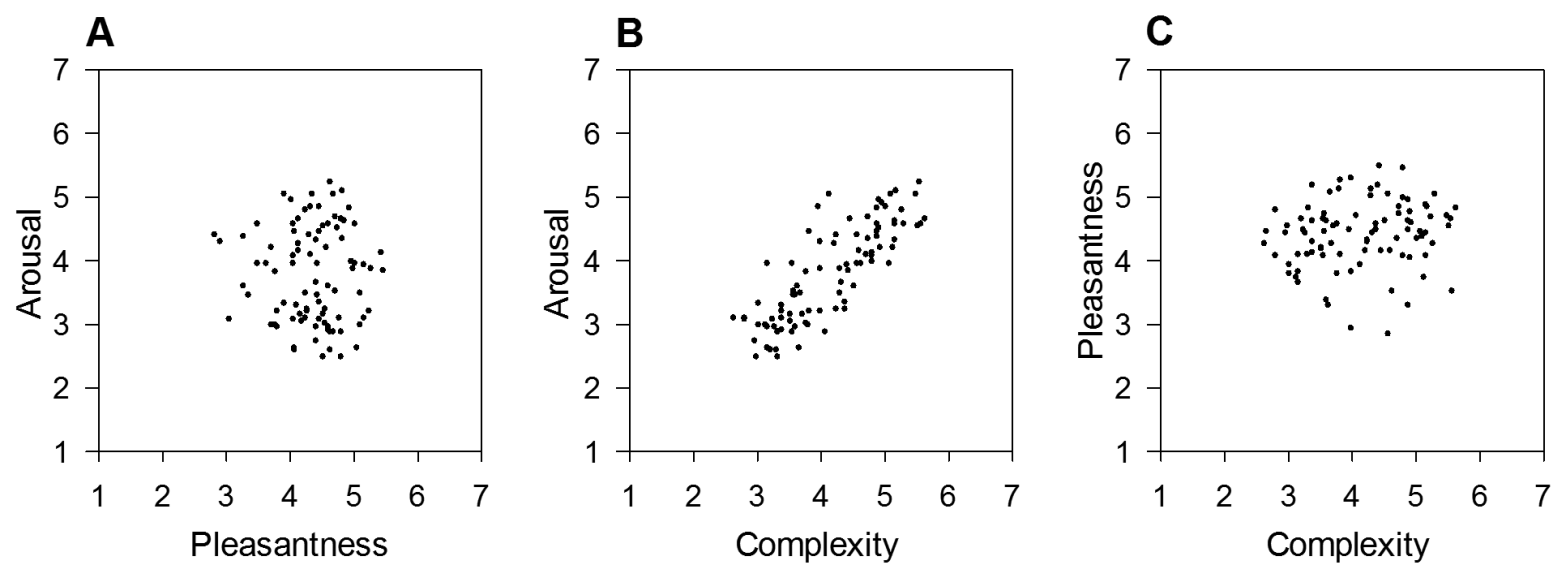
Relationships between pleasantness, arousal and complexity in a set of piano solo music excerpts. Low numbers refer to low ratings of pleasantness, arousal and complexity, respectively. A) Distribution of excerpts of Romantic piano solo music in the pleasantness-arousal space. B) Relationship between mean complexity and arousal ratings. C) Relationship between mean complexity and pleasantness ratings.

Figure 3a depicts the distribution of the musical excerpts in the arousal-pleasantness space. These excerpts varied to a similar degree in arousal (min. *M* = 2.47, max. *M* = 5.22, range = 2.75) as in pleasantness (min. *M* = 2.83, max. *M* = 5.47, range = 2.64). A comparison of the current results with the findings of Experiment 1 described in [83] revealed that both distributions cover a similar part of the emotion space based on seven-point scales (pleasantness: ~ 3.0 to 5.5; arousal: 2.0 ~ 5.5). In comparison to the emotion spaces of Experiments 1 and 2 of the present study, no significant quadratic relationship between arousal and pleasantness was found for the current excerpts of piano solo music.

Table 9 shows the relationships between the four subjective measures (familiarity, complexity, pleasantness and arousal) in response to Romantic piano solo music, analyzed for females and males separately (Figure S3) and for both groups together (Figure 3). We observed a strong positive relationship for ratings of familiarity and pleasantness (*r*_s_ = .75), as was the case for the respective variables in the visual domain (see Experiments 1 and 2). Familiarity did not significantly correlate with subjective complexity judgments of music (*r*_s_ = .03), which stands in contrast to the negative relationship observed for IAPS pictures, but at the same time, is in line with the finding for representational paintings (*r*_s_ = -.07), suggesting that familiarity and complexity may be less related in artistic stimuli if effects of gender are ignored. In contrast to the findings reported in Experiments 1 and 2, familiarity did not significantly negatively correlate with arousal (*r*_s_ = -.09). This correlation was significantly different compared to the one observed with IAPS pictures, *z* = -2.53, *p* = .011.

**Table 9:** Spearman’s rank-order correlations between ratings of familiarity, complexity, pleasantness and arousal of piano solo music excerpts (N_All_ ≥ 88, n_males_ ≥ 87, n_females_ ≥ 85).

**Measure**		**Familiarity**	**Complexity**	**Pleasantness**
Complexity	f	.11		
	m	-.21		
	All	.03		
Pleasantness	f	.64*	.07	
	m	.54*	.25	
	All	.75*	.18	
Arousal	f	-.13	.80*	-.15
	m	-.13	.83*	.18
	All	-.09	.83*	-.03

Note. *p < .05 after Bonferroni-Holm correction; f = females; m = males; All = all participants; the *dfs* are not the same for all correlations due to slightly different numbers of outliers.

Partial Spearman’s rank-order correlations between complexity, pleasantness and arousal were conducted to eliminate effects of familiarity (Table S7). Familiarity effects became particularly obvious in the group of males. The positive association between complexity and pleasantness gained in strength (*r*_s_ = .42), which was also the case for the relationship between arousal and pleasantness (*r*_s_ = .28). As expected, the relationship between complexity and arousal did not change when familiarity was controlled for (*r*_s_ = .82). Importantly, the underlying basis of the positive association between complexity and pleasantness in males, the only significant association observed in this series of experiments so far, is difficult to interpret. In the current experiment, the degree of complexity was not manipulated but variations in complexity were naturally present in the stimulus set under investigation. Stimuli were selected on the basis of their affective content [83]. The positive relationship between subjective complexity and arousal (controlling for effects of familiarity) was also present in Experiments 1 and 2, but in terms of the strength of this relationship the results of the three experiments clearly differ: The weakest correlation was observed in the set of IAPS pictures (*r*_s_ = .27), followed by the one in paintings (*r*_s_ = .59), and the strongest correlation was found in the current experiment for music (*r*_s_ = .83). The differences between these three correlations were significant (all *p*s < .003). All together, these results support Berlyne’s hypothesis [9] of a positive association between arousal and complexity.

In comparison to Experiment 1, we found several indications of gender effects in response to representational art and music. For instance, complexity correlated positively with pleasantness in males (*r*_s_ = .42) in response to music, indicating that more complex music induced higher levels of pleasantness. This relationship was negative in females (Figure S3), leading to a non-significant association when both genders were considered together (Figure 3). While the correlation between subjective complexity and pleasantness was negative in both genders in response to IAPS pictures, a positive association as proposed by Nadal et al. [16] between these variables was only present in males in response to representational paintings and music. Furthermore, the relationship between familiarity and complexity differed in direction and magnitude with regard to representational art (negative in females, positive in males). This pattern of results was not present in the current experiment, in which a (non-significant) negative relationship was detected in males and a positive relationship in females. Consequently, the present findings support the view that gender affects particularly the relationships between familiarity, pleasantness and complexity in response to artistic stimuli, whereas the relationship between arousal and complexity appears to be less affected.

As a next step, we performed a mediation analysis in order to examine whether the indirect effect of complexity on pleasantness through arousal can also be detected in the musical domain. A bootstrapping approach was used to test the mediator model [132]. First, a mediator model (arousal) was computed. A dependent variable model (pleasantness) was computed in a second step. In both models, familiarity was specified as a covariate. All relationships between these variables were modeled as linear. The model predicting the mediator was significant and explained 69% of the variance in arousal (Table 10). Complexity had a strong significant positive impact on arousal (*a* = .79, *p* < .001). The dependent variable model was also significant and explained 55% of the variance in pleasantness. The results showed a significant direct effect of complexity on pleasantness after controlling for arousal (*c*’ = .25, *p* = .005), which was not present in Experiments 1 and 2. The bootstrapped estimate of the indirect effect was negative and significant at the 95% confidence level, suggesting that pleasantness decreases linearly as complexity increases. Mediation models calculated for males and females separately revealed that this indirect effect was only significant in females.

**Table 10:** Indirect effect of complexity (X) on pleasantness (Y) through arousal (M) with familiarity as a covariate, calculated for piano solo music (N_All_ = 86, n_males_ = 84, n_females_ = 83).

		**Model predicting arousal (M)**			
		***Coeff***	***SE***		
Constant	f	1.63***	.39		
	m	-.37	.60		
	All	1.19*	.46		
Familiarity	f	-.25*	.11		
	m	.05	.14		
	All	-.22	.13		
Complexity (X)	f	.69***	.06		
	m	.95***	.07		
	All	.79***	.06		
Summary of model predicting M	f	*R* ^2^ = .65***			
	m	*R* ^2^ = .70***			
	All	*R* ^2^ = .69***			
		**Model predicting pleasantness (Y)**			
Constant	f	2.57***	.38		
	m	1.00	.48		
	All	1.23**	.39		
Familiarity	f	.73***	.11		
	m	.79***	.11		
	All	.94***	.10		
Complexity (X)	f	.23**	.08		
	m	.25*	.10		
	All	.25**	.09		
Arousal (M)	f	-.34**	.10		
	m	-.04	.09		
	All	-.21*	.09		
Summary of model predicting Y	f	*R* ^2^ = .48***			
	m	*R* ^2^ = .40***			
	All	*R* ^2^ = .55***			
		**Indirect effect**	**CI 95%**		
		f	-.23	-.37	-.10
		m	-.04	-.17	.11
		All	-.17	-.32	-.03

Note. X = independent variable; M = mediator variable; Y = dependent variable; *Coeff* = unstandardized coefficient; f = females; m = males; All = all participants; CI = confidence interval; * *p* < .05, ** *p* < .01, *** *p* < .001.

While the results of Experiments 1 and 2 provided evidence for a complexity-arousal-pleasantness path model, which is essentially in line with the mediating role of arousal proposed by Berlyne [9], the current results also revealed a significant direct effect of complexity on pleasantness after controlling for arousal besides a significant indirect effect. In other words, the relationship between complexity and pleasantness is not completely mediated through arousal. This finding, if replicated with other musical styles, may have important implications for models of aesthetic responses that comprise different sensory domains. The current results further suggest that gender affects the mediating role of arousal in response to music, which we did not observe in the two experiments involving visual stimuli.

In line with our hypothesis, the file sizes of the three types of compression format (MP3, Ogg Vorbis and FLAC), but not those of the original WAV formats, correlated significantly positively with subjective complexity (Table 11). FLAC compression size yielded the highest correlation coefficient (*r*_s_ = .65). Compared to results of Experiments 1 and 2, the current correlations between compressed file size and subjective complexity for musical stimuli were generally stronger. Interestingly, the three types of compressed file sizes correlated even slightly (non-significantly) better with reported arousal (e.g., *r*_s_ = .73 for the FLAC compression file size) than with subjective complexity (e.g., *r*_s_ = .65 for the FLAC compression file size). The results further indicated that the size of MP3 and FLAC files correlated negatively, but less strongly, with familiarity in males (*r*_s_ ~ -.28). We also observed a significant negative correlation between the FLAC file size and pleasantness in females (*r*_s_ = -.31). These latter findings again highlight the need to consider gender in research on musical complexity in the context of empirical aesthetics.

**Table 11:** Spearman’s rank-order correlations between compression algorithms applied to excerpts of piano solo music, their event density and rated familiarity, complexity, pleasantness and arousal (N_All_ ≥ 85, n_males_ ≥ 84, n_females_ ≥ 83).

**Measure**		**MP3**	**Ogg Vorbis**	**FLAC**	**Event Density**
Familiarity	f	-.19	.01	-.18	.23
	m	-.28	-.03	-.28	.11
	All	-.20	.02	-.22	.23
Complexity	f	.55*	.35*	.58*	.54*
	m	.61*	.41*	.70*	.56*
	All	.58*	.38*	.65*	.57*
Pleasantness	f	-.26	-.13	-.31*	.15
	m	.01	.10	-.06	.25
	All	-.17	-.02	-.24	.25
Arousal	f	.61*	.38*	.69*	.37*
	m	.64*	.44*	.74*	.43*
	All	.62*	.42*	.73*	.42*

Note. * *p* < .05 after Bonferroni-Holm correction; f = females; m = males; All = all participants; the *dfs* are not the same for all correlations due to slightly different numbers of outliers.

There was also a significant positive correlation between event density and subjective complexity (*r*_s_ = .57), supporting our hypothesis. This correlation explained about 32% of the variance in subjective complexity and thus had a comparable strength to the ones observed for the correlations between the FLAC and MP3 compressed file sizes and subjective complexity. Event density also correlated positively with arousal (*r*_s_ = .42). The data further revealed a weak positive association between pleasantness and event density in males (*r*_s_ = .25). Taken together, these results are similar to those of Experiment 2, in which we also found indications that subjective ratings of paintings other than complexity weakly correlated with measures of edge detection and compressed file size. Specifically, the positive relationship between edge detection measures and arousal in the set of paintings and the one between compressed file size, event density and arousal in the set of piano solo music may be due to the stronger relationship between subjective complexity and arousal in visual and musical artistic stimuli compared to IAPS pictures. Table S8 reports the inter-relationships between all objective measures of musical complexity.

A linear stepwise regression analysis was conducted to explore whether subjective or objective measures were better predictors of subjective complexity (Table 12). Familiarity was entered in the first block, followed by arousal and pleasantness as well as by the three measures of file size and event density in a second block. The final model (after four steps) was significant with an adjusted *R* ^*2*^ of .75, *F*(4,74) = 59.21, *p* < .001. Arousal was the strongest predictor (β = .70, *p* <.001), followed by pleasantness (β = .28, *p* = .001) and the FLAC file size (β = .20, *p* = .03). It is notable that arousal was the strongest predictor in the model, which was also the case in the model predicting subjective complexity of representational art in Experiment 2, but not in the model predicting subjective complexity ratings of IAPS pictures in Experiment 1. However, this pattern of results changed when males and females were analyzed separately. Familiarity was again entered in a first step. The model based on data from the male group was significant with an adjusted *R* ^*2*^ of .79, *F*(5,66) = 55.41, *p* < .001, revealing the following significant predictors: arousal (β = .60, *p* < .001), event density (β = .24, *p* = .001), MP3 file size (β = .17*p* = .022) and pleasantness (β = .14, *p* = .046). The regression analysis based on data of the female group was similar, indicating a significant model with an adjusted *R* ^*2*^ of .74, *F*(5,66) = 42.03, *p* < .001, and the following predictors: arousal (β = .61, *p* < .001), event density (β = .26, *p* = .001), MP3 file size (β = .19*p* = .002) and pleasantness (β = .18, *p* = .026). The finding that the type and order of the predictors are the same when both genders are analyzed separately and different when a model is fitted to the averaged data is worth noting. One possible explanation may relate to the different directions of the correlations between complexity and familiarity in males and females (Table 9). This result not only shows that familiarity is a crucial variable in the field of empirical aesthetics, but also that gender effects may distort findings that are based on averaged data across both groups. In other words, research designs that balance for gender without applying an in-depth analysis may yield misleading results.

**Table 12:** Summary of linear stepwise regression analysis for seven variables predicting subjective complexity ratings of piano solo music (N = 79).

**Variable**	***B***	***SEB***	**Β**
Step 1			
Constant	4.01	.76	
Familiarity	.04	.24	.02
Adjusted *R* ^*2*^	-.01		
*F*	.03		
Step 2			
Constant	.09	.49	
Arousal	.91	.07	.85***
Familiarity	.21	.13	.10
Adjusted *R* ^*2*^	.71		
*F*	98.11***		
*ΔR* ^*2*^	.72		
Step 3			
Constant	-.43	.51	
Arousal	.91	.06	.85***
Pleasantness	.35	.13	.22**
Familiarity	-.12	.17	-.06
Adjusted *R* ^*2*^	.74		
*F*	73.80***		
*ΔR* ^*2*^	.03		
Step 4			
Constant	-1.37	.66	
Arousal	.75	.10	.70***
Pleasantness	.44	.13	.28**
FLAC file size	8.15E-6	< .001	.20*
Familiarity	-.14	.17	-.07
Adjusted *R* ^*2*^	.75		
*F*	59.21***		
*ΔR* ^*2*^	.02		

Note. * *p* < .05, **p < .01, *** *p* < .001; *B* = unstandardized regression coefficient; SE = standard error; β = standardized regression coefficient; *ΔR* ^2^ *=* difference in the proportion of variance explained.

A stepwise regression model predicting subjective complexity only by means of objective measures of complexity (Table S9) showed that FLAC file size (β = .34, *p* = .001), event density (β = .46, *p* < .001) and MP3 file size (β = .27, *p* < .001) were significant predictors, explaining approximately 60% of the variance in subjective complexity, *F*(3,75) = 43.12, *p* < .001. These results did not differ between males and females. In comparison to recent models of musical complexity based on objective measures [50,115], the current model accounts for a similar proportion of variance by using fewer predictors, which furthermore can be computed more easily.

## Experiment 4

### Methods

#### Participants

Forty German-speaking psychology students (20 males, 24.5 ± 6.0 years, age range 20-46 years; 20 females, 22.2 ± 1.7 years, age range 20-26 years) participated in the experiment in return for course credits. These participants did not take part in any other experiment in this study. All participants were enculturated in Western tonal music from their birth onwards and were non-musicians, i.e., they were neither playing a musical instrument at the time of the experiment nor had they played an instrument for longer than four years in their childhood or youth. Twenty-one participants had played an instrument in their past (musical training = 1.01 ± 1.12 years). All participants reported normal hearing.

#### Materials

In this experiment, the aim was to manipulate musical complexity by the number of musical instruments present in an auditory scene, that is, one instrument (piano solo music) vs. three instruments (piano trios). This approach made a meaningful comparison with Experiments 1 and 2, in which visual complexity was manipulated in a similar vein, possible. Thus, 40 piano trio excerpts (see Supporting Information Stimulus List S4) were pre-selected by the first author, a trained musicologist, from the Romantic music repertoire to fall into one of the four emotion quadrants defined by the dimensions of arousal and pleasantness [18]. The first author had experience in the selection of music excerpts in Experiment 3 which helped her to choose an appropriate range of emotions induced by piano trios. All excerpts were taken from piano trios of representative Romantic composers, such as Schubert, Schumann, Brahms, Spohr and Liszt, and in the standard piano trio instrumentation (piano, violin and violoncello). It was ensured that all three instruments were present in an excerpt and that the emotional content was not changing over time. All stimuli were cut to 25 s of length, and a 500 ms fade in/out was added to the excerpts using Audacity 1.3.14 software. The sounds were not modified in any other way. Based on the results reported in Experiment 3, 20 piano solo music excerpts were chosen as best representatives of each emotion quadrant spanned by arousal and pleasantness (see Supporting Information Stimulus L3). This guaranteed that the complete range of the emotion space of this particular musical style was covered by 40 excerpts. The same questionnaire as in Experiment 3 was used to assess participants’ musical background and to collect data regarding their liking for the music and the experienced level of difficulty of judging complexity.

#### Procedure

In general, the experimental procedure was identical to the one described in Experiment 3, except for that participants were tested in pairs separated by a wall in a quiet room. Each participant was wearing Sennheiser (HD 380 pro) headphones. Furthermore, the piano solo music and piano trio excerpts were blocked according to their position in the arousal-pleasantness space. All trials (10 piano solo music excerpts and 10 piano Trió excerpts) were randomized within the four blocks, which were also randomized across participants. After the experiment, participants completed the questionnaire and were debriefed and dismissed. The experimental session lasted approximately 80 minutes on average. The 40 piano trio excerpts in WAV format were also transformed into MP3, Ogg Vorbis and FLAC files. The file size and event density of each musical excerpt were determined as described in Experiment 3.

### Results and Discussion

A series of Shapiro-Wilk normality tests indicated that the data referring to participants’ musical background, i.e., the role of music in participants’ lives and the frequency of listening to classical music, was largely not normally distributed when the data was considered separately for males and females (all *p*s < .140). Similarly, the distributions of the variables regarding the general liking of the music presented in the experiment and the difficulty of giving complexity ratings were not normally distributed (all *p*s < .219). Mann–Whitney *U*-tests indicated that males (*n* = 20) and females (*n* = 20) differed neither in their ratings of the role music plays in their daily lives, *U* = 200.0, *p* = 1.00, *r* =.00, nor in their frequency of listening to classical music, *U* = 192.5, *p* = .836, *r* = -.03. Moreover, males and females liked the musical stimuli to a similar extent, *U* = 171.5, *p* = .423, *r* = -.13, and they also reported a similar degree of difficulty of judging the complexity of the musical excerpts, *U* = 188.5, *p* = .750, *r* = -.05.

Further Mann–Whitney *U*-tests revealed that participants of Experiments 3 and 4 did not differ with regard to the role music played in their lives, *U* = 594.0, *p* = .329, *r* = -.11, and also not with regard to the frequency of listening to classical music, *U* = 645.0, *p* = .699, *r* = -.04. In a similar vein, the participant groups did not significantly differ in their liking ratings for the musical stimuli, *U* = 654.5, *p* = .480, *r* = -.08, and in their self-reported difficulty of judging musical complexity, *U* = 657.0, *p* = .500, *r* = -.08. These results thus allowed a meaningful comparison between the results of the two experiments involving musical stimuli.

As in Experiments 1-3, the main analysis was based on ratings for each musical excerpt averaged across participants. Inter-rater reliability was assessed by computing the average measure intra-class correlation coefficient (ICC) using a two-factor random effects model and type consistency [133,134]. A high degree of reliability was found for the four rating scales when all participants were considered together, as shown by the following results: familiarity (ICC(2, k) = .73, 95% CI [.63, .81]), complexity (ICC(2, k) = .96, 95% CI [.95, .97]), pleasantness (ICC(2, k) = .79, 95% CI [.72, .85]) and arousal (ICC(2, k) = .93, 95% CI [.90, .95]). Similar results were obtained for the group of males, except for that inter-rater reliability was not particularly high for familiarity ratings: familiarity (ICC(2, k) = .44, 95% CI [.25, .61]), complexity (ICC(2, k) = .92, 95% CI [.89, .94]), pleasantness (ICC(2, k) = .65, 95% CI [.52, .75]) and arousal (ICC(2, k) = .87, 95% CI [.82, .91]). Inter-rater reliability was also considered sufficiently high in females: familiarity (ICC(2, k) = .62, 95% CI [.49, .73]), complexity (ICC(2, k) = .92, 95% CI [.90, .95]), pleasantness (ICC(2, k) = .69, 95% CI [.58, .78]) and arousal (ICC(2, k) = .87, 95% CI [.82, .90]). It is worth noting that although non-musicians participated in the experiment, the inter-rater reliability for complexity ratings was very high, suggesting that untrained listeners are able to perceive differences in musical complexity within and across musical styles of Romantic music in a reliable fashion. We also obtained high inter-rater reliability for complexity ratings of representational art in Experiment 3.

**Figure 4:**
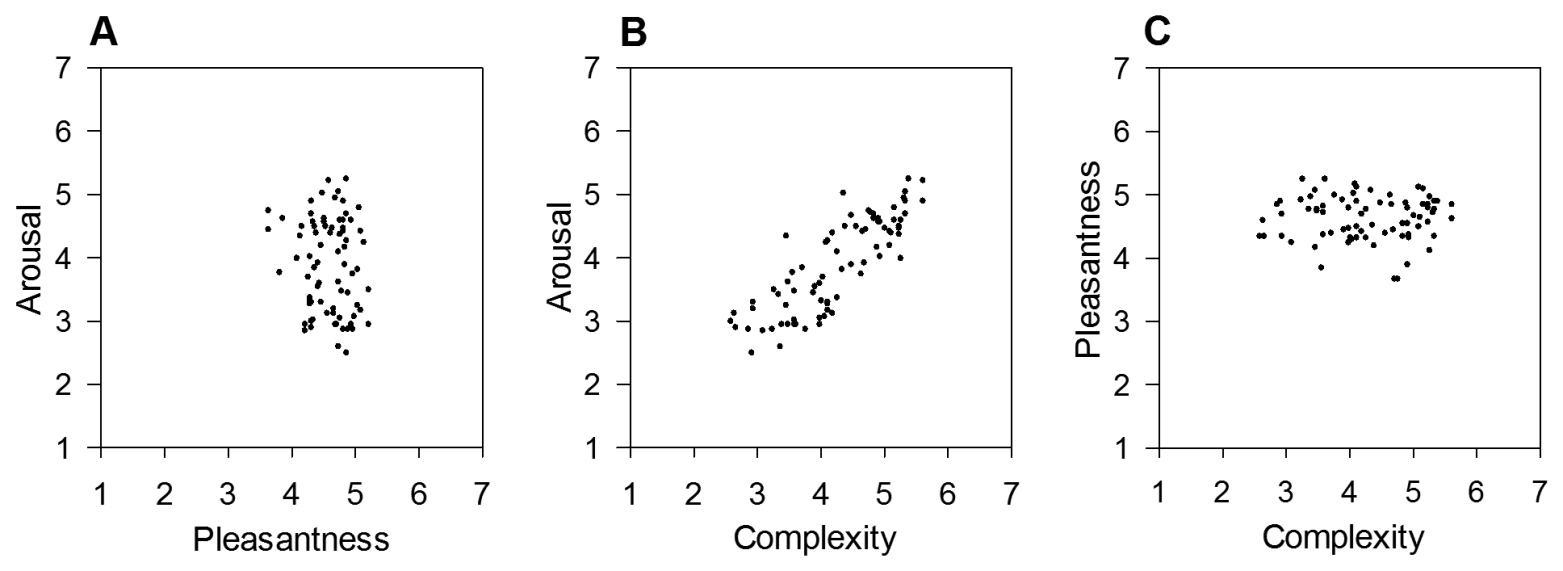
Relationships between pleasantness, arousal and complexity in a set of piano solo and chamber music excerpts. Low numbers refer to low ratings of pleasantness, arousal and complexity, respectively. A) Distribution of Romantic piano solo music and piano trio excerpts in the pleasantness-arousal space. B) Relationship between mean complexity and arousal ratings. C) Relationship between mean complexity and pleasantness ratings.

After the removal of outliers two *SD* above or below the mean, Shapiro-Wilk normality tests showed that most of the subjective ratings with regard to the musical excerpts, with the exception of those of familiarity, were not normally distributed when both genders were considered together (all *p*s < .03), and when males (all *p*s < .10) and females (all *p*s < .15) were analyzed separately. Deviations from normality were also observed for the Ogg Vorbis compressed file size (*p* < .001), therefore it was decided to conduct non-parametric correlation analyses as in Experiments 1-3. A comparison between the distributions of musical excerpts in the arousal-pleasantness space in Experiment 3 (Figure 3a) and 4 (Figure 4a) revealed that excerpts were less spread apart in terms of pleasantness when piano trios and piano solo music were combined in one stimulus set.

Here, complexity was manipulated by mixing piano solo excerpts with piano trios on the grounds that these have a greater number of melodic parts than the piano solo excerpts used in Experiment 3. However, adding additional melodic parts does more than simply add extra complexity to a piece of music. It may also add timbral streaming, harmonic richness and complexity, and greater expressive scope. It thus remains possible that these factors were responsible for any differences found between Experiments 3 and 4, rather than differences in subjective complexity per se. A comparison between the subjective complexity ratings of the original 40 piano solo and 40 piano trio excerpts revealed that piano trio excerpts (*M* = 4.57, *SD* = .68) were indeed rated as more complex than piano solo excerpts (*M* = 3.93, *SD* = .65), *t*(78) = -4.34, *p* < .001, *r* = .44.

The inter-relationships between the four subjective variables (Table 13 and Figure 4) were generally similar to the ones observed for piano solo music in Experiment 3 (Table 9) when familiarity was not considered in the analysis, thus suggesting that the manipulation of complexity by changing the audible number of instruments in an auditory scene had a small effect on these inter-relationships. For instance, natural variations in complexity in piano solo music were strongly correlated to degrees of felt arousal, which was also the case when complexity was deliberately varied (*r*_s_ = .84). The findings further indicated significant positive correlations between familiarity and pleasantness in all three groups. Furthermore, the pattern of correlations between arousal and pleasantness was similar in direction as in Experiment 3. Specifically, the current results showed that arousal and pleasantness were negatively associated in females (*r*_s_ = -.29) but positively in males (*r*_s_ = .22), yielding a slightly negative correlation on average. Another notable finding concerns the positive relationship between complexity and pleasantness in males (*r*_s_ = .22) and a concurrent negative relationship in females (Figure S4), whereas in Experiment 3 complexity and pleasantness were not related in females. Moreover, the negative correlation between familiarity and arousal was stronger when all participants were considered in the analysis (*r*_s_ = -.24) compared to results presented in Experiment 3. In a similar vein, there were clear indications for a negative correlation between familiarity and complexity in males and females, which was not the case for females in Experiment 3.

**Table 13:** Spearman’s rank-order correlations between ratings of familiarity, complexity, pleasantness and arousal in response to set of piano solo and piano trio excerpts (N_All_ ≥ 76, n_males_ ≥ 74, n_females_ ≥ 75).

**Measure**		**Familiarity**	**Complexity**	**Pleasantness**
Complexity	f	-.12		
	m	-.18		
	All	-.21		
Pleasantness	f	.61*	-.26	
	m	.42*	.22	
	All	.67*	.02	
Arousal	f	-.15	.83*	-.29
	m	-.10	.76*	.22
	All	-.24	.84*	-.10

Note. * *p* < .05 after Bonferroni-Holm correction; f = females; m = males; All = all participants; the *dfs* are not the same for all correlations due to slightly different numbers of outliers.

Partial Spearman’s rank-order correlations controlling for effects of familiarity were computed in a next step (Table S10), and the results were more or less identical to the findings of Experiment 3, indicating that the observed differences in the inter-relationships between the subjective variables described above were not due to the different stimulus sets. Males showed a significant positive correlation between complexity and pleasantness (*r*_s_ = .41), whereas this relationship remained negative but not significant in females (*r*_s_ = -.14). The relationship between arousal and pleasantness got stronger only for males (*r*_s_ = .35) but not for females (*r*_s_ = -.23) when familiarity was considered in the analysis. The association between complexity and arousal also remained unchanged in all analyses after controlling for familiarity effects. In summary, it can be concluded that familiarity plays an indispensable role in the investigation of the inter-relationships between complexity, arousal and pleasantness. Most importantly, it seems that the relationship between complexity and pleasantness cannot be meaningfully discussed without considering gender. Future studies may investigate the underlying reasons of these gender effects and whether they hold true for other musical styles.

As in Experiments 1-3, a mediation analysis based on a bootstrapping approach [132] was performed in order to examine the presence of an indirect effect of complexity on pleasantness through arousal. Such an indirect effect would be expected according to Berlyne’s collative-motivation model [9]. First, a mediator model (arousal) was computed, and then a dependent variable model (pleasantness) was computed in a second step. Familiarity was specified as a covariate in both models. All relationships between the variables were modeled as linear. In general, the current results were very similar to those of Experiment 3. The model predicting the mediator was significant and explained 70% of the variance in arousal (Table 14). As expected, complexity had a strong significant positive impact on arousal (*a* = .76, *p* < .001). The dependent variable model was also significant and explained 54% of the variance in pleasantness. Furthermore, the results showed a significant direct effect of complexity on pleasantness after controlling for arousal (*c*’ = .20, *p* = .003), which we did not find in Experiments 1 and 2. The bootstrapped estimate of the indirect effect was negative and significant at the 95% confidence level, suggesting that pleasantness decreases linearly as complexity increases through its effect on arousal. As in Experiment 3, mediation models calculated for males and females separately revealed that this indirect effect was only significant in females, although both groups had similar musical listening backgrounds. We consider the replication of the results of Experiment 3, with partially different musical stimuli and a new sample of participants, to be a noteworthy finding. First, the observed gender effects with regard to the mediation effect seem to be robust when different styles of Romantic music are used as stimulus material. Second, the co-occurrence of significant direct and indirect effects may be a particularity of the musical domain because direct effects were not present in the current two experiments involving visual stimuli. Future studies employing different types of visual and musical stimuli under a common experimental framework may thus reveal whether the current findings can be generalized.

**Table 14:** Indirect effect of complexity (X) on pleasantness (Y) through arousal (M) with familiarity as a covariate, calculated for excerpts of piano solo music and piano trios (N_All_ = 76, n_males_ = 73, n_females_ = 72).

		**Model predicting arousal (M)**			
		***Coeff***	***SE***		
Constant	f	.49	.57		
	m	.007	.78		
	All	.83	.61		
Familiarity	f	.003	.15		
	m	.26	.22		
	All	-.07	.16		
Complexity (X)	f	.74***	.06		
	m	.76***	.07		
	All	.76***	.06		
Summary of model predicting M	f	*R* ^2^ = .69***			
	m	*R* ^2^ = .63***			
	All	*R* ^2^ = .70***			
		**Model predicting pleasantness (Y)**			
Constant	f	2.74***	.39		
	m	1.00	.57		
	All	1.83***	.37		
Familiarity	f	.73***	.10		
	m	.91***	.16		
	All	.84***	.10		
Complexity (X)	f	.15*	.07		
	m	.22*	.08		
	All	.20**	.06		
Arousal (M)	f	-.24**	.08		
	m	-.05	.09		
	All	-.16*	.07		
Summary of model predicting Y	f	*R* ^2^ = .48***			
	m	*R* ^2^ = .36***			
	All	*R* ^2^ = .54***			
		**Indirect effect**	**CI 95%**		
		f	-.18	-.31	-.05
		m	-.04	-.21	.08
		All	-.12	-.24	-.01

Note. X = independent variable; M = mediator variable; Y = dependent variable; *Coeff* = unstandardized coefficient; f = females; m = males; All = all participants; CI = confidence interval; * *p* < .05, ** *p* < .01, *** *p* < .001.

The Spearman’s rank-order correlations between subjective and objective complexity as measured by compressed file size (Table 15) revealed a positive association between all four variables. In comparison to results of Experiment 3, the magnitude of these correlations was somewhat larger, with the exception of event density (*r*_s_ = .43), when piano trios and piano solo music were combined in one stimulus set. As in Experiment 3, the highest correlation was observed for the compression size based on the FLAC format (*r*_s_ = .78), explaining around 60% of the variance in the data. Bearing in mind that Streich’s model of musical complexity [50] reached a very similar performance with twelve complexity descriptors (around 60% without considering variables based on compression file size), this result clearly highlights the usefulness of compression file size in future models of musical complexity. The finding that event density was a stronger predictor of subjective complexity in Experiment 3 than in the present stimulus set may relate to the measure itself and how it is calculated. It is likely that the current event density algorithm performs worse when several instruments are present in an auditory scene. Consequently, this may point to a possible advantage of measures based on compressed file size over measures based on audio feature extraction, especially in cases in which several musical styles are mixed together. However, several measures based on feature extraction will need to be considered in future studies in order to make such a claim valid.

**Table 15:** Spearman’s rank-order correlations between compression algorithms applied to excerpts of piano solo and piano trio music, their event density and rated familiarity, complexity, pleasantness and arousal (N_All_ ≥ 71, n_males_ ≥ 70, n_females_ ≥ 71).

**Measure**		**MP3**	**Ogg Vorbis**	**FLAC**	**Event Density**
Familiarity	f	-.15	.02	-.12	.24
	m	-.18	-.10	-.23	.06
	All	-.22	-.13	-.22	.21
Complexity	f	.66*	.62*	.79*	.37*
	m	.63*	.55*	.70*	.47*
	All	.66*	.60*	.78*	.43*
Pleasantness	f	-.27	-.11	-.20	.20
	m	.09	.25	.03	.34*
	All	-.06	.06	-.07	.29
Arousal	f	.64*	.58*	.80*	.15
	m	.65*	.60*	.70*	.26
	All	.64*	.59*	.78*	.20

Note. * *p* < .05 after Bonferroni-Holm correction; f = females; m = males; All = all participants; the *dfs* are not the same for all correlations due to slightly different numbers of outliers.

Moreover, as observed in Experiment 3, the compressed file size measures and event density did not correlate solely with subjective complexity. The strength of the relationships between compressed file size and arousal were similar for the two data sets, whereas event density did not significantly correlate with arousal in Experiment 4. Interestingly, indications for a negative correlation between familiarity and the MP3 and FLAC file sizes were present in Experiments 3 and 4. In contrast to these findings, familiarity and event density correlated positively with each other in both experiments. In addition, the current data revealed negative correlations between pleasantness and the three types of compression file size in females, which was also present in Experiment 3. In Experiment 4, we further observed a significant positive correlation between event density and pleasantness in males (*r*_s_ = .34), an association which was already weakly present in all groups in Experiment 3. A summary of the inter-relationships between the objective measures of musical complexity applied to piano solo and piano trio excerpts is shown in Table S8.

Linear stepwise regression analyses were calculated to examine how subjective ratings of familiarity, arousal and pleasantness as well as objective measures of complexity explain variance in subjective complexity. In the model based on all participants (Table 16), familiarity was entered as a first step followed by six predictors in a second step. In contrast to findings of Experiment 3, familiarity was a significant predictor of subjective complexity (β = -.25, *p* = .048). The final model after four steps (adjusted *R* ^2^ = .80, *F*(4,59) = 62.52, *p* < .001) revealed three other significant predictors, namely arousal (β = .68, *p* < .001), event density (β = .24, *p* < .001) and MP3 file size (β = .17, *p* = .024). Separate regression analyses computed for both genders showed very similar results. The model for males, adjusted *R* ^2^ = .74, *F*(4,56) = 42.61, *p* < .001, yielded the following four predictors: familiarity (β = -.11, *p* = .126), arousal (β = .57, *p* < .001), event density (β = .21, *p* = .004) and MP3 file size (β = .26, *p* = .006). The pattern of results only changed minimally for females because MP3 file size did not significantly explain variance in subjective complexity. The model, adjusted *R* ^2^ = .77, *F*(3,59) = 69.58, *p* < .001, was based on the following predictors: familiarity (β = -.12, *p* = .066), arousal (β = .77, *p* < .001), and event density (β = .28, *p* < .001). Importantly, a similar pattern of results was observed in Experiment 3 when the groups of males and females were analyzed separately, with the exception that pleasantness did not emerge as a significant predictor of subjective complexity in the current models. Finally, a model of subjective complexity solely based on objective complexity measures was built (Table S11). The final model, adjusted *R* ^2^ = .67, *F*(3,63) = 44.77, *p* < .001, comprised three predictors: FLAC file size (β = .55, *p* < .001), MP3 file size (β = .28, *p* = .003) and event density (β = .21, *p* = .008). A comparison with results of Experiment 3 (Table S9) suggests that the significant predictors were the same for both types of stimulus sets. However, event density was a weaker predictor of subjective complexity when piano solo and piano trio excerpts were mixed in one stimulus set. The pattern of results did not change when models were computed separately for males and females.

**Table 16:** Summary of linear stepwise regression analysis for seven variables predicting subjective complexity ratings of piano solo and piano trio excerpts (N = 64).

**Variable**	***B***	***SEB***	**β**
Step 1			
Constant	6.32	1.03	
Familiarity	-.68	.34	-.25*
Adjusted *R* ^*2*^	.05		
*F*	4.10*		
Step 2			
Constant	.77	.71	
Arousal	.99	.08	.85***
Familiarity	-.10	.19	-.04
Adjusted *R* ^*2*^	.72		
*F*	84.79***		
*ΔR* ^*2*^	.67		
Step 3			
Constant	.97	.64	
Arousal	.90	.07	.77***
Event density	.20	.05	.25***
Familiarity	-.27	.17	-.10
Adjusted *R* ^*2*^	.78		
*F*	76.04***		
*ΔR* ^*2*^	.06		
Step 4			
Constant	-2.26	1.53	
Arousal	.79	.09	.68***
Event density	.19	.05	.24***
MP3 file size	4.72E-6	< .001	.17*
Familiarity	-.23	.17	-.08
Adjusted *R* ^*2*^	.80		
*F*	62.52***		
*ΔR* ^*2*^	.02		

Note. * *p* < .05, ** *p* < .01, ***p < .001, *B* = unstandardized regression coefficient; SE = standard error; β = standardized regression coefficient; *ΔR* ^2^ *=* difference in the proportion of variance explained.

## General Discussion

Research on subjective complexity and its relationship to hedonic value has been of substantial interest to the empirical aesthetics research community and beyond. However, the emotional content of the stimuli under investigation has been largely ignored [74], which may have limited the interpretations and generalizability of results. Here, we presented a cross-domain approach to the study of the relationship between subjective complexity, felt pleasantness and arousal in affective stimuli sets of environmental scenes (IAPS pictures), representational paintings, and music. Russell’s circumplex model of affect [18] served as a common framework for the selection of representative stimuli inducing a wide range of emotions. Furthermore, we also applied recent advances in computational measures of complexity to the prediction of subjective complexity in the visual domain, and most importantly, introduced these methodological approaches to the musical domain for the first time. In this endeavor, complexity, regarded as a multidimensional concept, was varied by changing the number of elements present in a visual or musical scene. Last, the possible role of gender in these relationships was explored.

In all four experiments, the current data provided convincing evidence for a positive association between subjective complexity and arousal, which is in line with Berlyne’s collative-motivation model [7,10]. When controlling for effects of familiarity, the weakest association was found for environmental scenes (*r*_s_ = .27), followed by representational paintings (*r*_s_ = .59) and music (*r*_s_ = .84). In the present study, environmental scenes and representational paintings were matched for semantic contents and for average brightness; therefore, it can be argued that the differences in strength of this relationship may not only refer to the type of modality but also to the degree of artistic quality and motivational relevance. In other words, for artworks we observed a stronger relationship between complexity and arousal. Future studies may examine this hypothesis by comparing different types of visual and auditory stimuli varying in their artistic quality.

Based on recent findings by Nadal et al. in the visual domain [16], that essentially argue for a multidimensional nature of complexity, we predicted a positive association between subjective complexity and pleasantness because complexity was manipulated by the number of elements present in a visual or auditory scene. This hypothesis stands in stark contrast to Berlyne’s proposed inverted-U relationship between these variables [7,9]. In general, we found indications of a negative association between complexity and pleasantness for both genders when viewing environmental scenes, but this relationship was modulated by gender when viewing representational paintings or listening to music. Furthermore, we did not find support for a positive association between subjective complexity and pleasantness for the four types of visual and musical stimuli when effects of familiarity were controlled for in the analysis. Nevertheless, analyzing the data for both genders separately revealed that subjective complexity and pleasantness are indeed positively associated in males in both experiments involving musical stimuli (piano solo music: *r*_s_ = .42; piano solo music combined with piano trios: *r*_s_ = .41) but not in females. It needs to be seen whether this result holds for different musical styles. These findings may suggest that there is no common trend for the relationship between complexity and pleasantness in the current framework of the present cross-domain study, although this was clearly the case for the relationship between complexity and arousal. Further experiments may explore whether these findings are due to the specific emotion model on which the selection of stimuli was based [18], or whether they can be replicated within the context of other affective approaches or with other measures of hedonic value, such as preference and beauty. Specifically, based on our results it can be surmised that not only complexity, but also hedonic value, should be treated as a multidimensional concept. However, this hypothesis would require a more thorough investigation of the complexity-hedonic value relationship, considering effects of familiarity and gender alike.

Our results also shed light on the question of whether the complexity-pleasantness relationship is mediated by arousal as proposed by Berlyne [7,10]. By applying mediation analyses based on a bootstrapping approach [132] we observed indirect effects of complexity on pleasantness through arousal in all four experiments, which lends support to Berlyne’s theory [7,10]. In the visual domain, arousal was identified as a mediator in response to affective environmental scenes. No direct effect of complexity on pleasantness was identified in this case. The findings for representational paintings somewhat differed because the indirect effect was only identified when males and females were analyzed separately, probably due to differential effects of familiarity. The indirect effect was only significant for paintings moderate and high in complexity but not for paintings of low complexity. As was the case in the experiment involving environmental scenes, we did not find indications of a direct effect of complexity on pleasantness with regard to representational paintings. In the musical domain, the path model investigating the complexity-arousal-pleasantness relationship revealed that arousal was a significant mediator only for female participants. Moreover, a significant direct effect of complexity on pleasantness was present, which was not the case in the visual domain. These findings may point towards a potentially interesting cross-domain difference between the underlying cognitive and neural mechanisms of the relationship between complexity, arousal and pleasantness when aspects of familiarity are controlled for. However, further studies are necessary to replicate these findings with different types of visual and musical stimuli, as well as with different types of hedonic measures.

If one considers adopting an affective approach to the study of complexity, including the concept of arousal, there are several issues that warrant attention: Arousal presumably may not form a uniform concept as described in Berlyne’s psychobiological model; instead, current behavioral and neuroscientific findings suggest that there may be different forms of affective arousal with related multiple underlying brain mechanisms. For example, energy arousal has been differentiated from tension arousal [142]. In line with this argument, a dissociation between the direction of change of different types of behavioral, psychophysiological and neural measures of arousal in response to a stimulus has been reported [58,143,146], which may be partly explicable by ongoing emotion regulation processes [147]. In spite of these potential difficulties associated with the incorporation of arousal in the study of complexity, the affect-as-information hypothesis (for a review, see 147) and the attributed role of arousal in evaluative judgments, cognitive processing and memory [148] strongly support the view that aesthetic theories ignoring the role of arousal may be incomplete [51,52,149]. Indeed, arousal has been identified as a predictor of preference in the visual [150] and musical [151,152] domains outside the context of Berlyne’s theory [9].

Measures based on compressed file size and edge detection algorithms have recently been applied to the study of visual complexity [74,102,104]. One of the main findings of the present study is that when complexity is manipulated through the number of elements present in a visual scene, certain measures of objective complexity are more suitable for environmental scenes than for visual art and vice versa. Importantly, this is not in line with Forsythe et al. [74], who found no significant differences for non-affective environmental scenes and representational paintings when exploring the relationships between subjective complexity and objective measures, such as JPEG and GIF compressed file size and perimeter detection, respectively. However, Forsythe et al. [74] did not vary complexity along one specific dimension in their stimuli set, which limits the comparison with our current results. Nevertheless, our findings indicate that the performance of compression file size and different measures of edge detection may depend on how complexity is manipulated in the visual materials.

Specifically, the findings obtained in the present study showed that compressed file size only correlated positively with subjective complexity judgments of photographs of environmental scenes but not with complexity judgments of representational paintings. TIFF (*r*_s_ = .53) and JPEG (*r*_s_ = .52) compression file sizes yielded the strongest correlations with subjective complexity ratings of environmental scenes. In contrast to Forsythe et al. [74], the GIF compression format yielded the lowest correlation (*r*_s_ = .29). The moderate correlation between JPEG compression size and subjective complexity was also reported by Cavalcante et al. [104], suggesting that JPEG may be a reliable measure of objective complexity across different types of stimulus sets of environmental scenes and taken as a proxy for subjective ratings. However, none of the four compression file formats correlated significantly with subjective complexity judgments of representational paintings. At first sight, this finding is rather surprising since it is not in line with previous reports [74]. Nevertheless, it may be explained by the way complexity was manipulated in the current stimuli sets. A uniform background perceived as simple may be easily compressible in photographs of environmental scenes but not in representational paintings, in which the structure of the background does not necessarily contain a high amount of redundant information. For example, some backgrounds in paintings of simple figure-ground compositions may consist of a large number of individual brush strokes that form a rather uniform background from a subjective perspective. Compared to a background of a uniform blue sky in a photograph, the background in a painting may be less compressible although it perceptually appears similarly simple. In brief, these findings suggest that digitized versions of visual art cannot be treated like photographs of environmental scenes in the discussion about the relationship between subjective complexity and compressed file size when other factors, such as semantic and affective contents, brightness, picture size, presentation time and the kind of complexity manipulation are controlled for.

In a similar vein, disparities between environmental scenes and representational paintings were noted when comparing the performance of a set of edge detection measures to predict subjective complexity. For environmental scenes, the mean and alpha RMS contrast measures yielded the strongest correlations (*r*_s_ ~ .59), followed by perimeter detection (*r*_s_ ~ .45) and the standard deviation of the RMS contrast measure (*r*_s_ = .43). The mean and beta phase congruency measures worked reasonably well with a correlation of *r*_s_ ~ .35. The pattern of results looks somewhat different for representational paintings. The standard deviation of the phase congruency measure performed best (*r*_s_ = .38), followed by the beta measure of phase congruency (*r*_s_ = .30), and the alpha and standard deviation of the RMS contrast measures (*r*_s_ ~ .25). Taken together, these results suggest that the correlation strength between edge detection measures and subjective complexity is stronger for photographs of environmental scenes than for paintings. This finding is in line with Forsythe et al. [74], who reported a stronger positive correlation for environmental scenes (*r*_s_ = .54) than for representational paintings (*r*_s_ = .37) with respect to perimeter detection. In the present study, perimeter detection did not yield correlations as strong as those reported by Forsythe et al. [74] since this measure only performed moderately well for environmental scenes but not for paintings.

Furthermore, the current results replicate and extend those by Cavalcante et al. [104], who found that RMS contrast measures outperformed the JPEG compression format in predicting subjective complexity. Our data also revealed a positive correlation of *r*_s_ ~ .60 for environmental scenes and a weaker association in the set of representational paintings. This finding indicates that RMS contrast measures perform better or equally well as measures such as perimeter detection and Canny edge detection, depending on the stimuli type under investigation. In a similar vein, edge detection based on phase congruency [123,153] provided satisfying correlations with subjective complexity for both environmental scenes and paintings. This measure has not been applied to the prediction of subjective complexity earlier, but our results clearly demonstrate its usefulness. The standard deviation of the phase congruency measure was the best correlate for subjective complexity judgments of paintings (*r*_s_ = .38), and the beta phase congruency measure worked similarly well for environmental scenes (*r*_s_ = .36). Further research may elucidate why these new measures of RMS contrast and phase congruency work better to predict subjective complexity for representational stimuli than previously used measures of edge detection.

It is interesting to note that we observed a negative correlation between the Canny edge detection measure and subjective complexity for representational paintings, (*r*_s_ = -.23). Canny edge detection is particularly sensitive to fine lines in an image; therefore, lines associated with brush strokes in a background of a simple figure-ground composition are detected, yielding a negative correlation with subjective complexity judgments. For environmental scenes, this correlation was positive and together with the correlation for the entropy measure it was among the weakest when the whole set of edge detection measures was considered. This stands in contrast to earlier studies which reported moderate positive correlations between subjective complexity and the Canny algorithm for different types of stimulus sets other than environmental scenes [102].

Previously it has been argued that objective measures of complexity are not related to subjective familiarity judgments of visual materials [102]. Our results generally support this finding. Nevertheless, we found indications of weak correlations between familiarity and measures of edge detection in environmental scenes as well as between familiarity and compression file sizes in representational paintings. Moreover, subjective arousal correlated with several edge detection measures (RMS contrast, phase congruency and Canny algorithm) in representational paintings. The latter finding may be due to a significant correlation between subjective complexity and arousal, which was more pronounced in the set of representational paintings compared to environmental scenes. With this in mind, it can be concluded that the use of edge detection measures can also be extended to predict other subjective measures besides subjective complexity and pleasantness in the future [74]. Conversely, compressed file size as a measure of complexity was not significantly associated with subjective ratings of arousal and pleasantness. This finding may be understood in light of the two types of objective complexity measures examined in this study: Compressed file size represents a rather abstract measure of information contents, whereas edge detection measures are more concrete and object-related.

The current study convincingly demonstrated that advances in research on the relationship between subjective and objective complexity in the visual domain can be successfully applied to the musical domain. In two experiments, the ratio between the original and the compressed file size was shown to be an appropriate correlate of subjective complexity. One experiment investigated this relationship between subjective and objective complexity in a set of Romantic piano solo music excerpts, whereas another experiment used a set of musical excerpts in which musical complexity was varied by the number of instruments present in an auditory scene, i.e., by adding excerpts of piano trio music to those of piano solo music. In fact, the obtained positive correlations were generally stronger than the ones observed in the visual domain, and this regardless of whether complexity was manipulated or naturally present in a set of affective musical stimuli.

In analogy to edge detection measures, we also computed the event density of musical excerpts as a potential correlate of subjective complexity judgments and obtained moderate correlations for the set of piano solo music and the set of piano trios combined with piano solo music. However, event density yielded stronger correlations when only one musical genre was considered. It is important to note that compressed file size and event density correlated better with subjective complexity than any of the 12 predictors of Streich’s model of musical complexity [50], in which the best predictor yielded a moderate correlation of *r* = .42. Further research may thus investigate the potential application of compressed file size as a predictor of subjective complexity, as well as the one of event density, in stimuli sets comprising several musical styles.

Moreover, compressed file size of musical excerpts correlated strongly with subjective arousal, a finding which was also observed with regard to edge detection measures of representational paintings. It can be conjectured that the relationship between arousal and measures of objective complexity may be specific to artistic stimuli. Compressed file size and event density further correlated with familiarity and pleasantness in music, but these correlations were much weaker than the ones observed with arousal and sometimes only present in one gender. Nevertheless, these results clearly demonstrate that measures of objective complexity can be used to predict other aesthetic or affective responses in the musical domain, which extends the application of these measures. For instance, complexity measures, such as compressed file size and event density, could be integrated into current attempts of modeling emotional responses to music [93,154].

Linear stepwise regression models controlling for effects of familiarity revealed that objective measures of complexity were better predictors of subjective complexity than subjective arousal only in the case of environmental scenes. In the visual domain, the models were moderately successful and yielded adjusted *R* ^*2*^ s between .40 and .51 with two or three predictors, whereas regression models of musical complexity were able to predict around 80% of the variance in subjective complexity with models based on four predictors. In general, predictive modeling employing only objective complexity measures without controlling for familiarity effects was much less successful. With regard to environmental scenes and representational paintings an adjusted *R* ^*2*^ of .25 was reported, while in the musical domain higher values of .62 and .67 were reached. In other words, these results suggest that the current approach taken in predicting subjective complexity in the musical domain is very promising, especially since earlier models of musical complexity proposed by Streich [50] and Mauch and Levy [115] achieved similar results by following a computationally more demanding method.

The multimodal nature of perception, and especially interactions between the visual and auditory domains, has been recently highlighted in the study of sensory processing [155,156]. While affective interactions between the musical and visual domain have already been investigated [83,157,158], direct comparisons between the processing of visual and musical complexity are scarce. Our findings that conceptually similar objective measures of complexity (compression file size and event/object density) can be successfully applied to both the visual and musical domain are in line with results reported by Boon et al. [112]. Boon et al. [112] conducted computational analyses of fractal dimension and entropy of abstract art works by Jackson Pollock and music by Johann Sebastian Bach, showing that the fractal nature of art is present across domains. Taken together, these findings add to the discussion whether the processing of complexity in the visual and musical domains, although at first sight so different in nature, should be studied within a common framework in the future.

Another noteworthy finding of the present study was the observation of gender effects. We found substantial evidence for gender effects regarding the inter-relationships of the subjective ratings of familiarity, complexity, pleasantness and arousal and for the relationships of these variables with measures of objective complexity in both the visual and musical domain. Interestingly, gender effects were more frequent in the three experiments using artistic stimuli compared to the experiment involving environmental scenes. These effects not only concerned differences with respect to the magnitude of an association, but, more importantly, also the direction of a relationship, i.e., positive versus negative. In general, this is in line with previous research on emotional and aesthetic responses that has shown differential effects of gender in the visual [19,83,85,86,139,140], and musical [20] domains. The reasons for gender effects in affective and aesthetic responses may be manifold and related to biological and sociocultural factors. For example, gender effects may be due to females’ hypersensitivity to negative affective stimuli [19,20,87,159], or be explicable by the empathizer-systemizer theory [160] and the hunter-gatherer hypothesis [161]. Equally important, gender differences have been reported in the study of low-level perceptual processes, such as those involved in color perception [162], which in turn may influence the perception of affective visual stimuli. Further cross-domain research is clearly warranted to elucidate the role of gender in affective and aesthetic responses.

## Conclusions

Although emotions play a crucial role in the processing of visual and musical stimuli in everyday life, subjective complexity and its relation to hedonic value have not been extensively investigated within the context of affective stimuli and emotion models so far. By comparing responses to affective environmental scenes, representational paintings and music, we found cross-domain evidence for a positive relationship between subjective complexity and arousal, which is in line with Berlyne’s collative-motivation model [7,9]. In general, the data did not reveal a significant association between subjective complexity and pleasantness for affective stimuli when effects of familiarity were controlled for, which supports neither the predictions made by Berlyne [7,9] nor those of Nadal et al. [16]. A significant positive association between complexity and pleasantness was only observed in males with regard to two types of musical stimuli used in this study.

Recent advances in the study of objective complexity in the visual domain have been extended by incorporating new computational measures of compression file size and edge detection. Results indicated that the performance of these measures in predicting subjective complexity depends on the stimulus type (environmental scenes vs. representational paintings) when complexity is manipulated by the number of elements present in a visual scene. We also demonstrated that compressed file size and event density are useful predictors of subjective musical complexity in two sets of musical stimuli. Furthermore, we found instances of weak correlations between measures of objective complexity with familiarity pleasantness and arousal ratings, and these correlations seem partly to depend on gender and stimulus type. In summary, these findings are consistent with the view that only a cross-domain approach may lead to the development of a general theory about the relationship between emotion and complexity that incorporates aesthetic quality in its framework.

## Supporting Information

Figure S1 **Relationships between pleasantness, arousal and complexity, analyzed for males and females, in a set of IAPS pictures.** Low numbers refer to low ratings of pleasantness, arousal and complexity, respectively. A) Relationship between pleasantness and arousal for females. B) Relationship between complexity and arousal for females. C) Relationship between complexity and pleasantness for females. D) Relationship between pleasantness and arousal for males. E) Relationship between complexity and arousal for males. F) Relationship between complexity and pleasantness for males.
(TIF)

Figure S2 **Relationships between pleasantness, arousal and complexity, analyzed for males and females, in a set of representational paintings.** Low numbers refer to low ratings of pleasantness, arousal and complexity, respectively. A) Relationship between pleasantness and arousal for females. B) Relationship between complexity and arousal for females. C) Relationship between complexity and pleasantness for females. D) Relationship between pleasantness and arousal for males. E) Relationship between complexity and arousal for males. F) Relationship between complexity and pleasantness for males.
(TIF)

Figure S3 **Relationships between pleasantness, arousal and complexity, analyzed for males and females, in a set of piano solo music excerpts.** Low numbers refer to low ratings of pleasantness, arousal and complexity, respectively. A) Relationship between pleasantness and arousal for females. B) Relationship between complexity and arousal for females. C) Relationship between complexity and pleasantness for females. D) Relationship between pleasantness and arousal for males. E) Relationship between complexity and arousal for males. F) Relationship between complexity and pleasantness for males.
(TIF)

Figure S4 **Relationships between pleasantness, arousal and complexity, analyzed for males and females, in a set of piano solo and piano trio excerpts.** Low numbers refer to low ratings of pleasantness, arousal and complexity, respectively. A) Relationship between pleasantness and arousal for females. B) Relationship between complexity and arousal for females. C) Relationship between complexity and pleasantness for females. D) Relationship between pleasantness and arousal for males. E) Relationship between complexity and arousal for males. F) Relationship between complexity and pleasantness for males.
(TIF)

Table S1 **Partial Spearman’s rank-order correlations between ratings of complexity, pleasantness and arousal, controlling for effects of familiarity, in response to IAPS pictures (*N* = 96).** Delete this.
(PDF)

Table S2 **Spearman’s rank-order correlations between a representative set of measures of objective complexity applied to a set of IAPS pictures (*N* = 96).**
(PDF)

Table S3 **Summary of linear stepwise regression analysis for seven variables of objective complexity predicting subjective complexity ratings of IAPS pictures (*N* = 72).**
(PDF)

Table S4 **Partial Spearman’s rank-order correlations, controlling for effects of familiarity, between ratings of complexity, pleasantness and arousal in response to representational paintings (*N* = 96).**
(PDF)

Table S5 **Spearman’s rank-order correlations between a representative set of measures of objective complexity applied to a set of representational paintings (*N* =96).**
(PDF)

Table S6 **Summary of linear stepwise regression analysis for seven variables of objective complexity predicting subjective complexity ratings of representational paintings (*N* = 64).**
(PDF)

Table S7 **Partial Spearman’s rank-order correlations controlling for effects of familiarity, between ratings of complexity, pleasantness and arousal in response to piano solo music (*N* = 92).**
(PDF)

Table S8 **Spearman’s rank-order correlations between four measures of objective complexity applied to a set of piano solo excerpts (*N* = 92) and to a set of piano solo music excerpts combined with piano trio excerpts (*N* = 80).**
(PDF)

Table S9 **Summary of linear stepwise regression analysis for four variables of objective complexity predicting subjective complexity of piano solo music (*N* = 79).**
(PDF)

Table S10 **Partial Spearman’s rank-order correlations, controlling for effects of familiarity, between ratings of complexity, pleasantness and arousal in response to piano solo and piano trio excerpts (*N* = 80).**
(PDF)

Table S11 **Summary of linear stepwise regression analysis for four variables of objective complexity predicting subjective complexity ratings of piano solo and piano trio excerpts (*N* = 67).**
(PDF)

Stimulus List S1 **List of 96 IAPS pictures.**
(PDF)

Stimulus List S2 **List of 96 representational paintings.**
(PDF)

Stimulus List S3 **List of 92 Romantic piano solo music excerpts.**
(PDF)

Stimulus List S4 **List of 40 Romantic piano chamber music excerpts.**
(PDF)
