# Supplementary figures and images for: Examining Complexity across Domains: Relating Subjective and Objective Measures of Affective Environmental Scenes, Paintings and Music
Source: PLoS One. 2013 Aug 16;8(8):e72412. doi: 10.1371/journal.pone.0072412 (PMC3745471; doi:10.1371/journal.pone.0072412)

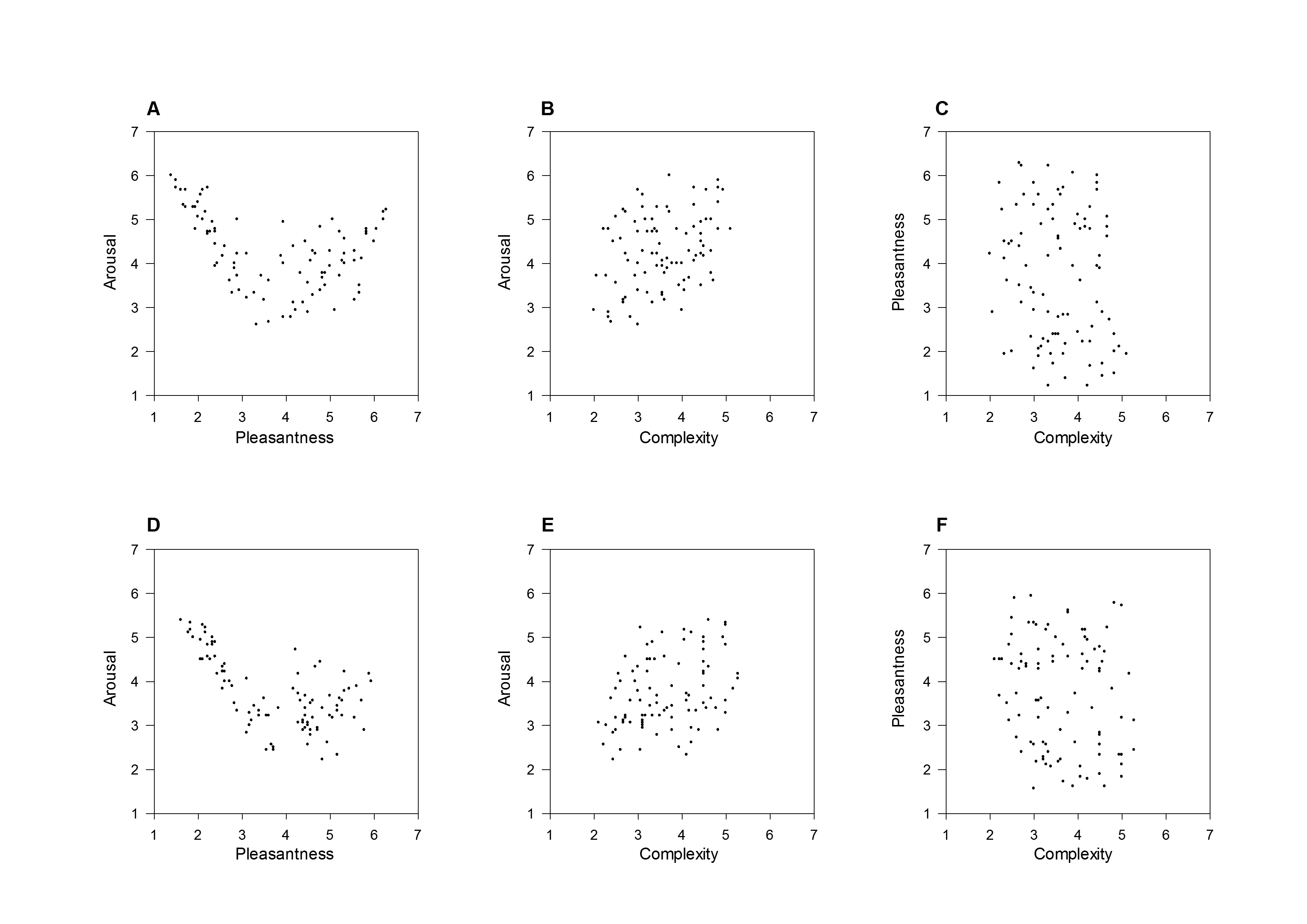

Supplement: Figure S1 — Relationships between pleasantness, arousal and complexity, analyzed for males and females, in a set of IAPS pictures. Low numbers refer to low ratings of pleasantness, arousal and complexity, respectively. A) Relationship between pleasantness and arousal for females. B) Relationship between complexity and arousal for females. C) Relationship between complexity and pleasantness for females. D) Relationship between pleasantness and arousal for males. E) Relationship between complexity and arousal for males. F) Relationship between complexity and pleasantness for males. (TIF) [file pone.0072412.s001.tif]

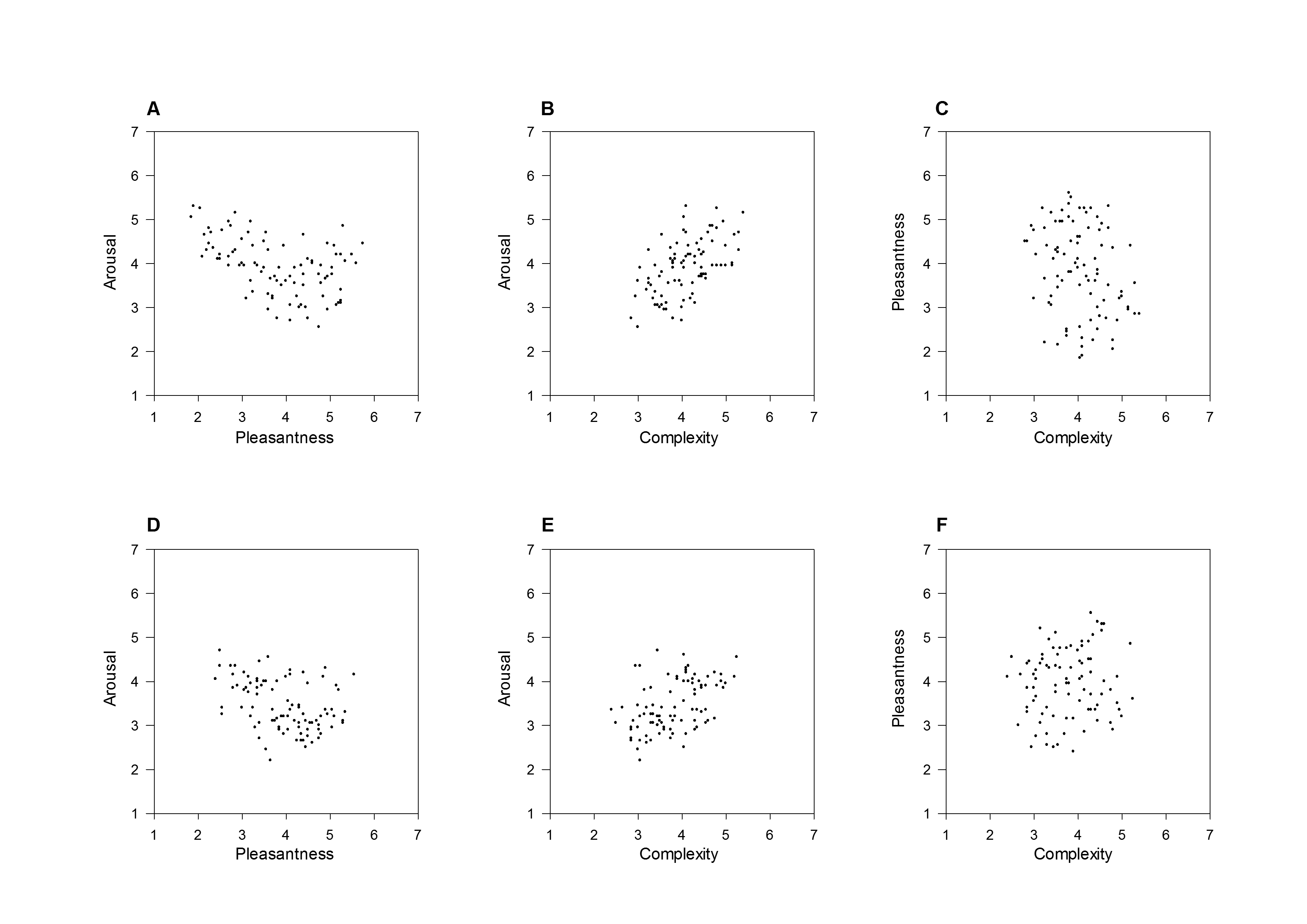

Supplement: Figure S2 — Relationships between pleasantness, arousal and complexity, analyzed for males and females, in a set of representational paintings. Low numbers refer to low ratings of pleasantness, arousal and complexity, respectively. A) Relationship between pleasantness and arousal for females. B) Relationship between complexity and arousal for females. C) Relationship between complexity and pleasantness for females. D) Relationship between pleasantness and arousal for males. E) Relationship between complexity and arousal for males. F) Relationship between complexity and pleasantness for males. (TIF) [file pone.0072412.s002.tif]

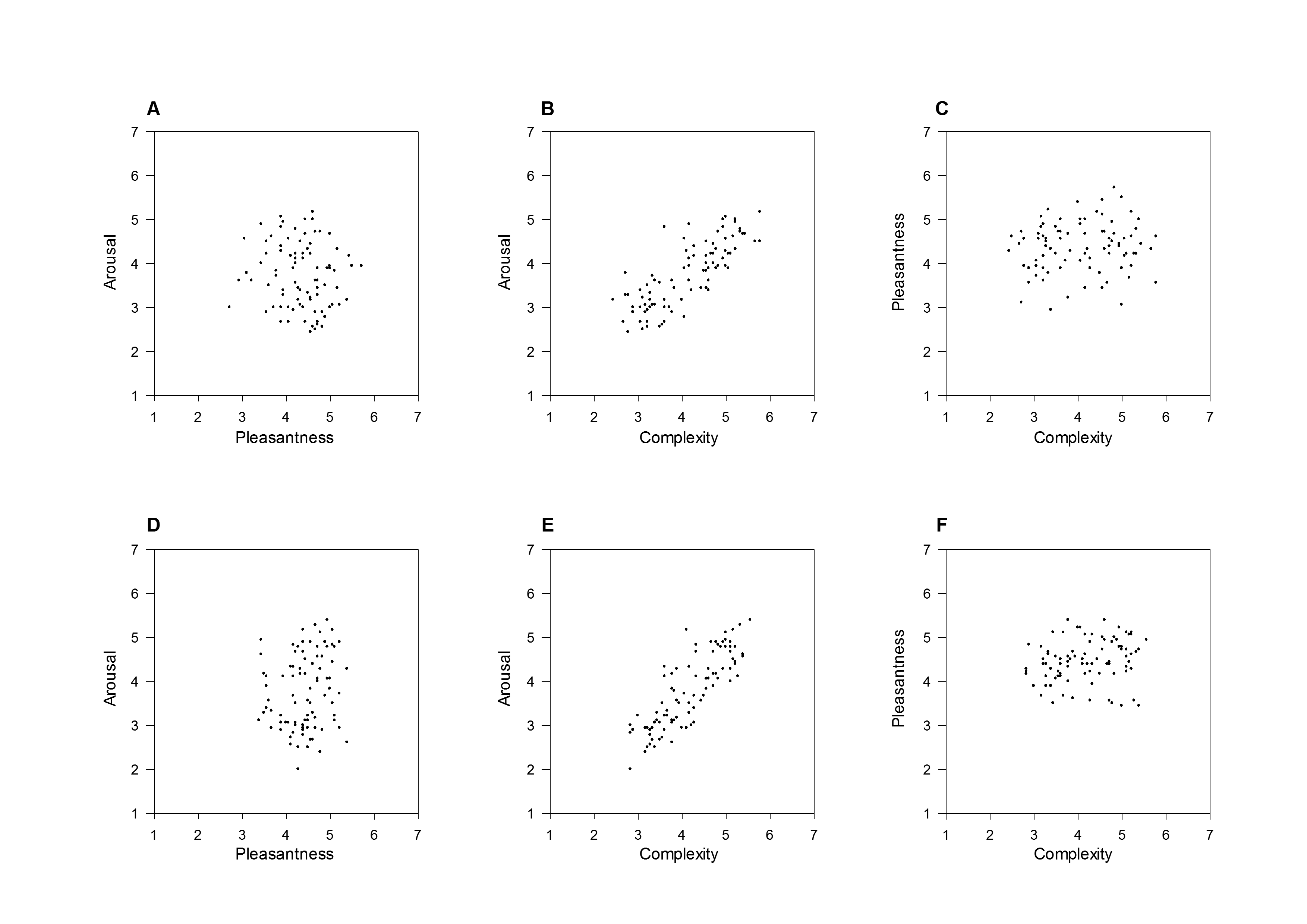

Supplement: Figure S3 — Relationships between pleasantness, arousal and complexity, analyzed for males and females, in a set of piano solo music excerpts. Low numbers refer to low ratings of pleasantness, arousal and complexity, respectively. A) Relationship between pleasantness and arousal for females. B) Relationship between complexity and arousal for females. C) Relationship between complexity and pleasantness for females. D) Relationship between pleasantness and arousal for males. E) Relationship between complexity and arousal for males. F) Relationship between complexity and pleasantness for males. (TIF) [file pone.0072412.s003.tif]

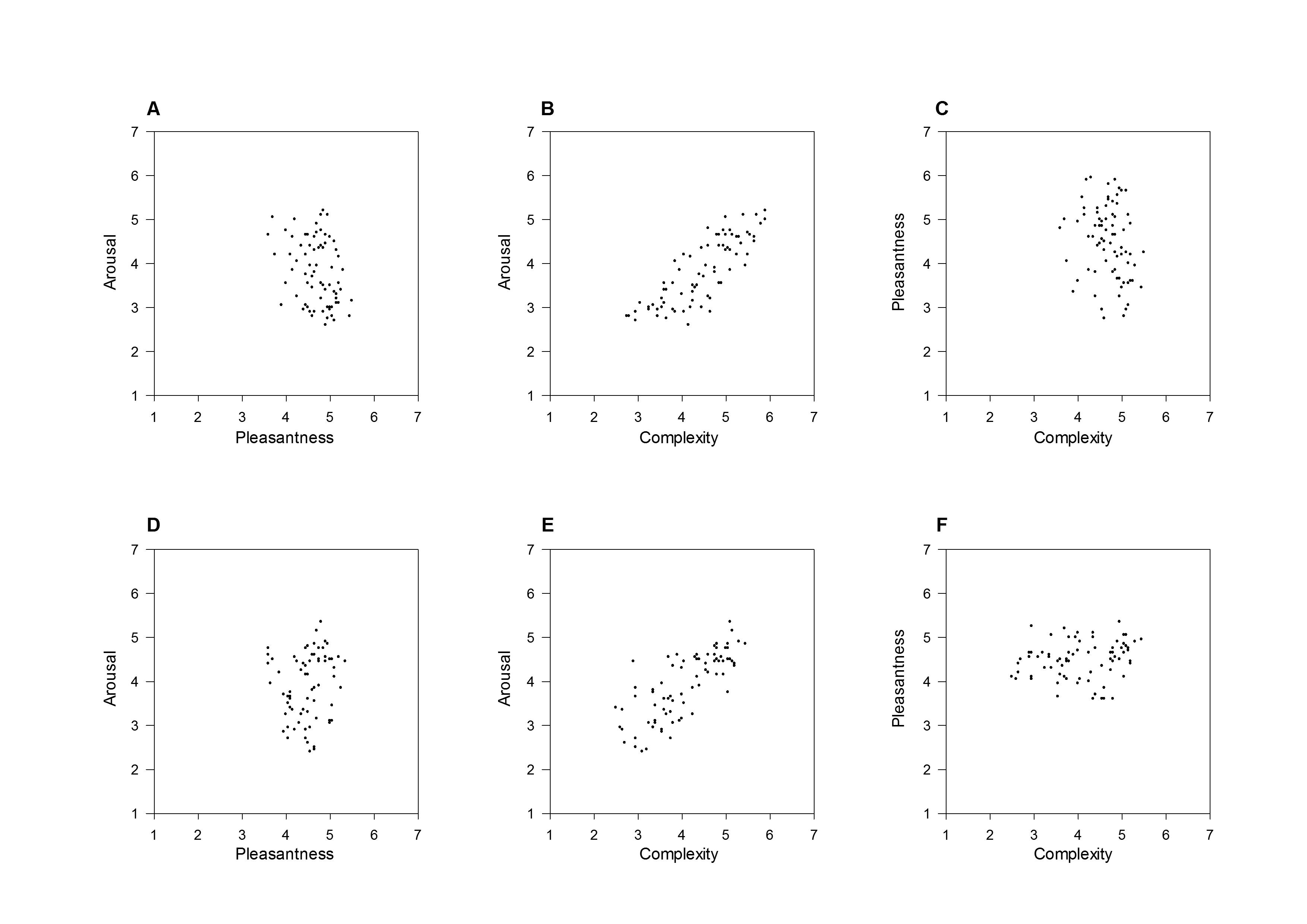

Supplement: Figure S4 — Relationships between pleasantness, arousal and complexity, analyzed for males and females, in a set of piano solo and piano trio excerpts. Low numbers refer to low ratings of pleasantness, arousal and complexity, respectively. A) Relationship between pleasantness and arousal for females. B) Relationship between complexity and arousal for females. C) Relationship between complexity and pleasantness for females. D) Relationship between pleasantness and arousal for males. E) Relationship between complexity and arousal for males. F) Relationship between complexity and pleasantness for males. (TIF) [file pone.0072412.s004.tif]
