# Supplementary material for: Examining Complexity across Domains: Relating Subjective and Objective Measures of Affective Environmental Scenes, Paintings and Music
Source: PLoS One. 2013 Aug 16;8(8):e72412. doi: 10.1371/journal.pone.0072412 (PMC3745471; doi:10.1371/journal.pone.0072412)
Supplement: Table S1 — Partial Spearman’s rank-order correlations between ratings of complexity, pleasantness and arousal, controlling for effects of familiarity, in response to IAPS pictures (N = 96). Delete this. (PDF) [file pone.0072412.s005.pdf]

Table S1

*Partial Spearman's rank-order correlations between ratings of complexity, pleasantness and arousal, controlling for effects of familiarity, in response to IAPS pictures (N = 96).*

| Group | | Complexity |
|---|---|---|
| f (*df* = 89) | Pleasantness | .02 |
| m (*df* = 91) | | .01 |
| All (*df* = 89) | | .01 |
| f (*df* = 89) | Arousal | .24* |
| m (*df* = 89) | | .25* |
| All (*df* = 91) | | .27* |

*Note.* **p* < .05 after Bonferroni-Holm correction; f = females; m = males; All = all participants; the *dfs* are not the same for all correlations due to slightly different numbers of outliers.
