# Supplementary material for: Examining Complexity across Domains: Relating Subjective and Objective Measures of Affective Environmental Scenes, Paintings and Music
Source: PLoS One. 2013 Aug 16;8(8):e72412. doi: 10.1371/journal.pone.0072412 (PMC3745471; doi:10.1371/journal.pone.0072412)
Supplement: Table S2 — Spearman’s rank-order correlations between a representative set of measures of objective complexity applied to a set of IAPS pictures (N = 96). (PDF) [file pone.0072412.s006.pdf]

Table S2

Spearman's rank-order correlations between a representative set of measures of objective complexity applied to a set of IAPS pictures (N = 96).

Measure	JPEG	GIF	PNG	TIFF	PERI-RAW	CANNY-RAW	ENTROPY	RMS-CONTR *M*
GIF	.81*							
PNG	.98*	.81*						
TIFF	.95*	.75*	.97*					
PERI	.84*	.69*	.78*	.76*				
CANNY	.61*	.59*	.59*	.56*	.56*			
ENTROPY	.46*	.54*	.46*	.48*	.40*	.31*		
RMS-CONTR *M*	.87*	.64*	.79*	.84*	.81*	.51*	.45*	
PHASE-CONG *M*	.47*	.32*	.39*	.45*	.58*	.48*	.17	.66*

Note. *$p < .05$ after Bonferroni-Holm correction; PERI = raw measure of perimeter detection; CANNY = raw measure of Canny edge detection; RMS = root mean square; CONTR = contrast; CONG = congruency; all *dfs* ≥ 81; the *dfs* are not the same for all correlations due to slightly different numbers of outliers.
