# Supplementary material for: Examining Complexity across Domains: Relating Subjective and Objective Measures of Affective Environmental Scenes, Paintings and Music
Source: PLoS One. 2013 Aug 16;8(8):e72412. doi: 10.1371/journal.pone.0072412 (PMC3745471; doi:10.1371/journal.pone.0072412)
Supplement: Table S3 — Summary of linear stepwise regression analysis for seven variables of objective complexity predicting subjective complexity ratings of IAPS pictures (N = 72). (PDF) [file pone.0072412.s007.pdf]

Table S3

Summary of linear stepwise regression analysis for seven variables of objective complexity predicting subjective complexity ratings of IAPS pictures (N = 72).

Variable	*B*	*SE B*	β
Constant	2.52	.23	
RMS contrast *M*	20.05	3.76	.54***
Adjusted R^2	.28		
F	28.37***		

Note. *p < .05, **p < .01, ***p < .001; *B* = unstandardized regression coefficient; *SE* = standard error; ß = standardized regression coefficient.
