# Supplementary material for: Examining Complexity across Domains: Relating Subjective and Objective Measures of Affective Environmental Scenes, Paintings and Music
Source: PLoS One. 2013 Aug 16;8(8):e72412. doi: 10.1371/journal.pone.0072412 (PMC3745471; doi:10.1371/journal.pone.0072412)
Supplement: Table S4 — Partial Spearman’s rank-order correlations, controlling for effects of familiarity, between ratings of complexity, pleasantness and arousal in response to representational paintings (N = 96). (PDF) [file pone.0072412.s008.pdf]

Table S4

Partial Spearman's rank-order correlations, controlling for effects of familiarity, between ratings of complexity, pleasantness and arousal in response to representational paintings (N = 96).

Group		Complexity
f (*df* = 85)	Pleasantness	.01
m (*df* = 84)		-.05
All (*df* = 83)		-.08
f (*df* = 85)	Arousal	.50*
m (*df* = 84)		.51*
All (*df* = 83)		.59*

Note. *$p < .05$ after Bonferroni-Holm correction; f = females; m = males; All = all participants; the *dfs* are not the same for all correlations due to slightly different numbers of outliers.
