# Supplementary material for: Examining Complexity across Domains: Relating Subjective and Objective Measures of Affective Environmental Scenes, Paintings and Music
Source: PLoS One. 2013 Aug 16;8(8):e72412. doi: 10.1371/journal.pone.0072412 (PMC3745471; doi:10.1371/journal.pone.0072412)
Supplement: Table S5 — Spearman’s rank-order correlations between a representative set of measures of objective complexity applied to a set of representational paintings (N =96). (PDF) [file pone.0072412.s009.pdf]

Table S5

Spearman's rank-order correlations between a representative set of measures of objective complexity applied to a set of representational paintings (N =96).

Measure	JPEG	GIF	PNG	TIFF	PERI	CANNY	ENTROPY	RMS-CONTR *SD*
GIF	.68*							
PNG	.89*	.68*						
TIFF	.97*	.71*	.94*					
PERI	.54*	.53*	.68*	.57*				
CANNY	.20	.28	.16	.20	.09			
ENTROPY	.32*	.39*	.39*	.36*	.39*	.14		
RMS-CONTR *SD*	.14	.12	.22	.15	.30	-.35*	.36*	
PHASE-CONG *SD*	-.15	-.23	-.10	-.13	.18	-.43*	-.07	.58*

Note. **p* < .05 after Bonferroni-Holm correction; PERI = raw measure of perimeter detection; CANNY = raw measure of Canny edge detection, RMS = root mean square, CONTR = contrast, CONG = congruency; all *dfs* ≥ 79; the *dfs* are not the same for all correlations due to slightly different numbers of outliers.
