# Supplementary material for: Examining Complexity across Domains: Relating Subjective and Objective Measures of Affective Environmental Scenes, Paintings and Music
Source: PLoS One. 2013 Aug 16;8(8):e72412. doi: 10.1371/journal.pone.0072412 (PMC3745471; doi:10.1371/journal.pone.0072412)
Supplement: Table S6 — Summary of linear stepwise regression analysis for seven variables of objective complexity predicting subjective complexity ratings of representational paintings (N = 64). (PDF) [file pone.0072412.s010.pdf]

Table S6

Summary of linear stepwise regression analysis for seven variables of objective complexity predicting subjective complexity ratings of representational paintings (N = 64).

Variable	*B*	*SE B*	β
Constant	-.06	.89	
Phase congruency *SD*	57.66	12.45	.69***
GIF file size	3.4E-6	< .001	.39**
RMS contrast *SD*	-21.01	9.94	-.30*
Adjusted R^2	.25		
F	7.9***		

Note. *p < .05, **p < .01, ***p < .001; *B* = unstandardized regression coefficient; *SE* = standard error; ß = standardized regression coefficient.
