# Supplementary material for: Examining Complexity across Domains: Relating Subjective and Objective Measures of Affective Environmental Scenes, Paintings and Music
Source: PLoS One. 2013 Aug 16;8(8):e72412. doi: 10.1371/journal.pone.0072412 (PMC3745471; doi:10.1371/journal.pone.0072412)
Supplement: Table S7 — Partial Spearman’s rank-order correlations controlling for effects of familiarity, between ratings of complexity, pleasantness and arousal in response to piano solo music (N = 92). (PDF) [file pone.0072412.s011.pdf]

Table S7

Partial Spearman's rank-order correlations, controlling for effects of familiarity, between ratings of complexity, pleasantness and arousal in response to piano solo music (N = 92).

Group		Complexity	Pleasantness
f (*df* = 80)	Pleasantness	-.002	
m (*df* = 81)		.42*	
All (*df* = 83)		.22	
f (*df* = 80)	Arousal	.82*	-.20
m (*df* = 81)		.82*	.28*
All (*df* = 83)		.83*	.03

Note. *$p < .05$ after Bonferroni-Holm correction; f = females; m = males; All = all participants; the *dfs* are not the same for all correlations due to slightly different numbers of outliers.
