# Supplementary material for: Examining Complexity across Domains: Relating Subjective and Objective Measures of Affective Environmental Scenes, Paintings and Music
Source: PLoS One. 2013 Aug 16;8(8):e72412. doi: 10.1371/journal.pone.0072412 (PMC3745471; doi:10.1371/journal.pone.0072412)
Supplement: Table S8 — Spearman’s rank-order correlations between four measures of objective complexity applied to a set of piano solo excerpts (N = 92) and to a set of piano solo music excerpts combined with piano trio excerpts (N = 80). (PDF) [file pone.0072412.s012.pdf]

Table S8

Spearman's rank-order correlations between four measures of objective complexity applied to a set of piano solo excerpts (N = 92) and to a set of piano solo music excerpts combined with piano trio excerpts (N = 80).

Measure		MP3	Ogg Vorbis	FLAC
Ogg Vorbis	Piano solo	.37*		
	Piano solo & piano trios	.44*		
FLAC	Piano solo	.59*	.58*	
	Piano solo & piano trios	.59	.67*	
Event Density	Piano solo	.07	.26*	.33*
	Piano solo & piano trios	.08	.28*	.30*

Note. **p* < .05 after Bonferroni-Holm correction; all *dfs* ≥ 80 for piano solo music; all *dfs* ≥ 68 for piano solo music & piano trios; the *dfs* are not the same for all correlations due to slightly different numbers of outliers.
