# Supplementary material for: Examining Complexity across Domains: Relating Subjective and Objective Measures of Affective Environmental Scenes, Paintings and Music
Source: PLoS One. 2013 Aug 16;8(8):e72412. doi: 10.1371/journal.pone.0072412 (PMC3745471; doi:10.1371/journal.pone.0072412)
Supplement: Table S9 — Summary of linear stepwise regression analysis for four variables of objective complexity predicting subjective complexity of piano solo music (N = 79). (PDF) [file pone.0072412.s013.pdf]

Table S9

Summary of linear stepwise regression analysis for four variables of objective complexity predicting subjective complexity of piano solo music (N = 79).

Variable	*B*	*SE B*	β
Step 1			
Constant	.17	.52	
FLAC file size	2.67E-6	<.001	.66**
Adjusted R^2	.43		
F	59.56***		
Step 2			
Constant	-.002	.45	
FLAC file size	2.13E-6	<.001	.53***
Event density	.32	.06	.42***
Adjusted R^2	.58		
F	54.82***		
ΔR^2	.15		
Step 3			
Constant	-3.53	1.27	
FLAC file size	1.37E-6	<.001	.34**
Event density	.35	.06	.46***
MP3 file size	6.05E-6	<.001	.27**
Adjusted R^2	.62		
F	43.12***		
ΔR^2	.04		

Note. *$p < .05$, **$p < .01$, ***$p < .001$; *B* = unstandardized regression coefficient; *SE* = standard error; ß = standardized regression coefficient; ΔR^2 = difference in the proportion of variance explained.
