# Supplementary material for: Examining Complexity across Domains: Relating Subjective and Objective Measures of Affective Environmental Scenes, Paintings and Music
Source: PLoS One. 2013 Aug 16;8(8):e72412. doi: 10.1371/journal.pone.0072412 (PMC3745471; doi:10.1371/journal.pone.0072412)
Supplement: Table S10 — Partial Spearman’s rank-order correlations, controlling for effects of familiarity, between ratings of complexity, pleasantness and arousal in response to piano solo and piano trio excerpts (N = 80). (PDF) [file pone.0072412.s014.pdf]

Table S10

Partial Spearman's rank-order correlations, controlling for effects of familiarity, between ratings of complexity, pleasantness and arousal in response to piano solo and piano trio excerpts (N = 80).

Group		Complexity	Pleasantness
f (*df* = 69)	Pleasantness	-.14	
m (*df* = 70)		.41*	
All (*df* = 73)		.25*	
f (*df* = 69)	Arousal	.83*	-.23*
M (*df* = 70)		.77*	.35*
All (*df* = 73)		.84*	.09

Note. *$p < .05$ after Bonferroni-Holm correction; f = females; m = males; All = all participants; the *dfs* are not the same for all correlations due to slightly different numbers of outliers.
