# Supplementary material for: Examining Complexity across Domains: Relating Subjective and Objective Measures of Affective Environmental Scenes, Paintings and Music
Source: PLoS One. 2013 Aug 16;8(8):e72412. doi: 10.1371/journal.pone.0072412 (PMC3745471; doi:10.1371/journal.pone.0072412)
Supplement: Table S11 — Summary of linear stepwise regression analysis for four variables of objective complexity predicting subjective complexity ratings of piano solo and piano trio excerpts (N = 67). (PDF) [file pone.0072412.s015.pdf]

Table S11

*Summary of linear stepwise regression analysis for four variables of objective complexity predicting subjective complexity ratings of piano solo and piano trio excerpts (N = 67).*

| Variable | *B* | *SE B* | β |
|---|---|---|---|
| Step 1 | | | |
| Constant | .85 | .35 | |
| FLAC file size | 2.00E-6 | <.001 | .77** |
| Adjusted $R^2$ | .59 | | |
| *F* | 96.87*** | | |
| Step 2 | | | |
| Constant | -3.72 | 1.65 | |
| FLAC file size | 1.60E-6 | <.001 | .62*** |
| MP3 file size | 6.95E-6 | <.001 | .26** |
| Adjusted $R^2$ | .63 | | |
| *F* | 57.63*** | | |
| $\Delta R^2$ | .05 | | |
| Step 3 | | | |
| Constant | -4.24 | 1.59 | |
| FLAC file size | 1.42E-6 | <.001 | .55** |
| MP3 file size | 7.34E-6 | <.001 | .28** |
| Event density | .16 | .06 | .21** |
| Adjusted $R^2$ | .67 | | |
| *F* | 44.77*** | | |
| $\Delta R^2$ | .04 | | |

*Note.* *$p < .05$, **$p < .01$, ***$p < .001$, *B* = unstandardized regression coefficient; *SE* = standard error; ß = standardized regression coefficient; $\Delta R^2$ = difference in the proportion of variance explained.
