# Supplementary material for: Examining Complexity across Domains: Relating Subjective and Objective Measures of Affective Environmental Scenes, Paintings and Music
Source: PLoS One. 2013 Aug 16;8(8):e72412. doi: 10.1371/journal.pone.0072412 (PMC3745471; doi:10.1371/journal.pone.0072412)
Supplement: Stimulus List S1 — List of 96 IAPS pictures. (PDF) [file pone.0072412.s016.pdf]

Stimulus List S1. List of 96 IAPS pictures (Lang, Bradley, & Cuthbert, 2005)

1090, 1300, 1333, 1340, 1440, 1500, 1540, 1610, 1640, 1670, 1710, 1850, 1900, 1920, 1930, 2053, 2070, 2104, 2110, 2120, 2205, 2306, 2340, 2493, 2501, 2506, 2513, 2560, 2590, 2650, 2690, 2691, 2703, 2791, 2800, 2900, 3180, 3181, 3530, 3550, 4574, 4617, 4641, 5200, 5201, 5480, 5600, 5621, 5811, 5833, 5890, 5970, 5971, 6020, 6212, 6230, 6370, 6821, 7330, 7350, 7352, 7380, 8041, 8080, 8090, 8116, 8120, 8161, 8180, 8200, 8280, 8370, 8400, 8465, 8485, 8531, 9000, 9001, 9008, 9090, 9101, 9110, 9140, 9181, 9220, 9265, 9280, 9290, 9342, 9360, 9560, 9561, 9592, 9630, 9912, 9921, 1460 (practice 1), 2410 (practice 2)
