# Supplementary material for: Examining Complexity across Domains: Relating Subjective and Objective Measures of Affective Environmental Scenes, Paintings and Music
Source: PLoS One. 2013 Aug 16;8(8):e72412. doi: 10.1371/journal.pone.0072412 (PMC3745471; doi:10.1371/journal.pone.0072412)
Supplement: Stimulus List S2 — List of 96 representational paintings. (PDF) [file pone.0072412.s017.pdf]

Stimulus List S2. List of 96 representational paintings

Adolf Lier	Sommerlandschaft am Starnberger See, 1857/59
Adolf Menzel	Kopf eines toten Schimmels, 1884
Adolf Menzel	Ratte im Rinnstein, 1863-1883
Anders Leonard Zorn	Mrs. Lucy Turner Joy, 1897
Andreas Achenbach	Italienische Landschaft in Küstennähe, 1845
Andreas Achenbach	Seesturm, 1890
Arnold Boecklin	Die Pest, 1898, upper part
Arthur Fitzwilliam	Maternal solicitude, 1873
Berthe Morisot	Reines-marguerites, 1885
Berthe Morisot	Julie rêveuse, 1894, upper part
Berthe Morisot	Jeune femme de dos à sa toilette, 1880
Camille Pissarro	Le Repos, paysanne couchée dans l'herbe, 1882
Carl Spitzweg	Der Sonntagsspaziergang, 1841
Caspar David	Landschaft mit Regenbogen, 1810
Caspar David	Flache Landschaft (Insel Rügen oder in der Nähe von Greifswald),
Caspar David	Das große Gehege, 1830-1834
Caspar David	Gebirgige Flusslandschaft bei Nacht, 1830/1835
Caspar David	Abtei im Eichwald, na
Caspar David	Kügelgens Grab, 1821/1822
Claude Monet	The river, Bennecourt, 1868
Claude Monet	Meule, effet de neige, le matin, 1891
Claude Monet	Les Coquelicots, 1873
Claude Monet	Femme au jardin, 1867
Claude Monet	La terrasse à Sainte-Adresse, 1867
Dora Koch-Stetter	Kleines Mädchen auf der Wiese, na
Edgar Degas	Eugène Monet, 1874
Edgar Degas	Portrait de Estelle Musson Degas, 1872
Edgar Degas	(Madame Paul Valpinçon?), 1865
Edgar Degas	La convalescente, 1868-1873, upper half
Edouard Manet	Le combat de taureaux, 1865-1866
Edouard Manet	Panier de fruits, 1864
Eugène Delacroix	Jeune orpheline au cimetière, 1824, upper half
Eugène Delacroix	Les deux chevaux morts sur le champ de bataille, 1824
Eugène Delacroix	Indienne dévorée par un tigre, 1840-1850
Francisco de Goya	Casa de locos, 1815/1819
Francisco de Goya	Pavo desplumado, 1810-1823
Francisco de Goya	La nevada, 1786
Francisco de Goya	Três de Maio de 1808 em Madrid, Os fuzilamentos da montanha do
Franz Marc	Die weiße Katze, 1912
Franz Marc	Sibirischer Hund, 1909

Franz von Defregger	Der Salon Tiroler, 1882
Franz von Lenbach	Ein Hirtenknabe, 1860
Frederic Remington	What an unbranded cow has cost, 1895
Friedrich Nerly	Die Piazzetta bei Mondschein, 1842
George Catlin	Wounded buffalo, strewing his blood over the prairies, 1832-1833
Gustave Courbet	L’orée de la forêt, 1856
Gustave Courbet	Fleurs dans un panier, 1863
Gustave Courbet	La dame au podoscaphe, 1865
Gustave Courbet	Les amants heureux, 1844, faces enlarged
Gustave Courbet	Juliette Courbet, soeur cadette du peintre, 1839
Gustave Courbet	Biche forcée, effet de neige, 1857
Gustave Courbet	La truite, 1872
Gustave Courbet	L’homme blessé, 1855
Gustave Courbet	Portrait de Paul Chenavard, 1869
Gustave Courbet	Zorro en la nieve, 1860
Gustave Courbet	Portrai de l’artiste, dit Le Dèsesperé
Gustave Courbet	Lutteurs, 1852-1853, upper half
Hugo Vogel	Am Strand, 1891
Jacques-Louis David	Assassination of Jean Paul Marat, 1793, body enlarged
James Goodwyn	The happy moment, 1847, person enlarged
Jean-François Millet	Narcisses et violettes, 1867
Joaquin Sorolla	¡Aún dicen que el pescado es caro!, 1894
Joseph Mallord	Dutch boards in a Gale: Fishermen endeavoring to put their fish on
Jules Elie Delaunay	La peste à Rome, 1869
Käte Lassen	Der Tod und das Mädchen, 1914
Mary Cassatt	Sleepy baby, 1910
Max Pechstein	Liegendes Mädchen, 1910
Nikolaj Alekseevic	Poor people picking coal in a deserted pit, 1894
Paul Cézanne	Baie de Marseille, vue de l'Estaque, 1885
Paul Cézanne	La neige fondue à l’Estaque, 1870-1871
Paul Cézanne	Le meurtre, 1868-1869
Paul Cézanne	Vieille femme au rosaire, 1900-1904, head enlarged
Paul Cézanne	Pyramide de crânes, 1900
Paul Gauguin	Montagnes dans Tahiti, 1891
Pierre-Auguste Renoir	Jeune fille endormie, 1880
Pierre-Auguste Renoir	L'après-midi des enfants à Wargemont, 1884
Pierre-Auguste Renoir	Chien, 1876
Pierre-Auguste Renoir	Portrait de Madame Henriot, 1876, upper half
Pierre-Auguste Renoir	Portrait of Mademoiselle Durand-Ruel, 1882, upper half
Robert Koehler	Head of an old woman, 1881, head enlarged
Robert Spear Dunning	Harvest of cherries, 1866
Sir Edwin Henry	Old shepherd's chief mourner, 1837

Théodore Géricault	L’homme aux illusions du commandement militaire, 1822-1823
Théodore Géricault	Le chat mort, 1820
Théodore Géricault	After death: study of the head of a corpse, 1819
Théodore Géricault	Le radeau de la Méduse, 1819
Théodore Géricault	Têtes de suppliciés, 1818
Theophile-Alexandre	Ausgestreckte Katze, 1910
Thomas Eakins	John Biglin in a single scull, 1874, person enlarged
Thomas Eakins	The Agnew clinic, 1889
Vincent van Gogh	La maison jaune, 1888
Vincent van Gogh	La plaine de Crau, 1888
Vincent van Gogh	Les mangeaurs de pommes de terre, 1885
William Sidney Mount	Fruit piece: Apples on tin cups, 1864
William Turner	Calais pier, with French poissards preparing for sea, 1803
Winslow Homer	Gulf stream, 1870/1900
Practice 1: Vincent van Gogh	Deux paysannes ramassant la tourbe, 1883
Practice 2: Franz von Defregger	Vor dem Tanz, 1872
