# Supplementary material for: Examining Complexity across Domains: Relating Subjective and Objective Measures of Affective Environmental Scenes, Paintings and Music
Source: PLoS One. 2013 Aug 16;8(8):e72412. doi: 10.1371/journal.pone.0072412 (PMC3745471; doi:10.1371/journal.pone.0072412)
Supplement: Stimulus List S3 — List of 92 Romantic piano solo music excerpts. (PDF) [file pone.0072412.s018.pdf]

Stimulus List S3. List of 92 Romantic piano solo music excerpts

Stimuli whose code is marked with an asterisk were used in Experiments 3 and 4. The first number of the code refers to the degree of arousal and pleasantness (1 = low-arousing pleasant, 2 = high-arousing pleasant, 3 = low-arousing unpleasant, and 4 = high-arousing unpleasant) based on findings by Marin, Gingras, & Bhattacharya (2012).

Code	Composer	Piece & CD	Duration
**101**	Robert Schumann	Fantasiestücke, Op. 12, No. 1, Philips, 434 732-2, Alfred Brendel, 1980	0:00 - 0:25.00
**102***	Fryderyk Chopin	Nocturne in D flat major, Op. 27, No. 2, Lento sostenuto, Deutsche Grammophon, 447 096-2, Maria João Pires, 1996	0:00 - 0:25.00
**103**	Franz Schubert	Piano Sonata 19 in C minor, D. 958, Mvt. 2, Adagio, Naxos, 8.550475, Jenö Jandó, 1991	0:00 - 0:25.00
**104**	Robert Schumann	Albumblätter, Op. 124, Mvt. 16, Schlummerlied, McGill Records, MR 2006-01-2, Kyoko Hashimoto, 2006	0:18 - 0:43.00
**105**	Fryderyk Chopin	Mazurkas, Op. 24, No. 3 in C major, Unterschrift Classics (no number), Chopin: Complete Mazurkas, Nina Milkina, 1970	0:00 - 0:25.00
**106**	Franz Liszt	Legenden, Franz von Assisi, Philips, 410 040-2, Alfred Brendel, 1991	7:54.60 - 8:19.60
**107***	Robert Schumann	Album für die Jugend, Op. 68, II, Mignon, Naxos, 8.555711, Rico Gulda, 2001	0:00 - 0:25.00
**108**	Fanny Mendelssohn-Hensle	Das Jahr, 12 Charakterstücke, No. 5, Mai, Sony Classical, 88697030162, Lauma Skirde, 2007	0:00 - 0:25.00
**109**	Edvard Grieg	Berceuse Op. 38, No. 1, Allegretto tranquillo, Deutsche Grammophon, 459671-2, Mikhail Pletnev, 2000	0:00 - 0:25.00
**110**	Johannes Brahms	Balladen, Op. 10, No. 3, Allegro in B, minor (Intermezzo), Thorofon, CTH 2241, Detlef Kraus, 1994	1:48.60 - 2:13.60
**111**	Franz Schubert	Drei Klavierstücke (3 Impromptus), D946, No. 2 in E-flat major, Harmonic Records, 8610, Paul Badura-Skoda, 1986	0:00 - 0:25.00
**112**	Felix Mendelssohn	Lieder ohne Worte, Op. 30, No. 3 in E major, Adagio non troppo, Deutsche Grammophon, 453 061-2, Daniel Barenboim, 1974	0:00 - 0:25.00
**113**	Felix Mendelssohn	Lieder ohne Worte, Op. 67, No. 3 in B flat major, Andante tranquillo, Deutsche Grammophon, 453 061-2, Daniel Barenboim, 1974	0:00 - 0:25.00

**114***	Felix Mendelssohn	Lieder ohne Worte, Op. 102, No. 6 in C major, Andante, Deutsche Grammophon, 453 061-2, Daniel Barenboim, 1974	0:00 - 0:25.00
**115***	Felix Mendelssohn	Kinderstücke Op. 72, No. 2, Andante sostenuto, Deutsche Grammophon, 453 061-2, Daniel Barenboim, 1974	0:00 - 0:25.00
**116***	Felix Mendelssohn	Lieder ohne Worte, Op. 85, No. 4 in D major, Andante sostenuto, Deutsche Grammophon, 453 061-2, Daniel Barenboim, 1974	0:00 - 0:25.00
**117***	Felix Mendelssohn	Gondellied, Allegretto non troppo, Deutsche Grammophon, 453 061-2, Daniel Barenboim, 1974	0:00 - 0:25.00
**118***	Edvard Grieg	Piano Sonata E minor, Op. 7, Andante molto, Deutsche Grammophon, 459671-2, Mikhail Pletnev, 2000	0:00 - 0:25.00
**119***	Franz Schubert	Sonata in B flat major, D 960, No. 2, Andante Sostenuto, Harmonic Records, H/CD 8610, Paul Badura-Skoda, 1986	0:51.00 - 1:16.00
**120***	Franz Schubert	Piano Sonata in A major, Op. Posth., D959, No. 2, Andantino, CBS Records, MK 44569, Murray Perahia, 1987	0:00 - 0:25.00
**121**	Fryderyk Chopin	Nocturne in B flat minor, Op. 9, No. 1, Larghetto, Deutsche Grammophon, 447 096-2, Maria João Pires, 1996	0:00 - 0:25.00
**122***	Johannes Brahms	3 Intermezzi, Op. 117, No. 1 in E flat major, Nimbus Records, NI 5376, Martin Jones, 1992	0:00 - 0:25.00
**123**	Fryderyk Chopin	2 Nocturnes, Op. 32, Nocturne No. 1 in B major, EMI Classics, 7243 5 56196 2 4, Byron Janis, 1996	0:00 - 0:25.00
**201**	Felix Mendelssohn	Piano Sonata 1 in B flat major, Op. 106, Mvt. 1, Allegro Vivace, Naxos, 8.553186, Benjamin Frith, 1997	0:08.00 - 0:33.00
**202**	Edvard Grieg	Fifth Birthday, Victoria, VCD 19025, Geir Henning Braaten, 1990	0:00 - 0:25.00
**203***	Franz Liszt	Schubert Transcriptions, Franz Schuberts Märsche für das Pianoforte übertragen; Grande Marche caractéristique, Hyperion, CDA66953, Leslie Howard, 1995	0:00 - 0:25.00
**204**	Felix Mendelssohn	Seven Characteristic Pieces, Op. 7, No. 7, Leicht und luftig, Naxos, 8.553541, Benjamin Frith, 1995	0:00 - 0:25.00
**205**	Franz Liszt	Legenden, Franz von Padua, Philips, 410 040-2, Alfred Brendel, 1991	4:56.00 - 5:21.00
**206***	Franz Schubert	Piano Sonata in A major, Op. Posth., D. 959, No. 3, Scherzo: Allegro vivace, CBS Records, MK 44569, Murray Perahia, 1987	0:00 - 0:25.00

**207***	Robert Schumann	Fantasiestücke, Op. 12, No. 7, Traumes Wirren, Philips, 434 732-2, Alfred Brendel, 1981	0:00 - 0:25.00
**208**	Johannes Brahms	16 Walzer, Op. 39, No. 1, Tempo guisto in B major, Thorofon, CTH 2176, Detlef Kraus, 1992	0:00 - 0:25.00
**209***	Johannes Brahms	16 Walzer, Op. 39, No. 6, Vivace in C sharp major, Thorofon, CTH 2176, Detlef Kraus, 1992	0:00 - 0:25.00
**210**	Franz Schubert	Impromptus D 935, No. 3 in B flat major, Thema-Andante, Var. I-V, Deutsche Grammophon, 435 788-2, Andrei Gavrilov, 1992	3:06.90 - 3:31.90
**211**	Franz Schubert	Sonata in B-flat major, D960, Scherzo, Allegro vivace con delicatezza, Harmonic Records, 8610, Paul Badura-Skoda, 1986	0:00 - 0:25.00
**212***	Felix Mendelssohn	Lieder ohne Worte, Op. 19, No. 3 in A major, Jägerlied, Deutsche Grammophon, 453 061-2 Daniel Barenboim, 1974	0:00 - 0:25.00
**213***	Felix Mendelssohn	Lieder ohne Worte, Op. 102, No. 3 in C major, Presto, Deutsche Grammophon, 453 061-2, Daniel Barenboim, 1974	0:00 - 0:25.00
**214**	Felix Mendelssohn	Zwei Klavierstücke, No. 2, Presto agitato in G minor, Naxos, 8.553358, Benjamin Frith, 1998	0:00 - 0:25.00
**215**	Robert Schumann	Klavierstücke, 29, Op. 32, Nuova Era, NE 7353, Jörg Demus, 1988	0:00 - 0:25.00
**216**	Robert Schumann	Etudes symphoniques, Op. 13, Etude IV (Variation III), EMI Classics, 7234 573277 2 5, Tzimon Barto, 1990	0:00 - 0:25.00
**217***	Robert Schumann	Kreisleriana, Op. 16, No. 7, Sehr rasch, Philips, 434 732-2 , Alfred Brendel, 1981	1:01.51 - 1:16.00
**218**	Felix Mendelssohn	Lieder ohne Worte, Op. 30, No. 2 in B flat minor, Allegro di molto, Deutsche Grammophon, 453 061-2, Daniel Barenboim, 1974	0:00 - 0:25.00
**219***	Felix Mendelssohn	Lieder ohne Worte, Op. 30, No. 4 in B minor, Agitato e con fuoco, Deutsche Grammophon, 453 061-2, Daniel Barenboim, 1974	0:00 - 0:25.00
**220***	Fryderyk Chopin	Piano Sonata in B flat, Op. 35, 1st movement, Decca, 00028943638920, Vladimir Ashkenazy, 1993	0:00 - 0:25.00
**221**	Felix Mendelssohn	Lieder ohne Worte, Op. 38, No. 3 in E major, Presto e molto vivace, Deutsche Grammophon, 453 061-2 Daniel Barenboim, 1974	0:00 - 0:25.00
**222**	Edvard Grieg	23 short pieces for piano (EG 104), Scherzo, Victoria, VCD 19034, Geir Henning Braaten, 1993	0:13.00 - 0:38.00

**223**	Felix Mendelssohn	Lieder ohne Worte, Op. 85, No. 3 in E major, Presto, Deutsche Grammophon, 453 061-2, Daniel Barenboim, 1974	0:00 - 0:25.00
**301**	Carl Maria von Weber	Piano Sonata 1 in C major, Op. 42, Mvt. 2, Adagio, Naxos, 8.550988, Alexander Paley, 1994	0:00 - 0:25.00
**302**	Robert Schumann	3 Fantasiestücke, Op. 111, No. 2 in A flat major, CPO, 999 431-2, Volker Banfield, 1997	0:15 – 40.00
**303***	Franz Liszt	Legenden, Franz von Padua, Philips, 410 040-2, Alfred Brendel, 1991	0:12.39 - 0:37.39
**304***	Robert Schumann	Gesänge der Frühe, 1-22, Nuova Era, 7353, Jörg Demus, 1988	0:41.63 - 1:06.63
**305**	Robert Schumann	Piano Sonata, in G minor, Op. 22, 2nd movement, Andantino, Sony Classical, 44569, Murray Perahia, 1986	0:00 - 0:25.00
**306**	Fryderyk Chopin	Mazurkas, No. 15 in F minor, Unterschrift Classics (no number), Chopin: Complete Mazurkas, Nina Milkina, 1970	0:00 - 0:25.00
**307**	P. I. Tchaikovsky	Romance in F minor, Op. 5, Naxos, 8.550504, Ilona Prunyi, 1991	0:00 - 0:25.00
**308**	Robert Schumann	Etudes symphoniques, Op. 13, Thema, EMI Classics, 5 69521 2, Dimitri Alexeev, 1996	0:22.00 - 0:47.0
**309**	Johannes Brahms	4 Balladen, Op. 10, Ballade No. 1 in D minor, Nimbus Records, 5372, Martin Jones, 1992	0:03.00 - 0:28.00
**310**	Johannes Brahms	Variations on a theme by Robert Schumann in F sharp minor, Op. 9, Nimbus Records, 5372, Martin Jones, 1992	0:00 - 0:25.00
**311***	Robert Schumann	Waldszenen, Op. 82, No. 3, Einsame Blume, MIREs 29-017, Denise Trudel, 2006	0:00 - 0:25.00
**312***	Franz Liszt	Die Trauergondel, No. 1, Philips, 410 040-2, Alfred Brendel, 1991	0:00 - 0:25.00
**313**	Fanny Mendelssohn-Hensel	Das Jahr, 12 Charakterstücke, No. 6, Juni, Sony Classical, 88697030162, Lauma Skirde, 2007	2:43.53 - 3:08.53
**314***	Robert Schumann	Album für die Jugend, Op. 68, II, Winterzeit 1, Naxos, 8.555711, Rico Gulda, 2001	0:00 - 0:25.00
**315***	Felix Mendelssohn	Lieder ohne Worte, Op. 62, No. 5 in A minor, Venezianisches Gondellied, Deutsche Grammophon, 453 061-2, Daniel Barenboim, 1974	0:00 - 0:25.00
**316***	Fanny Mendelssohn-Hensel	Das Jahr, 12 Charakterstücke, No. 1, Jänner, Sony Classical, 88697030162, Lauma Skirde, 2007	0:00 - 0:25.00

**317**	Fryderyk Chopin	Nocturne No. 13 in C minor, Deutsche Grammophon, 477 0962 GH2, Maria Joao Pires, 1996	0:00 - 0:25.00
**318**	Fryderyk Chopin	Préludes, Op. 28, No. 6, Deutsche. Grammophon, 1057269, Martha Argerich, 1991	0:00 - 0:25.00
**319***	Fryderyk Chopin	Préludes, Op. 28, No. 2, Deutsche. Grammophon, 1057269, Martha Argerich, 1991	0:28.60 – 0:53.60
**320***	Franz List	Hungarian Rhapsody 1, EMI Classics, 7243 5 75374 2 1, Georges Cziffra, 2002	0:00 - 0:25.00
**321**	Felix Mendelssohn	Lieder ohne Worte, Op. 62, No. 3 in E minor, Trauermarsch, Deutsche Grammophon, 453 061-2, Daniel Barenboim, 1974	0:15.71-0:40.71
**322**	Felix Mendelssohn	Lieder ohne Worte, Op. 102, No. 1 in E minor, Andante, un poco agitato, Deutsche Grammophon, 453 061-2, Daniel Barenboim, 1974	0:00 - 0:25.00
**323**	Fryderyk Chopin	Nocturne No. 21 in C minor, Deutsche Grammophon, 477 0962 GH2, Maria Joao Pires, 1996	0:00 - 0:25.00
**401**	P. I. Tchaikovsky	The Seasons, Op. 37, February: Carnaval, Virgin Classics, 7243 5 45042 2 8, Mikhail Pletnev, 1994	0:00 - 0:25.00
**402***	Robert Schumann	Skizzen für den Pedal, CD6, Track 25, Nuova Era Internazionale, Jörg Demus, 1988	0:00 - 0:25.00
**403**	Johannes Brahms	16 Walzer, Op. 39, No. 13, in B major, Thorofon, CTH 2176, Detlef Kraus, 1992	0:00 - 0:25.00
**404**	Franz Liszt	Sonata in B Minor, Philips, 410 040-2, Alfred Brendel, 1991	7:40.10 - 8:05.10
**405***	Franz Liszt	Sonata in B Minor, Philips, 410 040-2, Alfred Brendel, 1991	0:45.55 - 1:10.55
**406***	Fryderyk Chopin	Scherzo No. 1 in B minor, Op. 20, Brilliant Classics, 99802/6, Alwin Bär, 1998	0:06 - 0:31.00
**407**	Franz Liszt	Ungarische Rhapsodien, Vol. 2, No. 15 in A minor, Naxos, 8.554481, Jeno Jandó, 1998	0:39 - 1:04.00
**408**	Robert Schumann	Piano Sonata in G minor, Op. 22, No. 1, So rasch wie möglich, CBS Records, MK 44569, Murray Perahia, 1987	1:30.28 – 1:55.28
**409**	Robert Schumann	Fantasiestücke, Op. 12, No. 5, In der Nacht, Philips, 434 732-2, Alfred Brendel, 1981	2:04.83 - 2:29.38
**410***	Franz Liszt	Legenden, Franz von Padua, Philips, 410 040-2, Alfred Brendel, 1991	3:49.00 - 4:14.00
**411***	Robert Schumann	Album für die Jugend, Op. 68, I, Knecht Ruprecht, Naxos, 8.555711, Rico Gulda, 2001	0:00.22 - 0:25.22

**412***	Franz Liszt	Die Trauergondel, No. 2, Philips, 410 040-2, Alfred Brendel, 1991	4:30.76 - 4:55.76
**413***	Robert Schumann	1-19 Phantasiestück, Nuova Era, NE 7353, Jörg Demus, 1988	1.29.82 - 1:54.82
**414**	Robert Schumann	Romanzen, Op. 28, Nuova Era, NE 7353, Jörg Demus, 1988	0:09.17 - 0:34.17
**415**	Fanny Mendelssohn-Hensle	Das Jahr, 12 Charakterstücke, No. 4, April, Sony Classical, 88697030162, Lauma Skirde, 2007	1:22.21 - 1:47.12
**416**	Franz Schubert	Drei Klavierstücke (3 Impromptus), D946, No. 2 in E-flat major, Harmonic Records, 8610, Paul Badura-Skoda, 1986	2:28.80 - 2:53.80
**417**	Felix Mendelssohn	Lieder ohne Worte, Op. 38, No. 5 in A minor, Agitato, Deutsche Grammophon, 453 061-2, Daniel Barenboim, 1974	0:41.80 – 1:06.80
**418***	Felix Mendelssohn	Lieder ohne Worte, Op. 53, No. 3 in G minor, Presto agitato, Deutsche Grammophon, 453 061-2, Daniel Barenboim, 1974	0:00 - 0:25.00
**419**	Felix Mendelssohn	Lieder ohne Worte, Op. 85, No. 2 in A minor, Allegro agitato, Deutsche Grammophon, 453 061-2, Daniel Barenboim, 1974	0:16.90 - 0:41.90
**420***	Robert Schumann	Piano Sonata in G minor, 4th movement , Sony Classical, 44569, Murray Perahia, 1986	3:42.00 - 4:07.00
**421***	Johannes Brahms	8 Piano Pieces, Op.76, No. 5, Capriccio in C sharp minor, Agitato, ma non troppo presto, London Records, 430 053-2, Julius Katchen, 1990	0:00 - 0:25.00
**422***	Robert Schumann	Kinderszenen, Op. 15, Wichtige Begebenheit, Tacet, 153, Evgeni Koroliov, 2006	0:00 - 0:25.00
**423**	Franz Schubert	Piano Sonata in A major, 3rd movement, Sony Classical, 44569, Murray Perahia, 1986	0:06.89 - 0:31.89
**Prac1**	Franz Schubert	Impromptu in A flat major, Op. 90, D899, Genuin, GEN 86055, Paul Badura-Skoda, 2005	0:00 - 0:25.00
**Prac2***	Robert Schumann	Album für die Jugend, Op. 68, I, Fröhlicher Landmann von der Arbeit zurückkehrend, Naxos, 8.555711,, Rico Gulda, 2001	0:00 - 0:25.00
