# Supplementary material for: Examining Complexity across Domains: Relating Subjective and Objective Measures of Affective Environmental Scenes, Paintings and Music
Source: PLoS One. 2013 Aug 16;8(8):e72412. doi: 10.1371/journal.pone.0072412 (PMC3745471; doi:10.1371/journal.pone.0072412)
Supplement: Stimulus List S4 — List of 40 Romantic piano chamber music excerpts. (PDF) [file pone.0072412.s019.pdf]

Stimulus List S4. List of 40 Romantic piano chamber music excerpts

The first number of the code refers to the degree of arousal and pleasantness (1 = low-arousing pleasant, 2 = high-arousing pleasant, 3 = low-arousing unpleasant, and 4 = high-arousing unpleasant) based on a pre-selection by the first author. Due to a labeling mistake excerpts No. 102, 201 and 401 were unfortunately taken from a quartet and not from a piano trio, a mistake which was only discovered after the experiment was run.

Code	Composer	Piece & CD	Duration
**101**	Johannes Brahms	Trio No. 3 in C minor, Op. 101, 1st movement, Chandos, 8334, Borodin Trio	1:12.50-1:37.50
**102**	Johannes Brahms	Quartet in A major, Op. 26, 2nd movement, Deutsche Grammophon, 449 611	0:00-0:25.00
**103**	Gabriel Fauré	Trio Op. 120, 1st movement , EMI 0946 336126 2 4	2:15.00-2:40.00
**104**	Gabriel Fauré	Trio, Op. 120, 2nd movement, EMI 0946 336126 2 4	0:00-0:25.00
**105**	Camille Saint-Saëns	Trio in F major, Op. 18, 4th movement, Naxos, 8550935, Joachim Trio	4:39.00-5:04.00
**106**	Camille Saint-Saëns	Trio in E minor, Op. 92, 1st movement, Naxos, 8550935, Joachim Trio	4:25.50-4:50.50
**107**	Franz Schubert	Trio No. 1 in B flat major, Op. 99, D. 898, 1st movement, EMI, 749979 Jess Trio Wien	1:56.00-2:21.00
**108**	Franz Schubert	Trio No. 1 in B flat major, Op. 99, D. 898, EMI, 749979, 2nd movement, Jess Trio Wien	0:08.50-1:33.50
**109**	Franz Schubert	Trio in one movement in B flat, D. 28, Phillips, 426096-2, Beaux Arts Trio	1:03.00-1:28.00
**110**	Johannes Brahms	Trio No. 3 in C minor, Op. 101, 2nd movement, Chandos, 8334, Borodin Trio	2:36.50-3:01.50
**201**	Johannes Brahms	Quartet in A major, Op. 26, 4th movement, Deutsche Grammophon, 449 611-2	0:00-0:25.00
**202**	Louis Spohr	Trio E minor, Op. 119, 1st movement, Orfeo, 352952, Neues Münchner Klaviertrio	1:37.00-2:02.00
**203**	Louis Spohr	Trio E minor, Op. 133, 4th movement, Orfeo, 352952, Neues Münchner Klaviertrio	0:00-0:25.00
**204**	Camille Saint-Saëns	Trio in F-major, Op. 18, 1st movt., Allegro, Naxos, 8550935, Joachim Trio	0:00-0:25.00
**205**	Franz Liszt	Trio in E major (Carneval de Pesth), EMI, 749979, Jess Trio Wien	3:38.50-4:03.50
**206**	Franz Schubert	Trio No. 1 in B flat major, Op. 99, D. 898, 4th movement, EMI, 749979, Jess Trio Wien	2:09.00-2:34.00

**207**	Robert Schumann	Piano Trio No. 1 in D minor, Op. 63, 4th movement, Hyperion, GAW21063, Florestan Trio	4:31.00-4:56.00
**208**	Franz Schubert	Trio No. 2 in E flat major, D. 929, 3rd movement, Phillips, 426096-2, Beaux Arts Trio	2:59.00-3:24.00
**209**	Franz Schubert	Trio No. 2 in E flat major, D. 929, 4th movement, Phillips, 426096-2, Beaux Arts Trio	2:01.50-2:31.50
**210**	Franz Schubert	Trio No. 1 in B flat major, Op. 99, D. 898, EMI, 749979, Jess Trio Wien	2:01.00-2:26.00
**301**	Camille Saint-Saëns	Trio in F major, Op. 18, 2nd movement, Naxos, 8550935, Joachim Trio	0:35.00-1:00.00
**302**	Robert Schumann	Trio No. 1 in D minor, Op. 63, 3rd movement, Hyperion, GAW21063, Florestan Trio	4:04.00-4:29.00
**303**	Robert Schumann	Trio No. 2 in F major, Op. 80, 2nd movement, Hyperion, GAW21063, Florestan Trio	0:00-0:25.00
**304**	Robert Schumann	Trio No. 2 in F major, Op. 80, 3rd movement, Hyperion, GAW21063, Florestan Trio	0:00-0:25.00
**305**	Camille Saint-Saëns	Trio in E minor, Op. 92, 3rd movement, Naxos, 8550935, Joachim Trio	2:54.00-3:19.00
**306**	Louis Spohr	Trio F major, Op. 123, 2nd movement, Orfeo, 352952, Neues Münchner Klaviertrio	0:55.00 - 1:20.00
**307**	Louis Spohr	Trio F major, Op. 133, 3rd movement, Orfeo, 352952, Neues Münchner Klaviertrio	3:46.00 – 4:11.00
**308**	Franz Schubert	Trio No. 2 in E flat major, D. 929, 2nd movement, Phillips 426096-2, Beaux Arts Trio	3:31.00-3:56.00
**309**	Louis Spohr	Trio in A minor, Op. 124, 2nd movement, Orfeo, 352952, Neues Münchner Klaviertrio	0:00 - 0:25.00
**310**	Louis Spohr	Trio in G minor, Op. 142, 2nd movement, Orfeo, 352952, Neues Münchner Klaviertrio	5:36.50-6:01.50
**401**	Johannes Brahms	Quartet in A major, Op. 26, 3rd movement, Deutsche Grammophon, 449 611-2	6:27.00-6:52.00
**402**	Camille Saint-Saëns	Trio No. 2 in E minor, Op. 92, 5th movement, Naxos, 8550935, Joachim Trio	7:14.00-7:39.00
**403**	Robert Schumann	Trio No. 2 in F major, Op. 80, 4th movement, Hyperion, GAW21063, Florestan Trio	2:16.00-2:41.00
**404**	Franz Schubert	Trio No. 2 in E flat major, D. 929, 1st movement, Phillips, 426096-2, Beaux Arts Trio	6:36.50-7:01.50

**405**	Robert Schumann	Trio No. 1 in D minor, Op. 63, 1st movement, Hyperion, GAW21063, Florestan Trio	6:23.00-6:48.00
**406**	Robert Schumann	Trio No. 1 in D minor, Op. 63, 2nd movement, Hyperion, GAW21063, Florestan Trio	0:47.00-1:12.00
**407**	Robert Schumann	Trio No. 2 in F major, Op. 80, 1st movement, Hyperion, GAW21063, Florestan Trio	3:20.00-3:45.00
**408**	Franz Schubert	Trio No. 2 in E flat major, D. 929, 2nd movement, Phillips 426096-2, Beaux Arts Trio	2:49.00-3:14.00
**409**	Louis Spohr	Trio No. 3 in A minor, Op. 124, 4th movement, Orfeo, 352952, Neues Münchner Klaviertrio	2:30.50-3:55.50
**410**	Camille Saint-Saëns	Trio in E minor, Op. 92, 1st movement, Naxos, 8550935, Joachim Trio	6:44.00-7:09.00
**Prac**	Louis Spohr	Trio in F major, Op. 123, 1st movement, Orfeo, 352952, Neues Münchner Klaviertrio	0:00-0:25.00
